# Supplementary material for: Hierarchical incremental learning deciphers molecular arrangements in multi-component materials
Source: Nat Commun. 2025 Oct 22;16:9324. doi: 10.1038/s41467-025-64372-4 (PMC12546701; doi:10.1038/s41467-025-64372-4)
Supplement: Supplementary file 1 — Supplementary Information [file 41467_2025_64372_MOESM1_ESM.pdf]

Supporting Information

Hierarchical incremental learning deciphers molecular arrangements in multi-component materials

Hanyin Zhang,[a,b] *Nan Lin,*[c] *Austin M. Evans,*[d] *Tonghui Wang,*[e] *Saied Md Pratik,*[f] *Jean-Luc Bredas,*[f] and *Haoyuan Li*[a,b*]

[a]School of Microelectronics, Shanghai University, Shanghai 201800, China
[b]Key Laboratory of Advanced Display and System Applications, Ministry of Education, Shanghai University, Shanghai 200072, China
[c]Department of Statistics and Data Science, Washington University, St. Louis, MO 63130, USA
[d]George and Josephine Butler Polymer Laboratory, Department of Chemistry, University of Florida, Gainesville, Florida 32611-7200, United States
[e]School of Materials Science and Engineering, Jilin University, Changchun 130022, China
[f]Department of Chemistry and Biochemistry, The University of Arizona, Tucson, Arizona 85721-0041, United States

*Corresponding author: lihaoyuan@shu.edu.cn

Table of Contents

1. Mathematical descriptions S4

1.1. Simulated data S4

1.2. Data pre-processing S4

1.3. Learning contexts by clustering analysis S5

1.3.1. Clustering local atomic environments corresponding to a single context S5

1.3.2. Clustering local atomic environments corresponding to multiple contexts S6

1.4. Labelling a new local atomic environment S7

1.5. Evaluation of the HiDiscover framework S7

1.6. Selection of tuning parameters S8

2. Molecular dynamics simulations S9

2.1. MD model 1 .... S9

2.2. MD model 2 .... S11

2.3. MD model 3 .... S13

2.4. MD model 4 .... S15

2.5. MD model 5 .... S16

2.6. MD model 6 .... S18

2.7. MD model 7 .... S19

2.8. MD model 8 .... S21

2.9. MD model 9 .... S23

2.10. MD model 10 .... S25

2.11. MD model 11 .... S27

2.12. MD model 12 .... S29

2.13. MD model 13 .... S31

2.14. MD model 14 .... S32

2.15. MD model 15 .... S34

2.16. MD model 16 .... S35

2.17. MD model 17 .... S37

2.18. MD model 18 .... S38

2.19. MD model 19 .... S40

2.20. MD model 20 .... S42

2.21. MD model 21 .... S44

2.22. MD model 22 .... S46

3. Data processing **S48**
4. Model training **S52**
4.1. Li-ion transport in COF-PEO-3 .... **S52**
4.1.1. Task ζ_A **S55**
4.1.2. Task ζ_B **S58**
4.1.3. Task ζ_C **S59**
4.1.4. Task ζ_D **S61**
4.1.5. Accuracies on the test set **S62**
4.2. CO_2 adsorption in MOF-5 .... **S62**
4.2.1. Task ζ_E **S64**
4.2.2. Task ζ_F **S66**
4.2.3. Accuracies on the test set **S67**
4.3. Molecular packing in the active layer of the PM6:Y6 organic solar cell **S67**
4.3.1. Task ζ_G **S68**
4.4. Impact of temporal correlation in datasets **S69**
4.5. Computational costs and benchmarks of the trajectory lengths for the reference molecular models **S72**
5. Additional data analysis **S78**
6. Suggestions for designing the reference molecular systems **S90**
7. References **S90**

1. Mathematical descriptions

We describe below the mathematical treatment in each task of the HiDiscover research protocol.

1.1. Simulated data

We generate multiple datasets using molecular dynamic (MD) simulations according to a set of MD models on a carefully designed schedule to disentangle various atomic configurations (See, *e.g.*, **Figure 2**). The schedule is designed in an incremental nature such that earlier simulations yield single-component or more homogeneous configurations, while later ones produce multi-component systems or a mixture of heterogeneous configurations. For example, the MD model for dataset 1 corresponds to context A_1 only, while that for dataset 2 corresponds to the combination of contexts A_1 and A_2. Note that due to intrinsic constraints in the materials, we cannot easily simulate configurations corresponding to A_2 only. This simulation design then allows incremental learning of both A_1 and A_2. After we learn about A_1 based on dataset 1, we can identify configurations corresponding to A_2 by 'separating' them from those corresponding to A_1 based on our understanding of context A_1 learned from dataset 1.

We take the case of Li-ion transport as an example. Each simulated dataset contains the atomic configuration of a huge single-/multi-component system of Li^+-containing compounds over N_T time frames. The value of N_T is chosen to be sufficiently large to have enough training data so that the HiDiscover method achieves high accuracy. Typically, we use N_T of 100,000. Meanwhile, the time interval Δ between two consecutive frames needs to be reasonably large to decrease the correlation in the training data. Typically, we set Δ = 100 ps. We randomly partition each simulated dataset into a training set, a validation set, and a test set according to an 8:1:1 ratio.

1.2. Data pre-processing

We assume that the atomic configuration of a multi-component system is represented with a fixed underlying grid of atomic sites in a d-dimensional space. Typically, $d = 3$. For the Li-ion transport problem, the simulated system is then summarized by the Li^+-local atomic environments therein. Note that we do not track the temporal dependence of local environments in the training stage of the HiDiscover protocol, so it results in $N = \gamma_{train} n N_T$ local atomic environments for training, where n is the number of Li^+, N_T the output time frames in the simulation, and γ_{train} is the ratio of the training set.

We define a Li^+-local atomic environment as a set of neighboring atoms around a Li-ion. The neighboring atoms are decided by the chemical groups of interest in the specific task. For example, in studying the Li^+-ClO_4^- configurations, we first identify the K nearest neighboring ClO_4^- ions of a Li^+. For each of the K neighboring ClO_4^- ions, we choose the central Cl atom and the nearest O atom to Li^+. Overall, including the center Li^+ ion itself, this results in $2K + 1$ atoms in the local atomic environment. Let $\mathbf{x}_i = (x_{i1},...,x_{id})$ denote the coordinates of the ith atom in the local environment, $i = 1,...,2K + 1$. Indexing of the atoms follows a pre-specified ordering starting from the center Li^+ and followed by other neighboring atoms from the nearest to the furthest. For example, in studying the Li^+-ClO_4^- configurations, the ordering is given by (Li^+, $Cl^{(1)}$,$O^{(1)}$, ...,$Cl^{(K)}$,$O^{(K)}$), where the superscript (k) denotes the kth nearest neighboring ClO_4^-. The

locality size K may vary in different tasks and is chosen to optimize the performance of the overall HiDiscover framework. Further information can be found in **Sections 3** and **4**.

A local atomic environment is then depicted by a Coulomb matrix M, which encodes the atomic species and inter-atomic distances of a finite system in a pair-wise, two-body matrix inspired by the form of the Coulomb potential. The elements of the Coulomb matrix **M** are given by:[1]

$$\mathbf{M}_{ii'} = \begin{cases} 0, & \text{if } i = i' \\ 1/r_{ii'} & \text{if } i \neq i' \end{cases} \tag{S1}$$

where $r_{ii'} = ||\mathbf{x}_i - \mathbf{x}_{i'}||_2$ is the (Euclidean) distance between the ith and i'^{th} atom for i and $i' = 1,...,2K + 1$.

1.3. Learning contexts by clustering analysis

We learn contexts by grouping similar local atomic environments using clustering analysis.[2] Currently, we adopt the k-means clustering algorithm[3] for its simplicity and ease of implementation. In general, one may also adopt other clustering algorithms, such as k-medoid and GMM[4]. It remains a future research topic to study the optimal choice of the clustering algorithm. To prepare for the clustering analysis, we flatten the Coulomb matrix M of each local atomic environment into a vector of size $(2K + 1)^2$ by concatenating its rows.

1.3.1. Clustering local atomic environments corresponding to a single context

If the local atomic environments are believed to be from a single context, for example, in the simulation corresponding to MD model 1, we directly apply the k-means clustering algorithm and define the collection of the k clusters as the context's profile.

Let $\mathbf{v}_1, \mathbf{v}_2, ..., \mathbf{v}_N \in R^{(2K+1)^2}$ denote the vectors resulting from flattening the Coulomb matrix of each of the N local atomic environments from an MD simulation. The k-means clustering algorithm assumes that each data point $\mathbf{v}_i$, $i = 1,...,N$, belongs to a certain cluster C_j, $j = 1,...,k$, and the goal is to partition the data points into k clusters by minimizing the within-cluster variance. The algorithm can be described using the following steps:

1. Given a prespecified value of k (see **Sections 1.6** and **4** for the choice of k), initialize k cluster centroids $\boldsymbol{\mu}_1, \boldsymbol{\mu}_2, ..., \boldsymbol{\mu}_k$ at k randomly chosen data points from $\mathbf{v}_1, \mathbf{v}_2, ..., \mathbf{v}_N$.

2. Assign each data point $\mathbf{v}_i$ to the cluster C_j that minimizes the Euclidean distance:

$$j = \arg\min_{l=1,...,k} \|\mathbf{v}_i - \boldsymbol{\mu}_l\|_2 \tag{S2}$$

3. Update the centroids of each cluster by computing the average of the data points in that cluster:

$$\boldsymbol{\mu}_l = \frac{1}{|C_l|} \sum_{\mathbf{v}_i \in C_l} \mathbf{v}_i \tag{S3}$$

   where $|C_l|$ represents the size of C_l.

4. Calculate the total within-cluster variance by:

$$\text{variance} = \sum_{l} \frac{1}{|C_l|} \sum_{\mathbf{v}_i \in C_l} \|\mathbf{v}_i - \boldsymbol{\mu}_l\|_2^2 \quad \text{(S4)}$$

5. Repeat steps 2-4 until convergence, *i.e.* the absolute change in the total variance between consecutive iterations is smaller than 10^{-3}. The context's profile is then given by the set of centroids $\{\boldsymbol{\mu}_1, \boldsymbol{\mu}_2, \ldots, \boldsymbol{\mu}_k\}$.

1.3.2. Clustering local atomic environments corresponding to multiple contexts

In our simulation design, MD models used in later simulations correspond to multiple contexts. For example, MD model 2 corresponds to both contexts A_1 and A_2. A direct application of k-means clustering to the dataset from such a simulation cannot distinguish clusters belonging to different contexts. Therefore, we adopt an incremental learning framework to identify new contexts based on previously defined contexts from earlier simulations. To ensure the identifiability of new contexts, we carefully design our simulation schedule such that an MD model corresponding to multiple contexts involves only a single new context A, and profiles of the rest (denoted by $\tilde{A}$) have all been studied in previous simulations. Profile of the new context A is then defined using the following incremental clustering algorithm through a slight modification to the standard k-means clustering algorithm in **Section 1.3.1**. That is, we fix the centroids of the clusters in the context set $\tilde{A}$ based on the profiles identified in earlier simulations and no longer update them during the iterations of the clustering process. Details of the algorithm are as follows.

Suppose that the set $\tilde{A}$ of existing contexts contains $\tilde{k}$ clusters defined by their centroids $\boldsymbol{\mu}_1, \boldsymbol{\mu}_2, \ldots, \boldsymbol{\mu}_{\tilde{k}}$. Again, let $\mathbf{v}_1, \mathbf{v}_2, \ldots, \mathbf{v}_N \in R^{(2K+1)^2}$ be the flattened Coulomb matrices from an MD simulation.

1. Given a prespecified value of k, initialize k cluster centroids $\boldsymbol{\mu}_{\tilde{k}+1}, \boldsymbol{\mu}_{\tilde{k}+2}, \ldots, \boldsymbol{\mu}_{\tilde{k}+k}$ at k randomly chosen data points from $\mathbf{v}_1, \mathbf{v}_2, \ldots, \mathbf{v}_N$.
2. Assign each data point $\mathbf{v}_i$ to the cluster C_j that minimizes the Euclidean distance:

$$j = \arg\min_{l=1,\ldots,\tilde{k}+k} \|\mathbf{v}_i - \boldsymbol{\mu}_l\|_2 \quad \text{(S5)}$$

3. Update the centroids of each new cluster by computing the average of the data points in that cluster:

$$\boldsymbol{\mu}_l = \frac{1}{|C_l|} \sum_{\mathbf{v}_i \in C_l} \mathbf{v}_i, \text{ for } l = 1,2,\ldots,\tilde{k}+k \quad \text{(S6)}$$

4. Calculate the total within-cluster variance by:

$$\text{variance} = \sum_{l} \frac{1}{|C_l|} \sum_{\mathbf{v}_i \in C_l} \|\mathbf{v}_i - \boldsymbol{\mu}_l\|_2^2, \text{ for } l = 1,2,\ldots,\tilde{k}+k \quad \text{(S7)}$$

5. Repeat steps 2-4 until convergence (the same criterion in **Section 1.3.1** is used). The profile of the new context A is then given by the set of centroids $\{\boldsymbol{\mu}_{\tilde{k}+1}, \boldsymbol{\mu}_{\tilde{k}+2}, ..., \boldsymbol{\mu}_{\tilde{k}+k}\}$

1.4. Labelling a new local atomic environment

Suppose that we learned M contexts from the previous incremental learning framework and the mth context contains k_m clusters, whose centroids are $\{\boldsymbol{\mu}_{m1}, \boldsymbol{\mu}_{m2}, ..., \boldsymbol{\mu}_{mk_m}\}$, $m = 1,...,M$. Then, we can use them to classify a new local atomic environment by matching it to the context profiles. Let $\mathbf{v}_0$ be the flattened Coulomb matrix of the new local atomic environment. We first classify it into the cluster with the closest centroid:

$$\arg\min_{m=1,...M;l=1,...,k_m} \left\| \mathbf{v}_0 - \boldsymbol{\mu}_{ml} \right\|_2 \tag{S8}$$

We then assign $\mathbf{v}_0$ to the corresponding context m containing that cluster.

1.5. Evaluation of the HiDiscover framework

If a validation dataset V is from an MD simulation model corresponding to a single context m, the true context label for each local atomic environment is obviously just m. Then, we evaluate the performance (accuracy) of the HiDiscover framework by the correct classification rate (CCR):

$$\text{CCR} = \frac{1}{|V|}\sum_{v=1}^{|V|} 1\left(A_v = m\right) \tag{S9}$$

where |V| is the number of local atomic environments in the validation dataset, A_v is the predicted context label of the vth local atomic environment given by the HiDiscover method, and $1(S)$ is the indicator function whose value is 1 if S is true and 0 otherwise.

On the other hand, when a validation dataset is from an MD simulation model corresponding to multiple contexts, we can not assign a single true context label to each local atomic environment but only know it is from one of a set of contexts, say $\tilde{A}$. For example, in MD simulation model 2, $\tilde{A} = A_1 \cup A_2$. We then compute the CCR as

$$\text{CCR} = \frac{1}{|V|}\sum_{v=1}^{|V|} 1\left(A_v \in \tilde{A}\right) \tag{S10}$$

where V is the validation dataset, and A_v is the predicted context label of the vth local atomic environment given by the HiDiscover method. In practice, we use Ω_m as $\tilde{A}$ to evaluate the CCR for label m.

We may assess the uncertainty of a model on a dataset by the entropy of the softmax output. High entropy indicates high uncertainty and the application of the model on this dataset should be taken cautiously. We can apply a trained model to a dataset and compute the entropy for sample points. Given a probability distribution over C classes, the entropy of a vector $\boldsymbol{v}$ (derived from the local atomic environment) is defined as:

$$H(\mathbf{v}) = -\sum_{k=1}^{C} P(k \mid \mathbf{v}) \log P(k \mid \mathbf{v}) \tag{S11}$$

where $P(k|\mathbf{v})$ is the posterior probability of cluster k given $\mathbf{v}$. Using Bayes' theorem, the probability that $\mathbf{v}$ belongs to cluster k is given by:

$$P(k \mid \mathbf{v}) = \frac{P(\mathbf{v} \mid k) P(k)}{P(\mathbf{v})} \tag{S12}$$

where $P(k)$ is the prior probability of cluster k; $P(\mathbf{v}|k)$ is the likelihood of $\mathbf{v}$ under cluster k, modeled as a Gaussian:

$$P(\mathbf{v} \mid k) = \frac{1}{(2\pi)^{d/2} |\Sigma_k|^{1/2}} \exp\left(-\frac{1}{2} (\mathbf{v} - \boldsymbol{\mu}_k)^T \Sigma_k^{-1} (\mathbf{v} - \boldsymbol{\mu}_k) \right) \tag{S13}$$

where $\boldsymbol{\mu}_k$ is the kth centroid and $\boldsymbol{\Sigma}_k$ is the covariance of cluster k; $|\boldsymbol{\Sigma}_k|$ is the determinant of $\boldsymbol{\Sigma}_k$. In the incremental learning framework, the distribution of data may shift across different datasets. The covariance and prior probability are thus calculated from the classified clusters on the evaluated dataset.

The marginal probability $P(\mathbf{v})$ is expressed as follows:

$$P(\mathbf{v}) = \sum_{k=1}^{C} P(\mathbf{v} \mid k) P(k) \tag{S14}$$

Sample points are selected from the dataset excluding those used in the calculation of the covariance and prior probability, for which entropy can be calculated. The normalized entropy is expressed as:

$$H_{norm}(\mathbf{v}) = \frac{H(\mathbf{v})}{\log C} \tag{S15}$$

where $H_{norm}(\mathbf{v})$ ranges from 0 to 1. We average the value of $H_{norm}(\mathbf{v})$ for the sample points as a final indicator.

1.6. Selection of tuning parameters

The performance of our HiDiscover framework depends on a few sets of tuning parameters, especially the number of clusters in defining the context profiles. Let k_m denote the number of clusters for the mth context, m=1,...,M. Ideally, we would like to choose $\{k_1, ..., k_M\}$ jointly by minimizing the overall accuracy of the HiDiscover framework. However, this requires a large number of evaluations on a grid set over different values of $\{k_1, ..., k_M\}$, which is computationally infeasible for even moderate M.

We lower the computational cost using a heuristic sequential approach. Assume that the ordering in $\{k_1, ..., k_M\}$ follows the order in which the contexts were learned. That is, contexts learned earlier are numbered lower. As earlier MD models are simpler than the later ones in our design, it is generally true that the CCR corresponding to an MD simulation model depends on only $\{k_1, ..., k_{\tilde{m}}\}$ but not $\{k_{\tilde{m}+1}, ..., k_M\}$ for some $1 < \tilde{m} < M$. The value of $\tilde{m}$ increases as the MD model becomes more complex. Hence, rather than computing an overall accuracy for the entire HiDiscover system, we use the MD model-specific CCRs to choose the number of clusters sequentially. The entropy values are used as a reference. After the number of clusters for an earlier context is decided, we will fix it when choosing that for a later context. For example, in task ζ_A, we first determined the value of k_1 to be 20 and then fixed k_1 when proceeding to further contexts (**Figures S78-S80**).

Sometimes, the CCR might be relatively insensitive to the number of clusters, so we choose the number of clusters that gives less variability in the classification result to achieve better stability for the HiDiscover framework. If neither CCR nor the variability can distinguish between several choices on the number of clusters, we randomly choose one from the equally good choices. Details can be found in **Section 4**.

2. Molecular dynamics simulations

2.1. MD model 1

An 8×6×5 supercell of $LiClO_4$ was initially constructed (960 $LiClO_4$, 3.83 nm × 4.03 nm × 4.29 nm). NPT simulation was performed to bring the system to 298 K over 0.5 ns and kept at this temperature for another 19.5 ns. A production run was then performed for 100 ns under NVT conditions, which was used for further processing to generate the dataset.

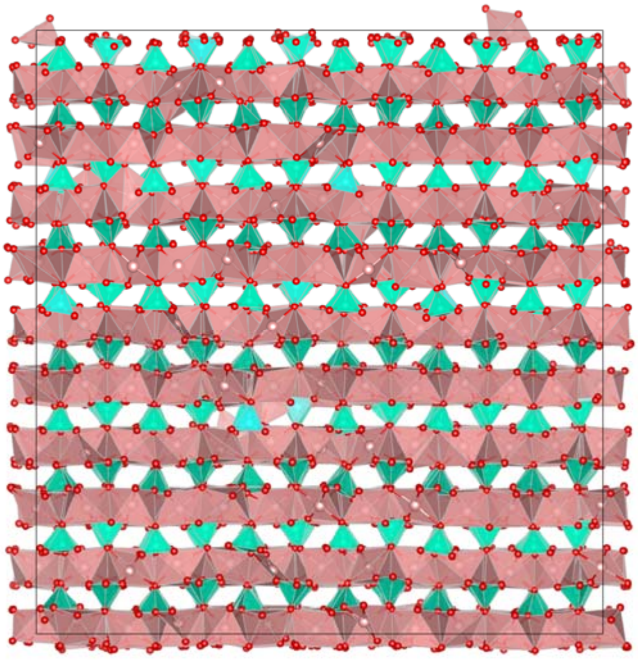

Figure S1. Illustration of the structure of MD model 1.

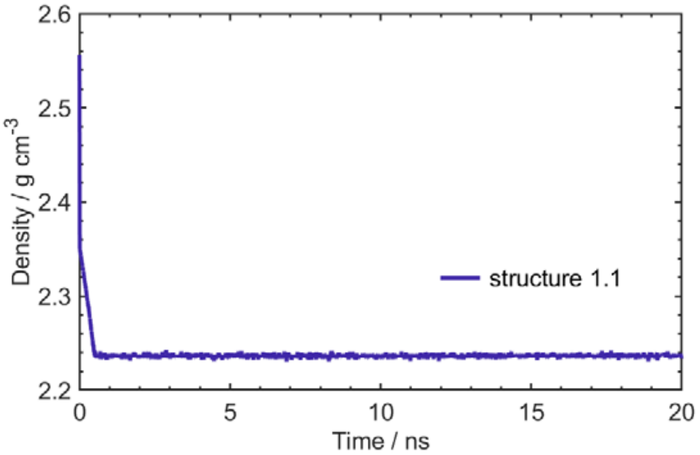

Figure S2. Simulated density of MD model 1 at different times.

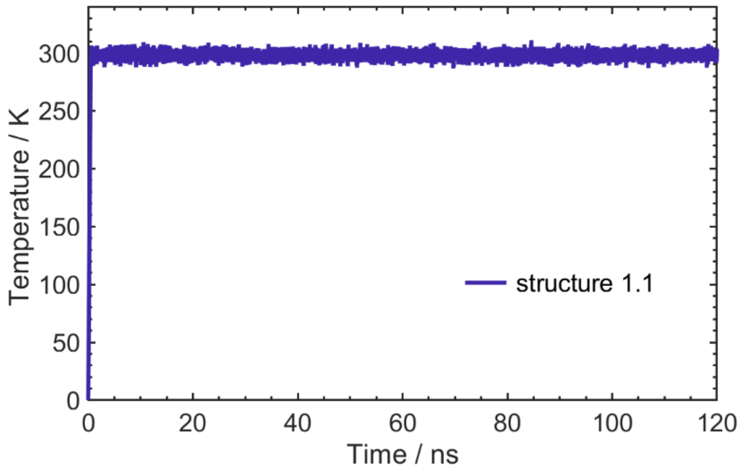

Figure S3. Simulated temperature of MD model 1 at different times.

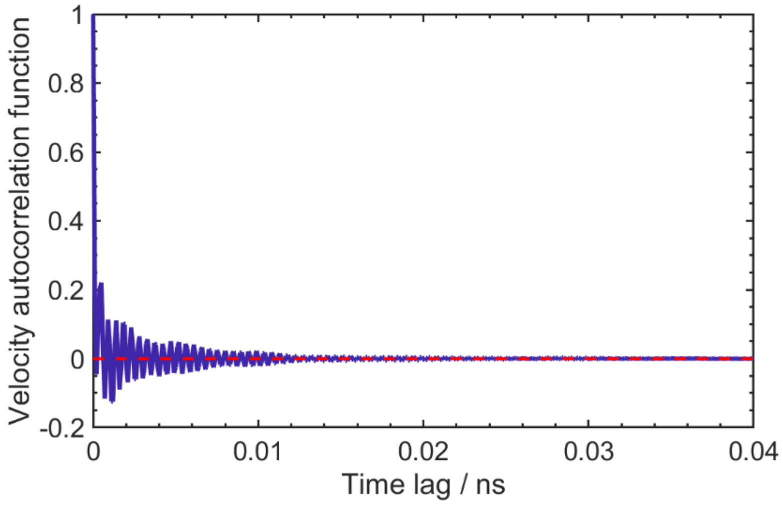

Figure S4. Velocity autocorrelation functions at different time lags of MD model 1.

2.2. MD model 2

1000 $LiClO_4$ were initially randomly placed in a box of 4.5 nm × 4.5 nm × 4.5 nm using packmol (version 20.3.5)[5]. Four parallel models were constructed, denoted as structures 2.1-2.4. NPT simulation was performed to bring the system to 298 K over 0.5 ns and kept at this temperature for another 19.5 ns. The density of the systems was stable after 2 ns. NVT simulation was then performed for 100 ns, and the last 50 ns were further processed to generate the dataset.

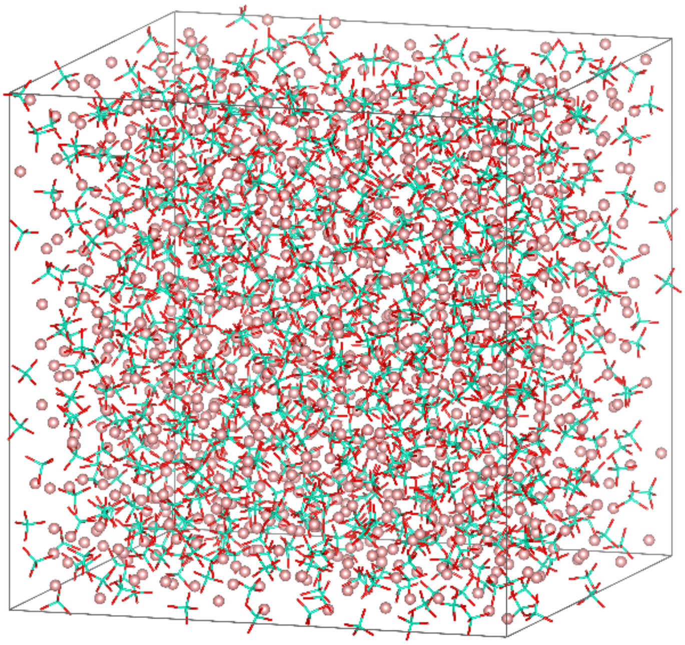

Figure S5. Illustration of the structure of MD model 2.

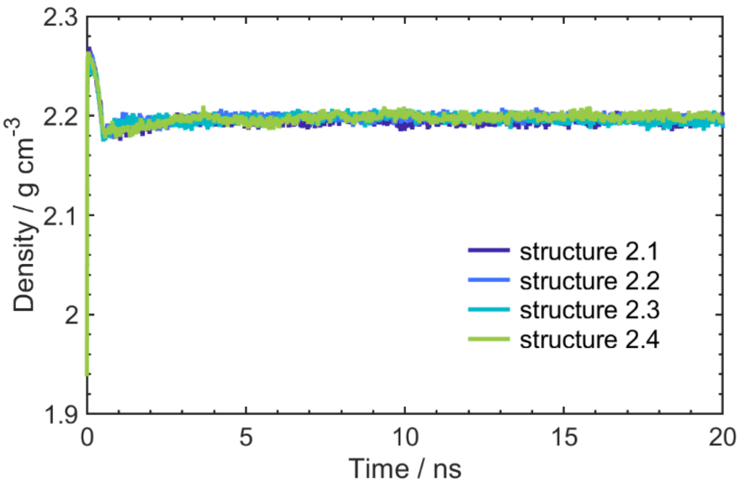

Figure S6. Simulated density of MD model 2 at different times.

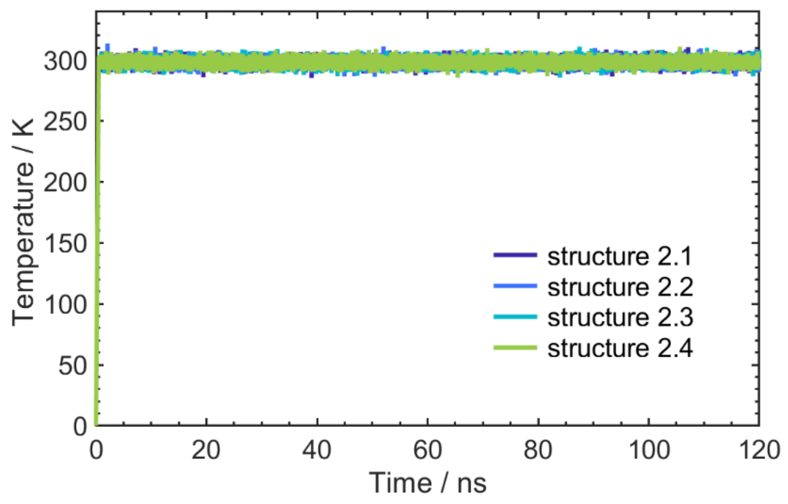

Figure S7. Simulated temperature of MD model 2 at different times.

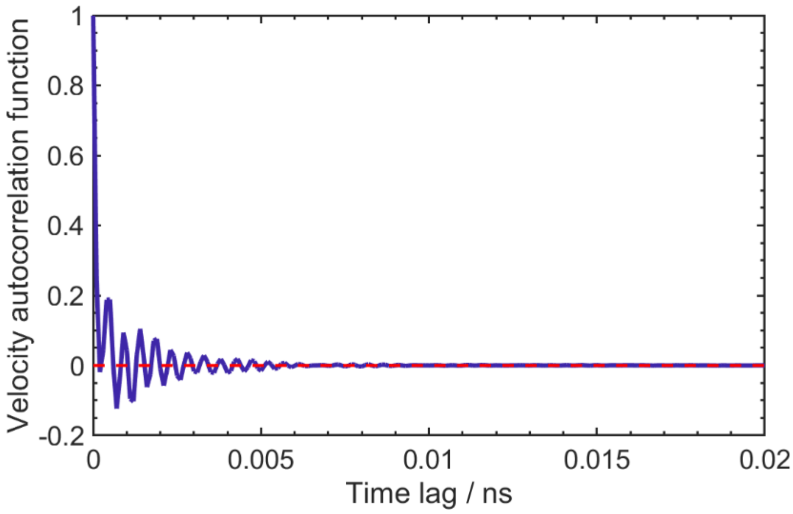

Figure S8. Velocity autocorrelation functions at different time lags of MD model 2.

2.3. MD model 3

The final molecular structures of Model 2 were put into a box of 9 nm × 9 nm × 9 nm. These were denoted as structures 3.1-3.4. NVT simulation was performed to bring the system to 298 K over 0.5 ns and kept at this temperature for another 49.5 ns. A production run was then performed for 100 ns, the data of which was further processed to generate the dataset.

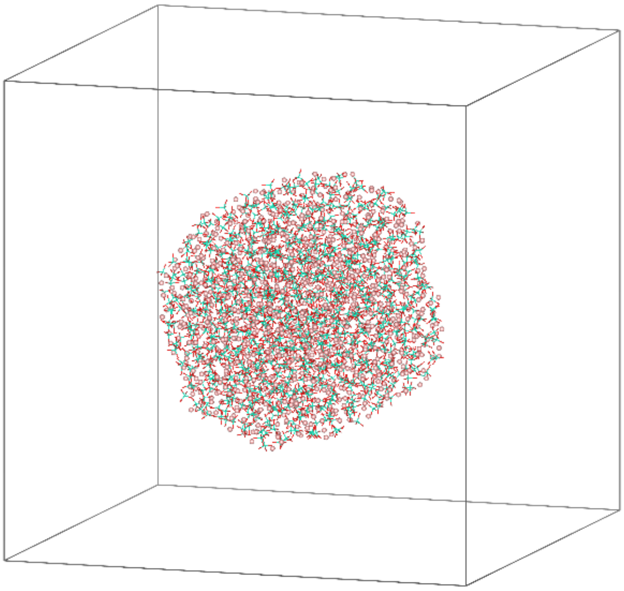

Figure S9. Illustration of the structure of MD model 3.

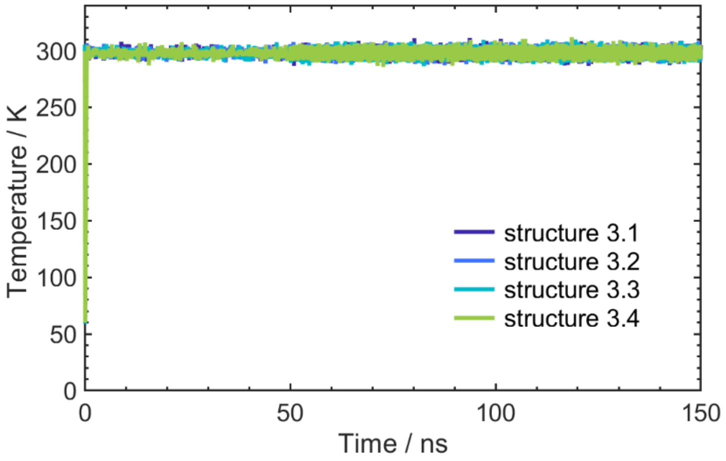

Figure S10. Simulated temperature of MD model 3 at different times.

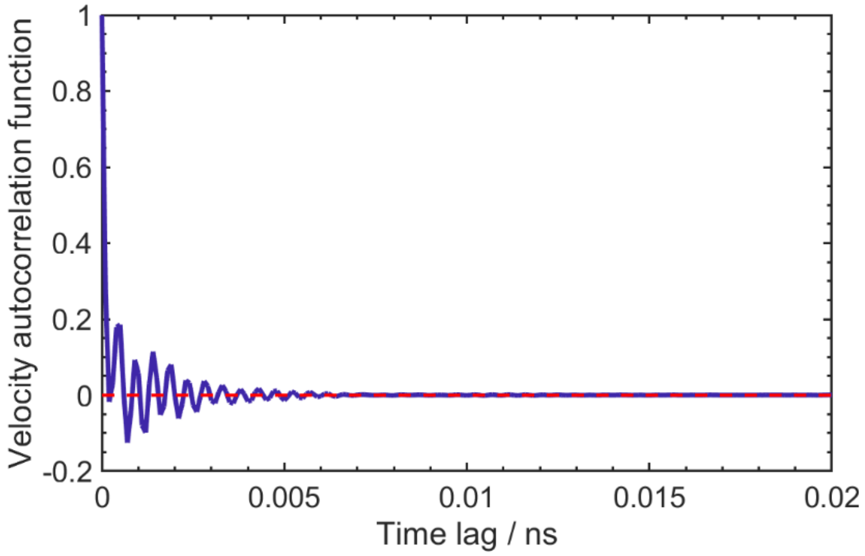

Figure S11. Velocity autocorrelation functions at different time lags of MD model 3.

2.4. MD model 4

60 $LiClO_4$ (corresponding to Li^+ ratios of 0.46 wt%) and 2×2×20 supercells of COF-42 were mixed using packmol[5]. Four parallel models were constructed, denoted as structures 4.1-4.4. NVT simulation was performed to bring the system to 298 K over 0.5 ns, which was kept at this temperature for 1500 ns. The last 1000 ns was used for further processing to generate the dataset.

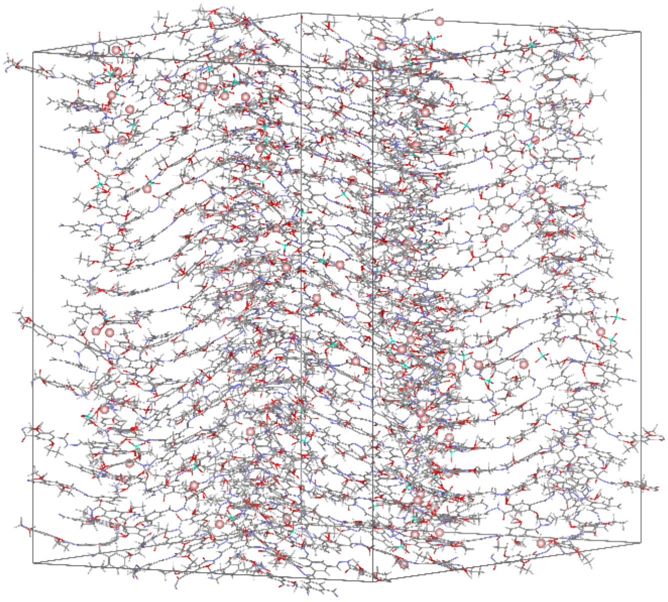

Figure S12. Illustration of the structure of MD model 4.

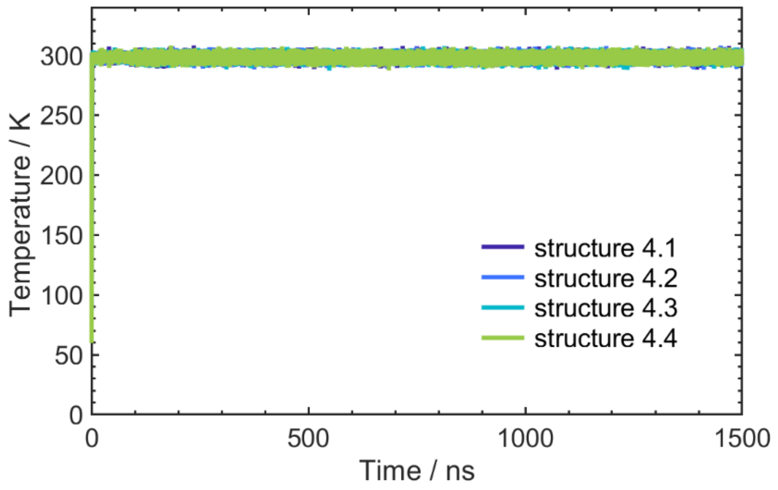

Figure S13. Simulated temperature of MD model 4 at different times.

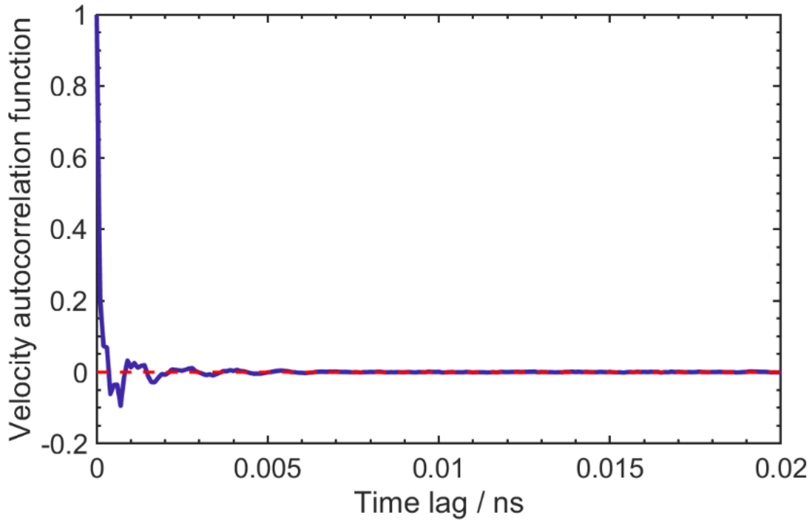

Figure S14. Velocity autocorrelation functions at different time lags of MD model 4.

2.5. MD model 5

30 $LiClO_4$ (corresponding to Li^+ ratios of 0.24 wt%) and 2×2×20 supercells of COF-42 were mixed using packmol[5]. Four parallel models were constructed, denoted as structures 5.1-5.4. NVT simulation was performed to bring the system to 298 K over 0.5 ns, which was kept at this temperature for 1500 ns. The last 1000 ns was used for further processing to generate the dataset.

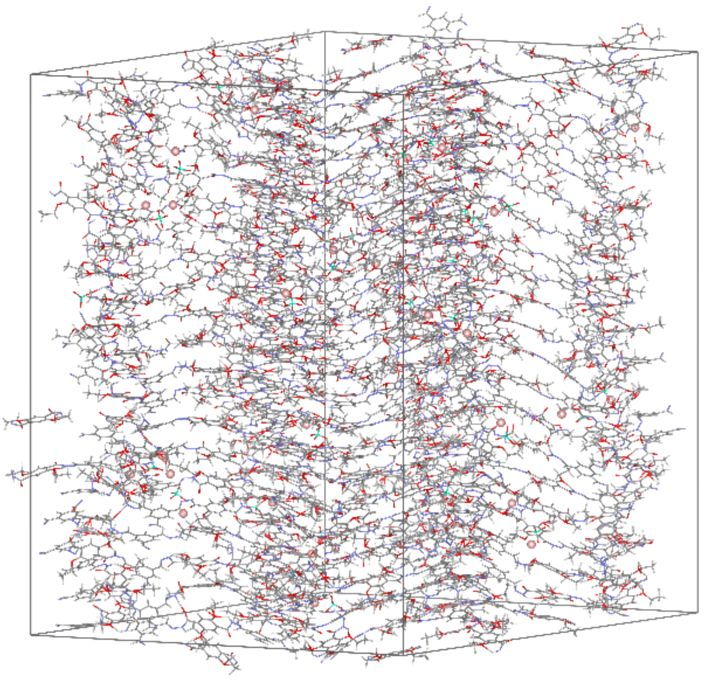

Figure S15. Illustration of the structure of MD model 5.

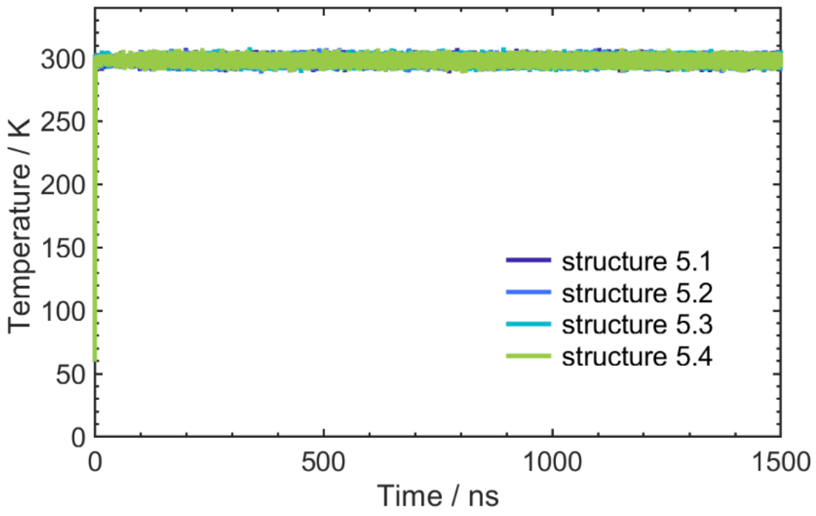

Figure S16. Simulated temperature of MD model 5 at different times.

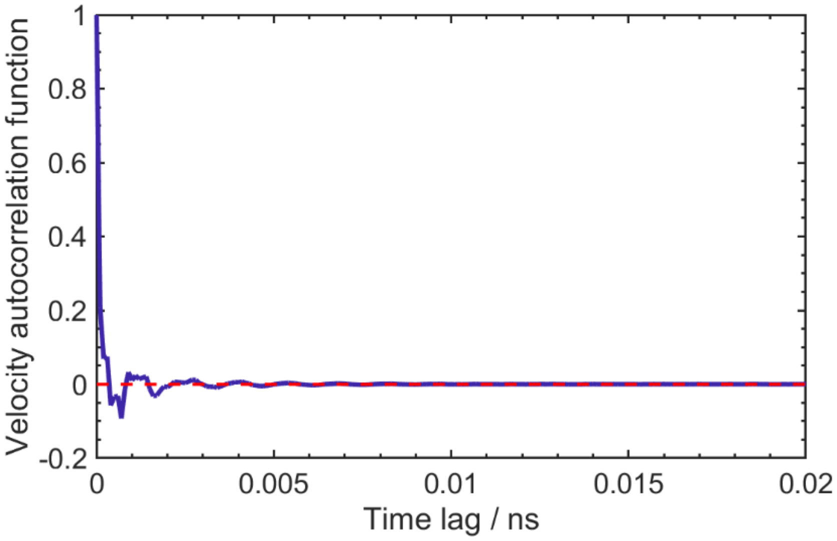

Figure S17. Velocity autocorrelation functions at different time lags of MD model 5.

2.6. MD model 6

240 $LiClO_4$ (corresponding to Li^+ ratios of 1.5 wt%) and 2×2×20 supercells of COF-42 were mixed using packmol[5]. Four parallel models were constructed, denoted as structure 6.1-6.4. NVT simulation was performed to bring the system to 298 K over 0.5 ns, which was kept at this temperature for 1500 ns. The last 1000 ns was used for further processing to generate the dataset.

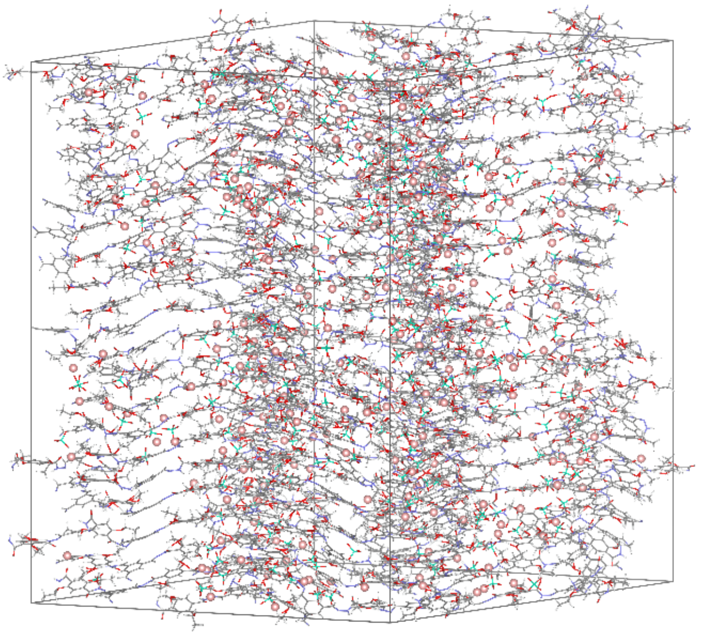

Figure S18. Illustration of the structure of MD model 6.

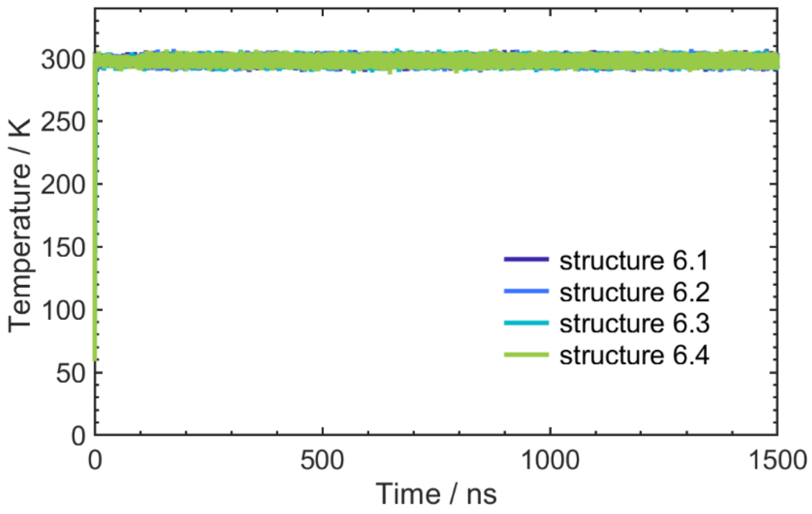

Figure S19. Simulated temperature of MD model 6 at different times.

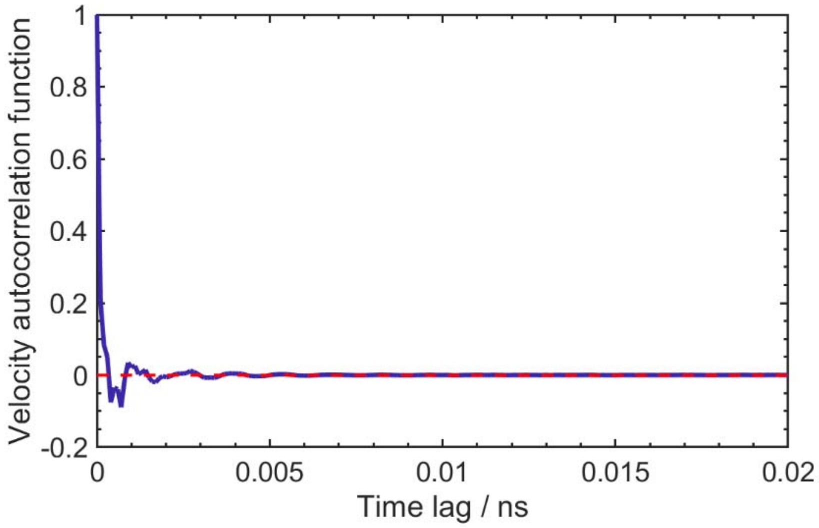

Figure S20. Velocity autocorrelation functions at different time lags of MD model 6.

2.7. MD model 7

60 $LiClO_4$ and 480 $(PEO)_3$ were initially randomly placed in a box of 6 nm × 6 nm × 6 nm using packmol[5]. Four parallel models were constructed, denoted as structures 7.1-7.4. The density of the systems was stable after 1 ns. NPT simulation was performed to bring the system to 298 K over 0.5 ns and kept at this temperature for another 19.5 ns. The system was kept at this temperature for 600 ns under NVT conditions, and the last 500 ns was used for further processing to generate the dataset.

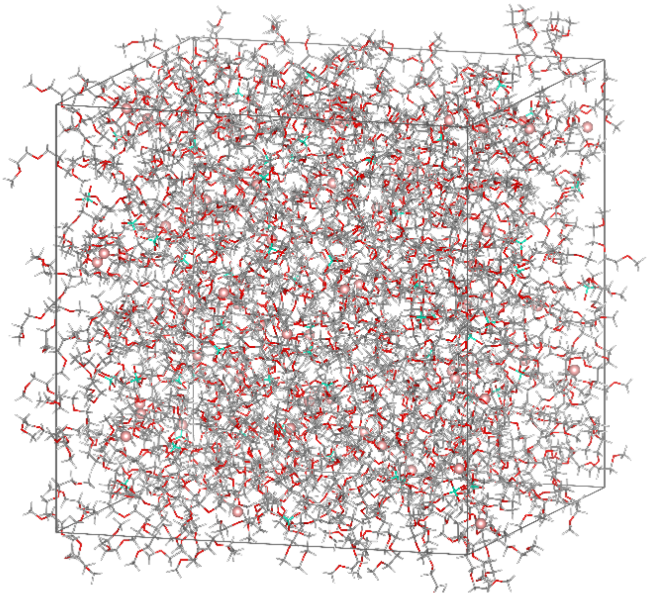

Figure S21. Illustration of the structure of MD model 7.

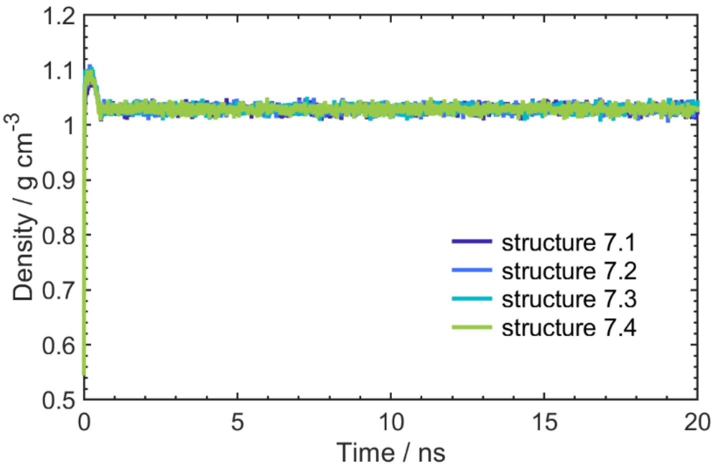

Figure S22. Simulated density of MD model 7 at different times.

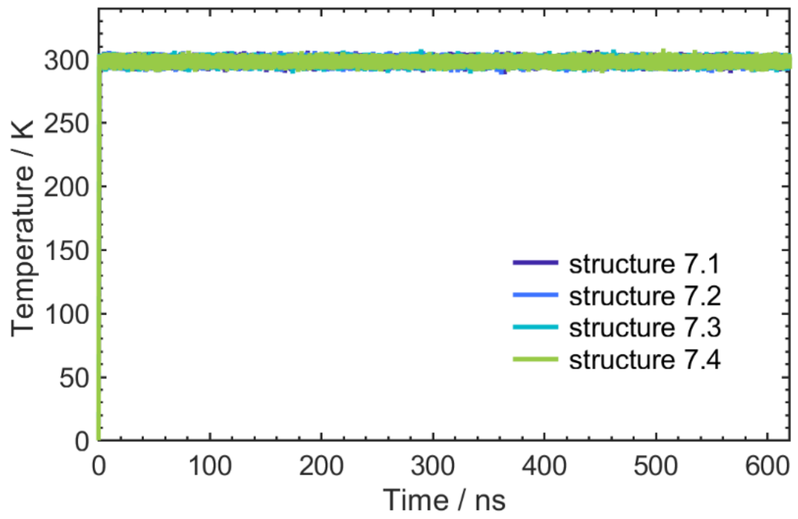

Figure S23. Simulated temperature of MD model 7 at different times.

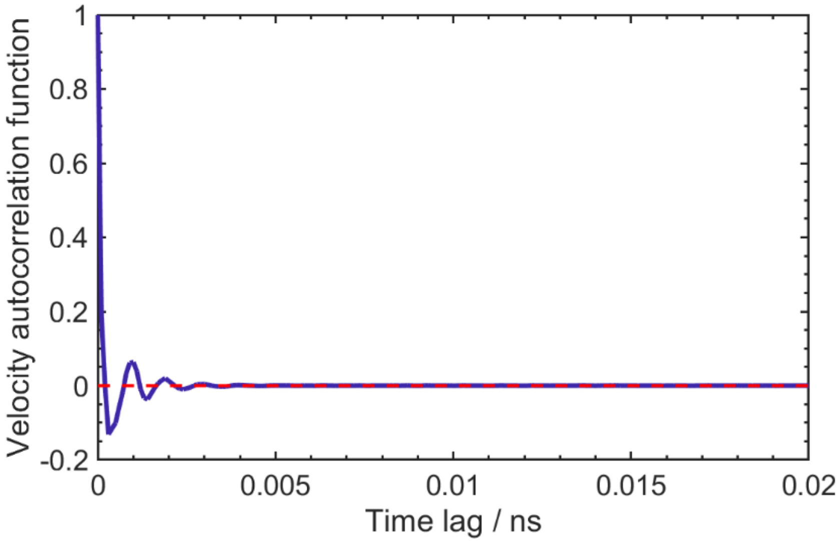

Figure S24. Velocity autocorrelation functions at different time lags of MD model 7.

2.8. MD model 8

360 $LiClO_4$ were initially randomly blended in a 2×2×20 supercell of COF-PEO-3 using packmol[5]. Four parallel models were constructed, denoted as structures 8.1-8.4. NVT simulation was performed to bring the system to 298 K over 0.5 ns. The system was kept at this temperature for 1500 ns, and the last 1000 ns was used for further processing to generate the dataset.

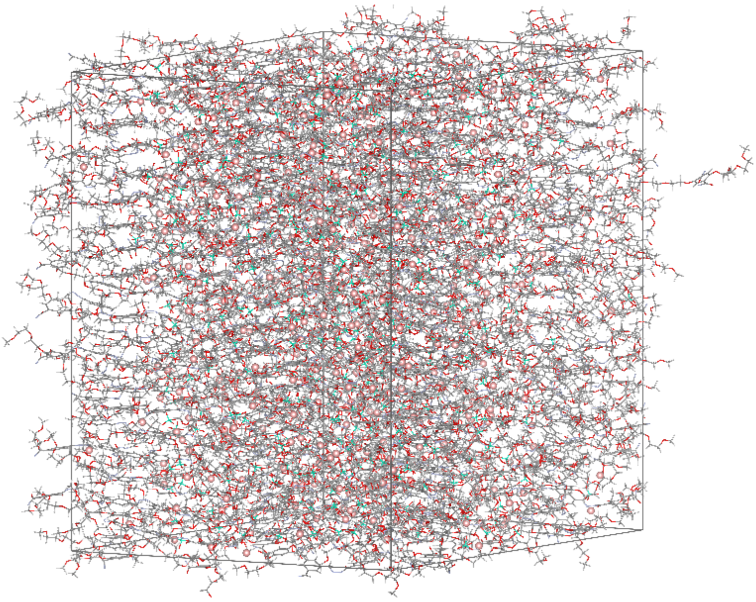

Figure S25. Illustration of the structure of MD model 8.

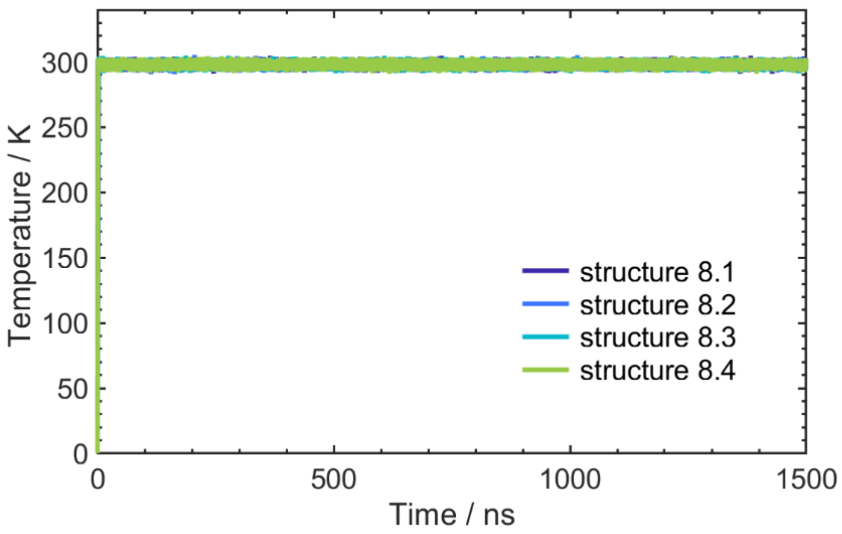

Figure S26. Simulated temperature of MD model 8 at different times.

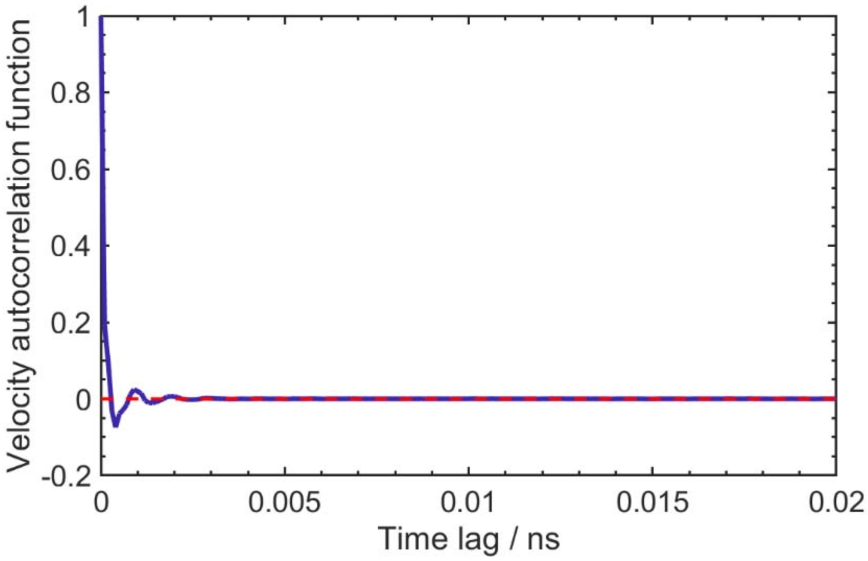

Figure S27. Velocity autocorrelation functions at different time lags of MD model 8.

2.9. MD model 9

1000 CO_2 were initially randomly placed in a box using packmol[5]. The pressure was set to 1000 bars. NPT simulation was performed for 200 ns at 298 K. The density converges after about 30 ns. An NVT production run was then performed for 100 ns, which was used for further processing to generate the dataset.

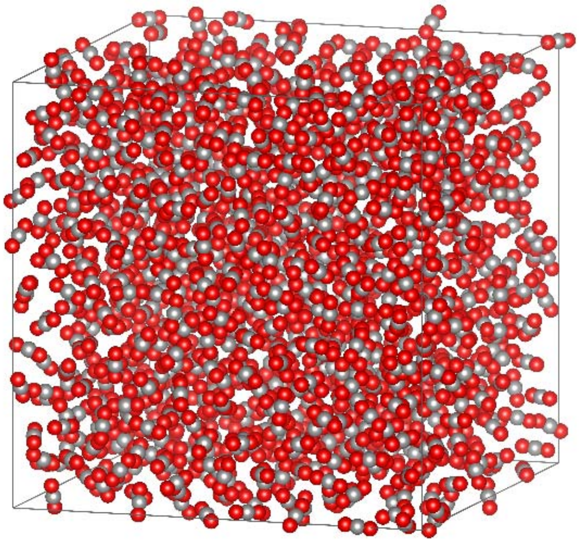

Figure S28. Illustration of the structure of MD model 9.

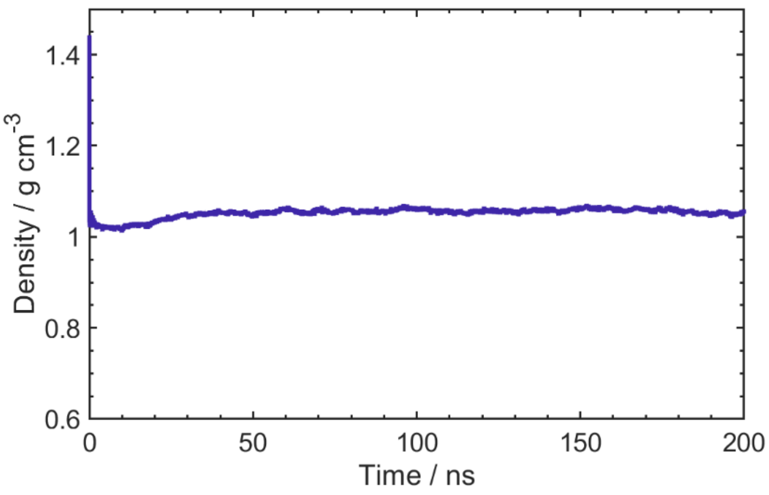

Figure S29. Simulated density of MD model 9 at different times.

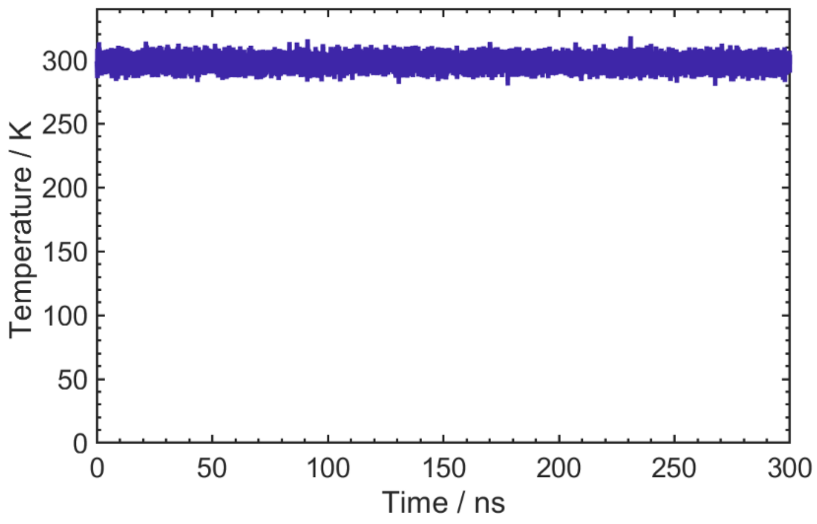

Figure S30. Simulated temperature of MD model 9 at different times.

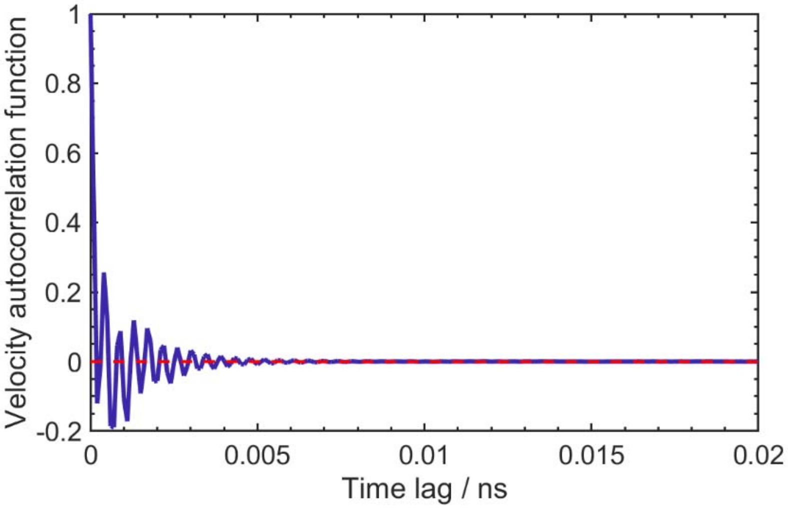

Figure S31. Velocity autocorrelation functions at different time lags of MD model 9.

2.10. MD model 10

1000 CO_2 were initially randomly placed in a box using packmol[5]. The pressure was set to 100 bars. NPT simulation was performed for 100 ns at 298 K. The density converges after about 30 ns. An NVT production run was then performed for 100 ns, which was used for further processing to generate the dataset.

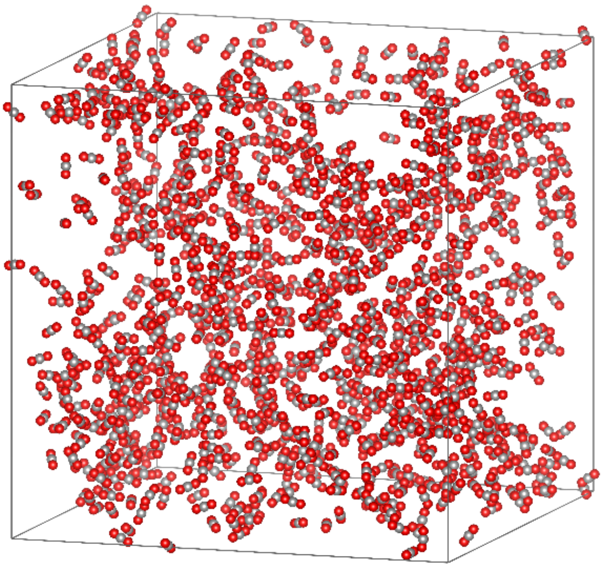

Figure S32. Illustration of the structure of MD model 10.

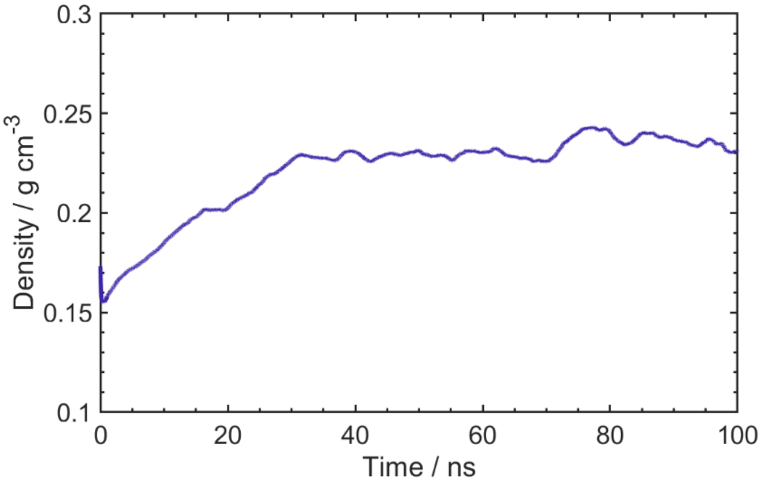

Figure S33. Simulated density of MD model 10 at different times.

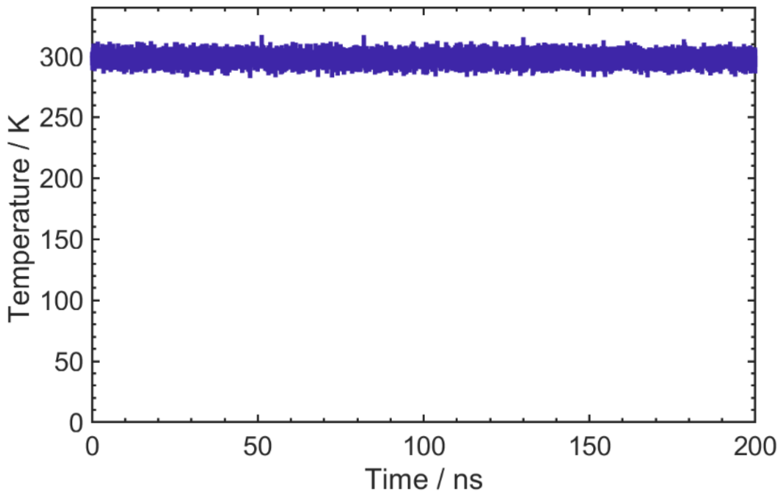

Figure S34. Simulated temperature of MD model 10 at different times.

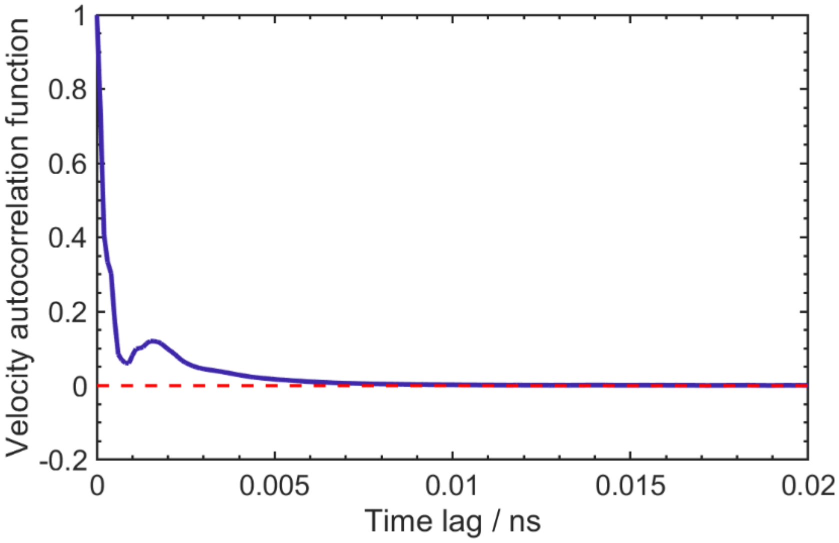

Figure S35. Velocity autocorrelation functions at different time lags of MD model 10.

2.11. MD model 11

1000 CO_2 were initially randomly placed in a box using packmol[5]. The pressure was set to 10 bars. NPT simulation was performed for 300 ns at 298 K. The density converges after about 30 ns. An NVT production run was then performed for 100 ns, which was used for further processing to generate the dataset.

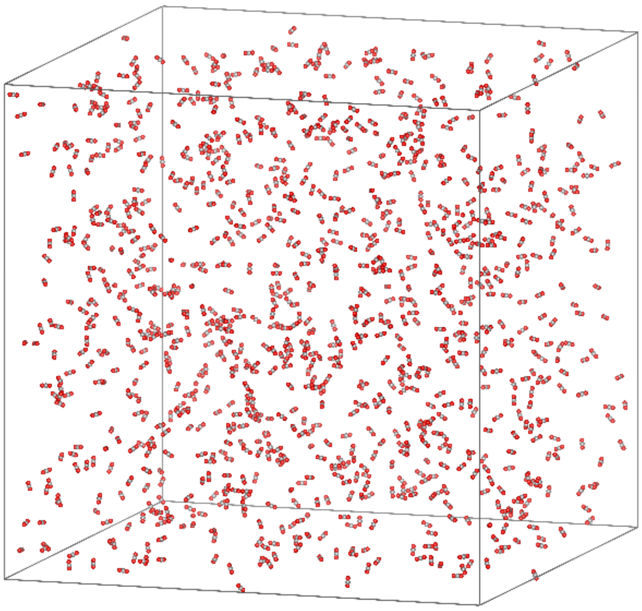

Figure S36. Illustration of the structure of MD model 11.

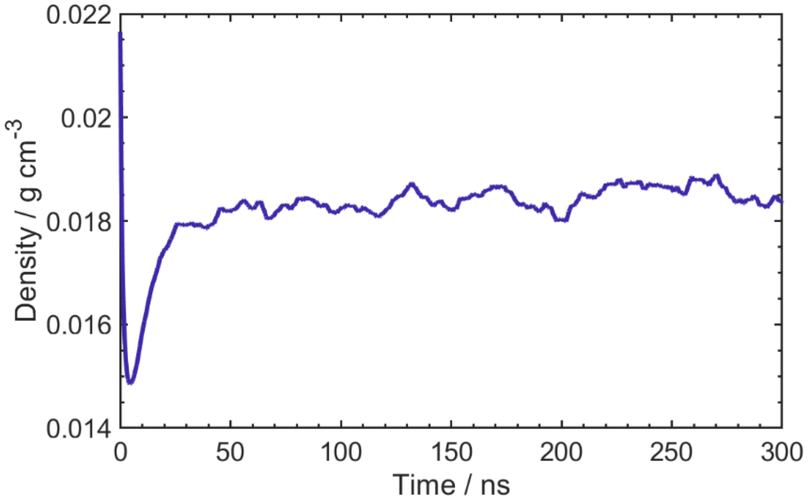

Figure S37. Simulated density of MD model 11 at different times.

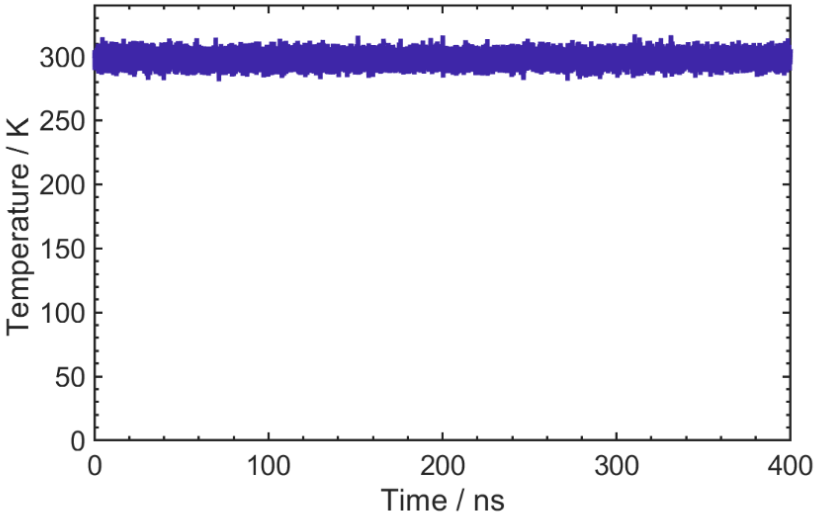

Figure S38. Simulated temperature of MD model 11 at different times.

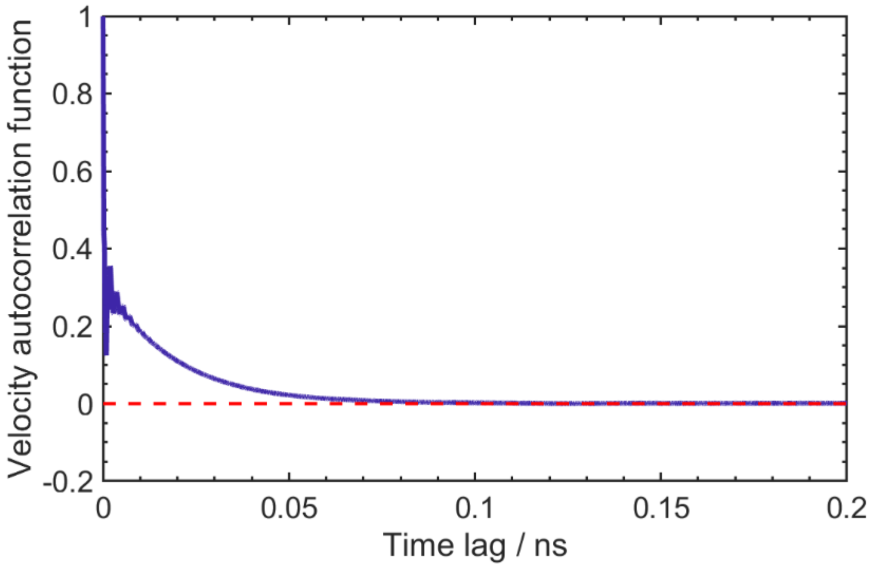

Figure S39. Velocity autocorrelation functions at different time lags of MD model 11.

2.12. MD model 12

1000 CO_2 were initially randomly placed in a box using packmol[5]. The pressure was set to 1 bar. NPT simulation was performed for 300 ns at 298 K. The density converges after about 100 ns. An NVT production run was then performed for 100 ns, which was used for further processing to generate the dataset.

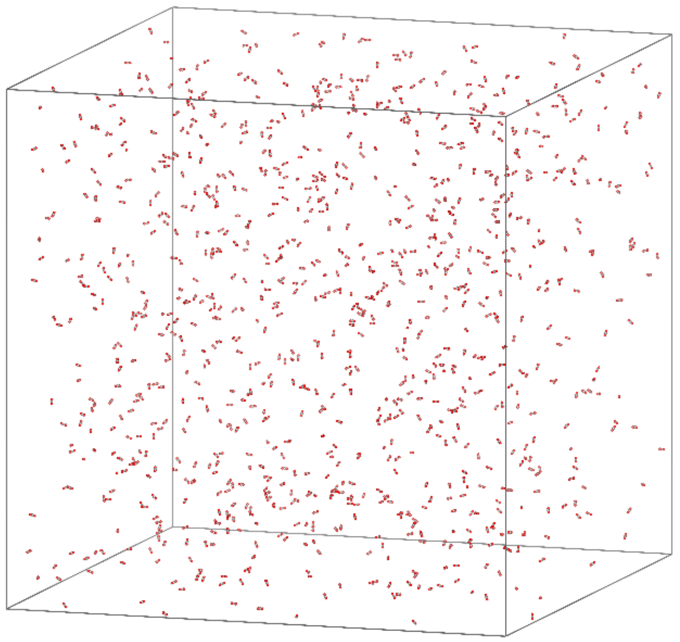

Figure S40. Illustration of the structure of MD model 12.

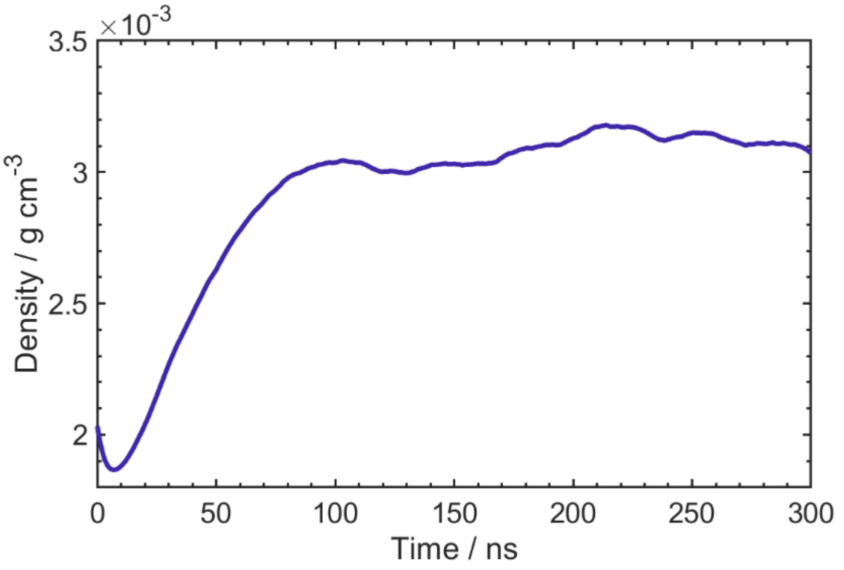

Figure S41. Simulated density of MD model 12 at different times.

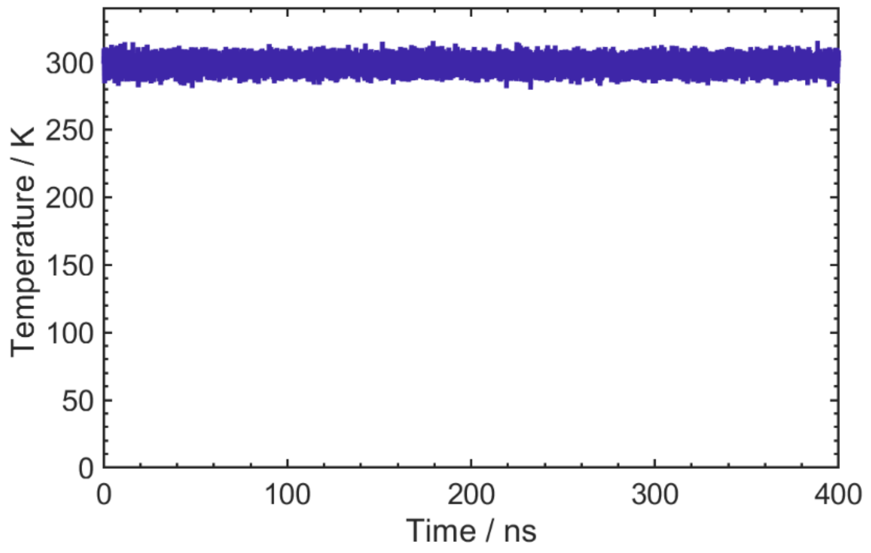

Figure S42. Simulated temperature of MD model 12 at different times.

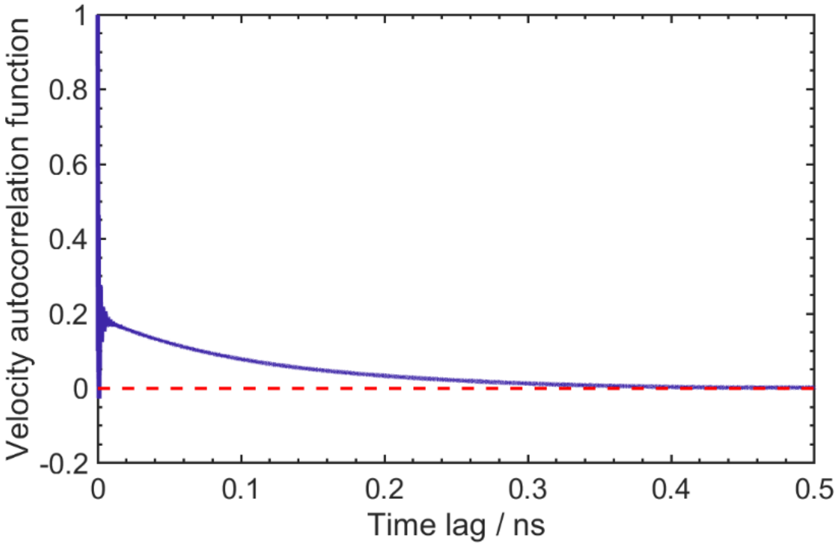

Figure S43. Velocity autocorrelation functions at different time lags of MD model 12.

2.13. MD model 13

25 CO_2 were initially randomly put in a 2×2×2 supercell MOF-5 using packmol[5], corresponding to a loading of 0.5 mmol/g. NVT simulation was performed to bring the system to 298 K over 0.5 ns. The system was simulated at 298 K for 50 ns. Another simulation of 100 ns was performed, which was used for further processing to generate the dataset.

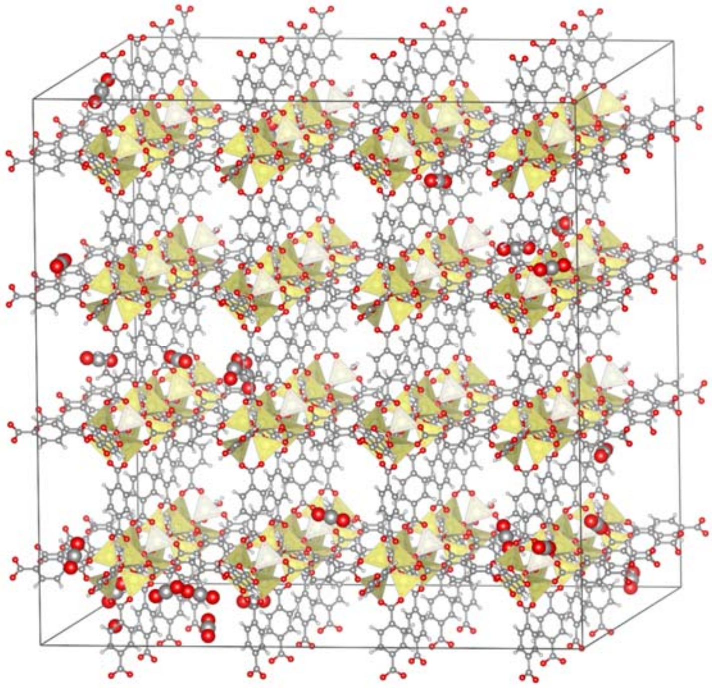

Figure S44. Illustration of the structure of MD model 13.

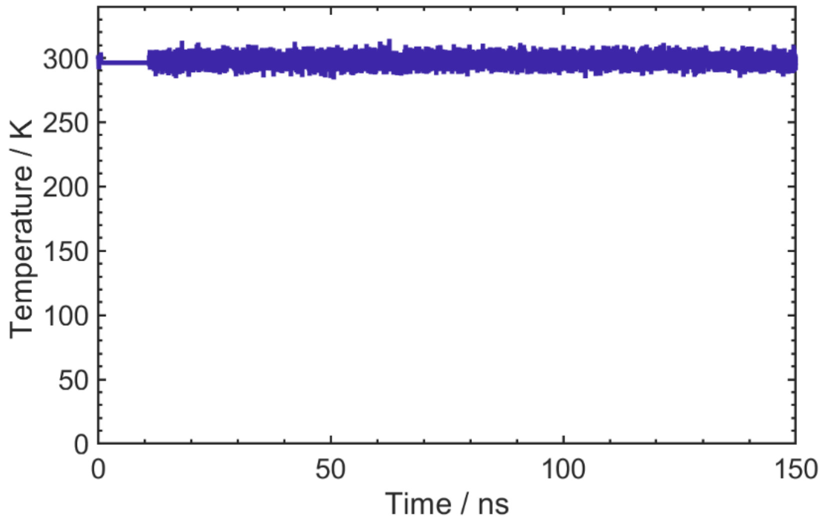

Figure S45. Simulated temperature of MD model 13 at different times.

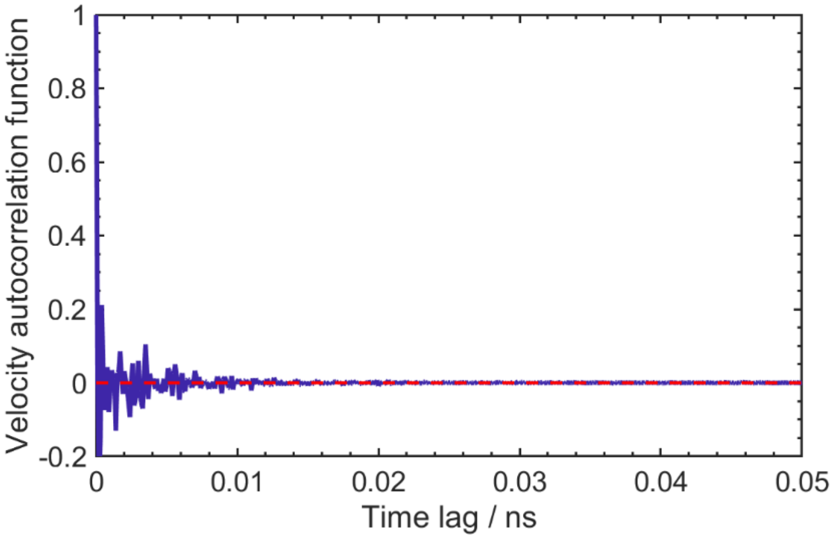

Figure S46. Velocity autocorrelation functions at different time lags of MD model 13.

2.14. MD model 14

99 CO_2 were initially randomly put in a 2×2×2 supercell MOF-5 using packmol[5], corresponding to a loading of 2 mmol/g. NVT simulation was performed to bring the system to 298 K over 0.5 ns. The system was simulated at 298 K for 50 ns. Another simulation of 100 ns was performed, which was used for further processing to generate the dataset.

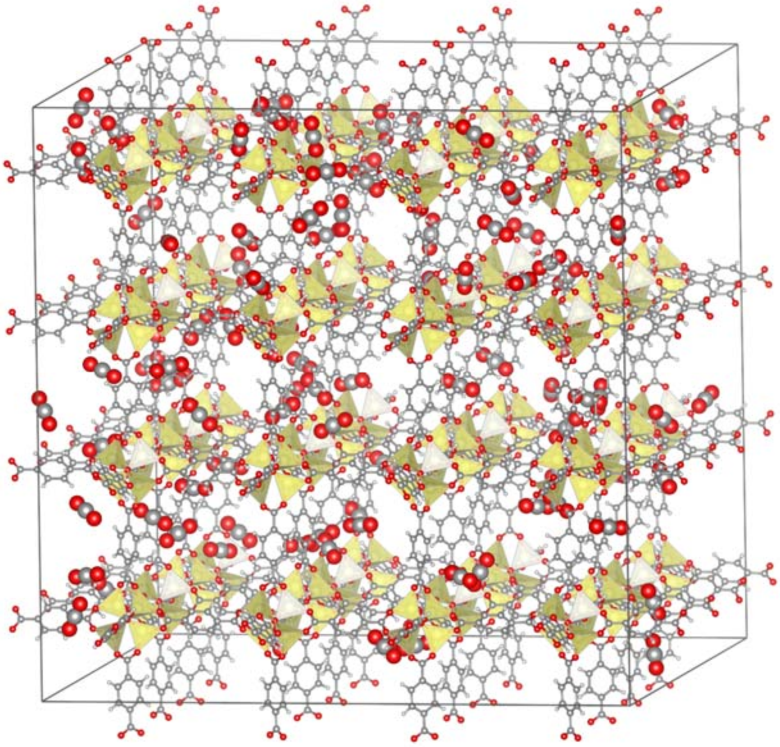

Figure S47. Illustration of the structure of MD model 14.

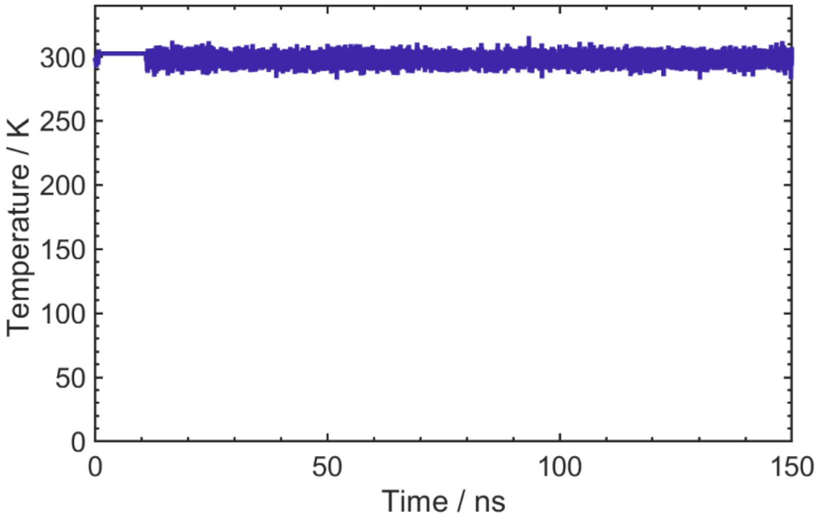

Figure S48. Simulated temperature of MD model 14 at different times.

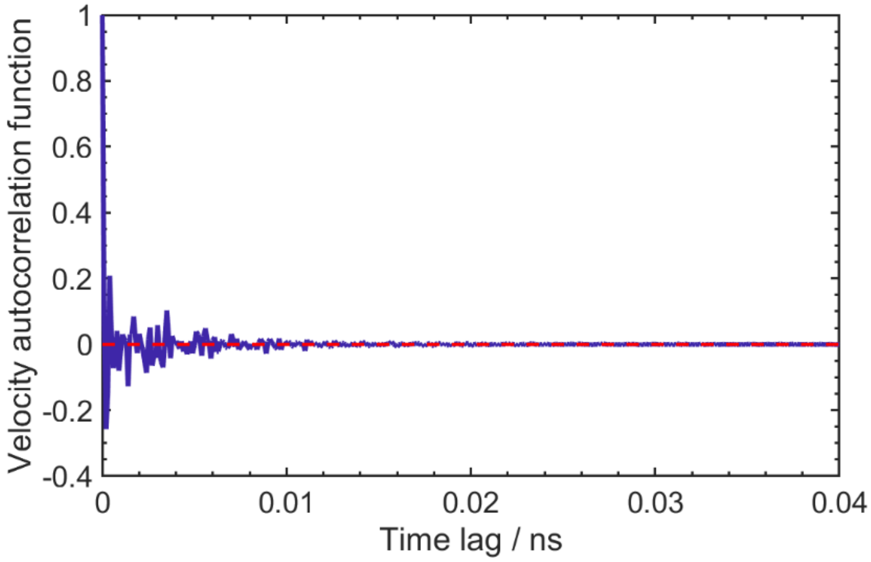

Figure S49. Velocity autocorrelation functions at different time lags of MD model 14.

2.15. MD model 15

493 CO_2 were initially randomly put in a 2×2×2 supercell MOF-5 using packmol[5], corresponding to a loading of 10 mmol/g. NVT simulation was performed to bring the system to 298 K over 0.5 ns. The system was simulated at 298 K for 50 ns. Another simulation of 100 ns was performed, which was used for further processing to generate the dataset.

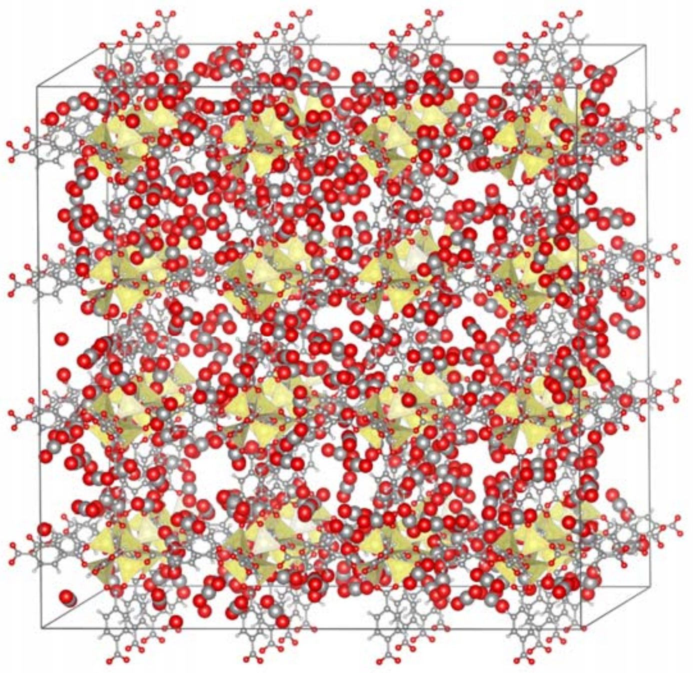

Figure S50. Illustration of the structure of MD model 15.

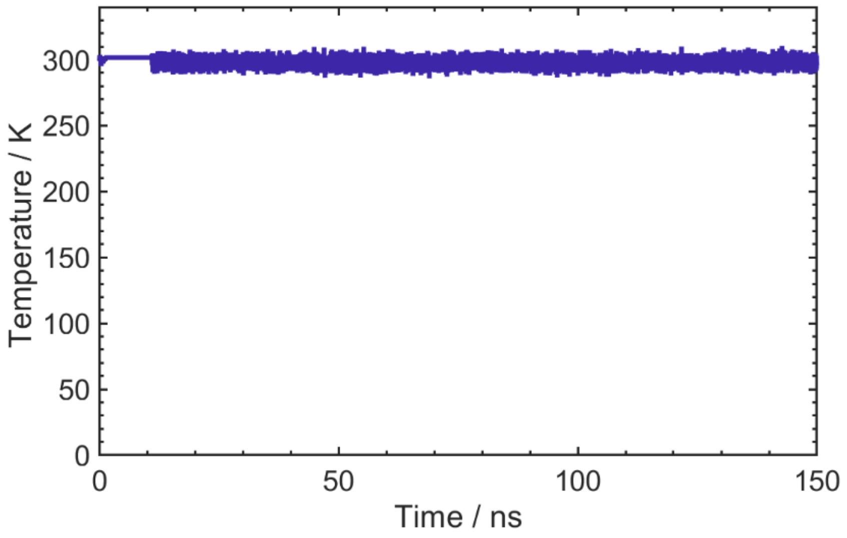

Figure S51. Simulated temperature of MD model 15 at different times.

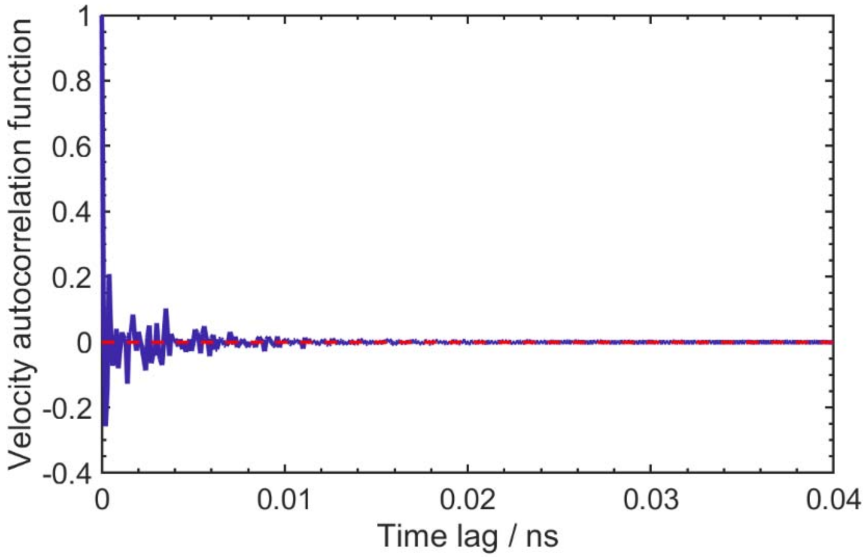

Figure S52. Velocity autocorrelation functions at different time lags of MD model 15.

2.16. MD model 16

985 CO_2 were initially randomly put in a 2×2×2 supercell MOF-5, corresponding to a loading of 20 mmol/g. NVT simulation was performed to bring the system to 298 K over 0.5 ns. The system was simulated at 298 K for 50 ns. Another simulation of 100 ns was performed, which was used for further processing to generate the dataset.

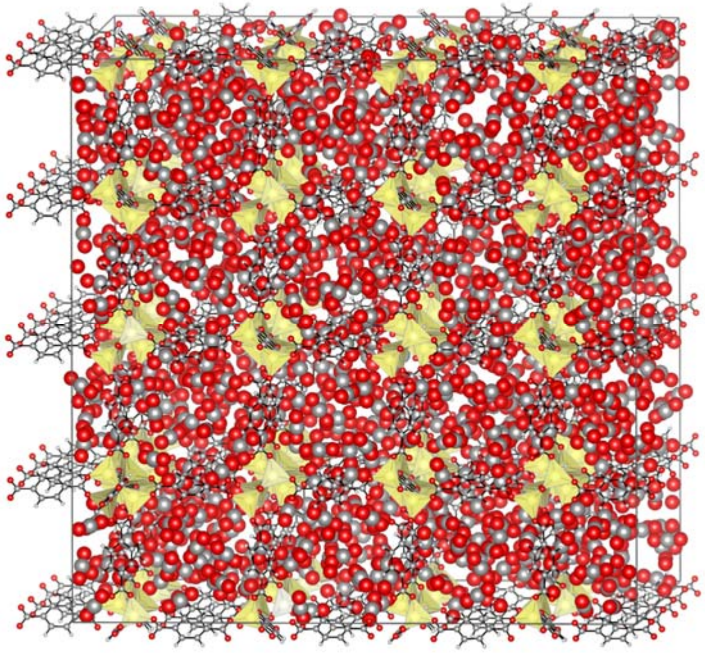

Figure S53. Illustration of the structure of MD model 16.

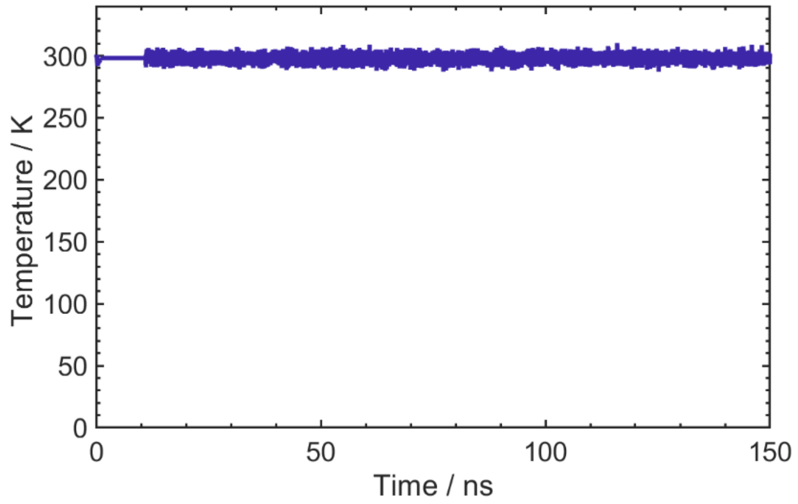

Figure S54. Simulated temperature of MD model 16 at different times.

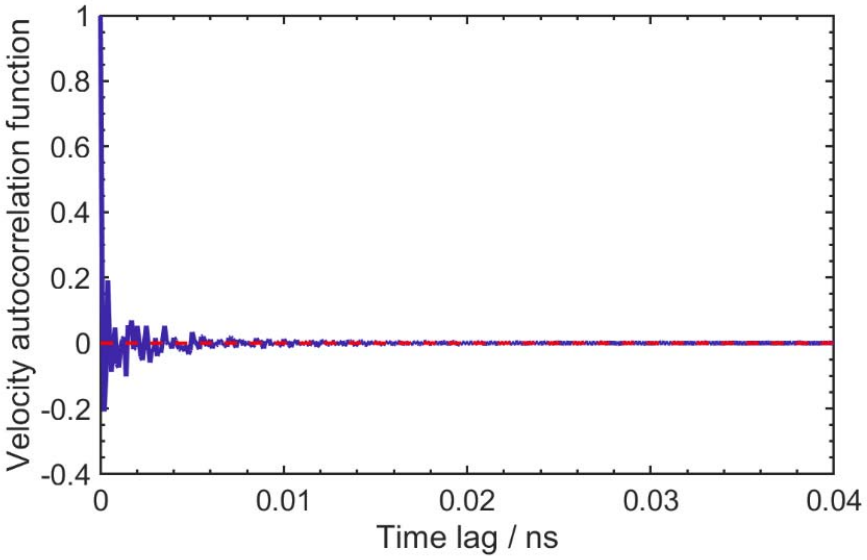

Figure S55. Velocity autocorrelation functions at different time lags of MD model 16.

2.17. MD model 17

This system is a 2×2×4 supercell of Y6 based on its crystal structure.[40] The system contains 256 Y6 molecules. The trajectory for the dataset was generated from a series of geometry optimization steps (steepest descend algorithm) from an initially tilted configuration, which produces coordinates around the equilibrium location. Only the last 700 steps around the equilibrium geometries are used to reduce the impact of the initial structure.

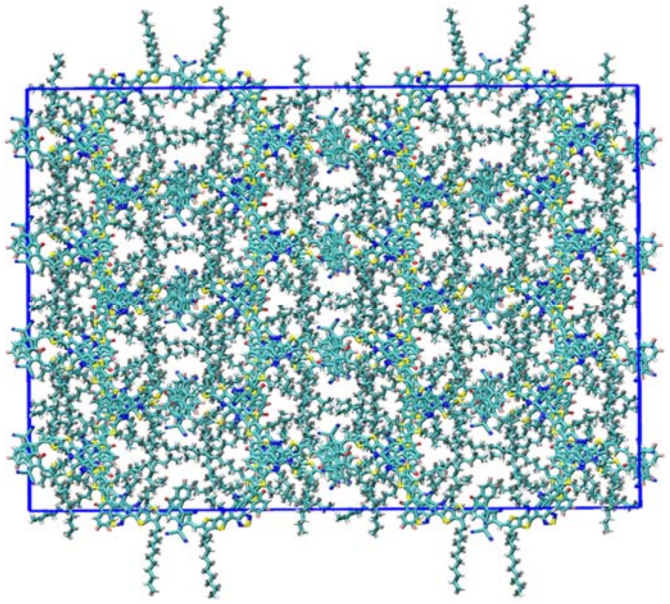

Figure S56. Illustration of MD model 17.

2.18. MD model 18

100 Y6 were initially randomly placed in a cubic box using packmol[5]. Eight parallel models were constructed, denoted as structures 18.1-18.8. NPT simulation was performed to bring the system to 850 K over 1 ns and kept at this temperature for another 49 ns. The system was then brought to 298 K over 100 ns and kept at this temperature for another 100 ns. NVT simulation was then performed for 250 ns, and the last 200 ns was used for further processing to generate the dataset.

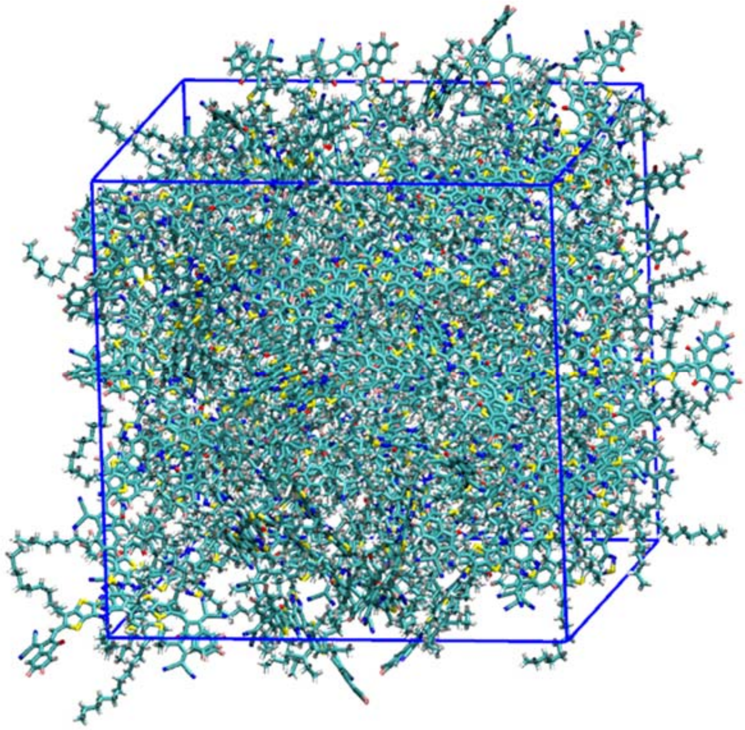

Figure S57. Illustration of MD model 18.

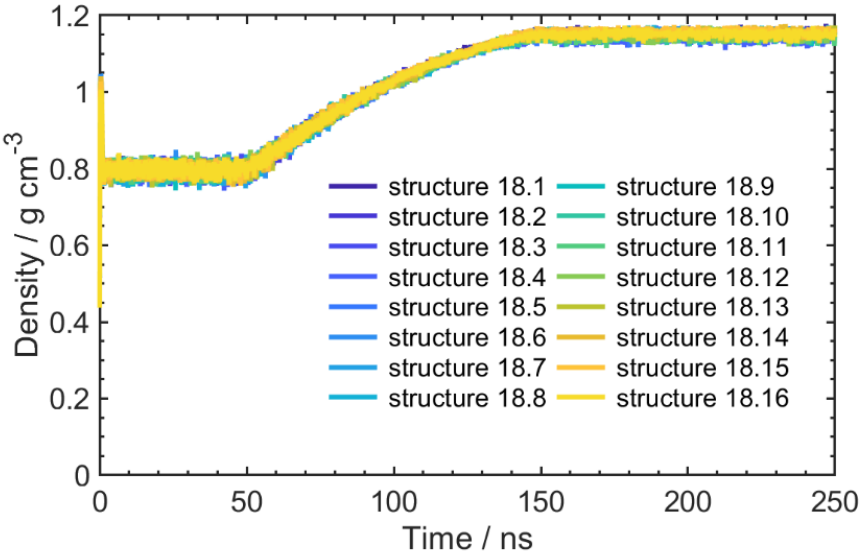

Figure S58. Simulated density of MD model 18 at different times.

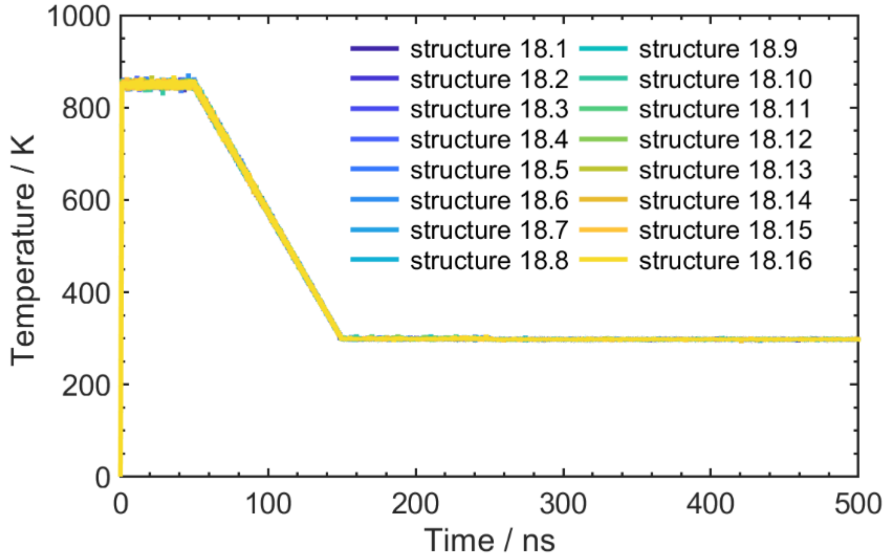

Figure S59. Simulated temperature of MD model 18 at different times.

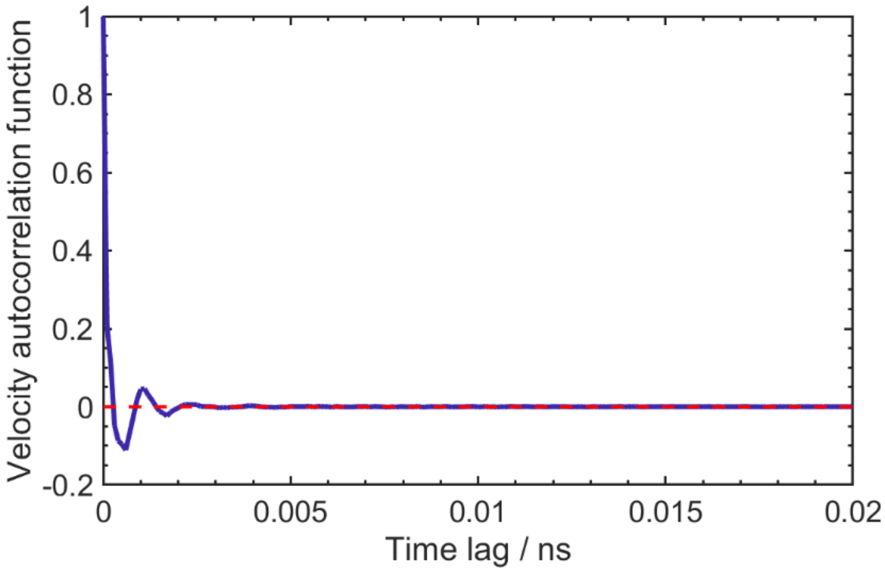

Figure S60. Velocity autocorrelation functions at different time lags of MD model 18.

2.19. MD model 19

100 Y6 and 3 PM6 with 20 repeating units (weight ratio of 2:1) were initially randomly placed in a cubic box using packmol[5]. Eight parallel models were constructed, denoted as structures 19.1-19.8. NPT simulation was performed to bring the system to 850 K over 1 ns and kept at this temperature for another 49 ns. The system was then brought to 298 K over 100 ns and kept at this temperature for another 100 ns. NVT simulation was then performed for 250 ns, and the last 200 ns was used for further processing to generate the dataset.

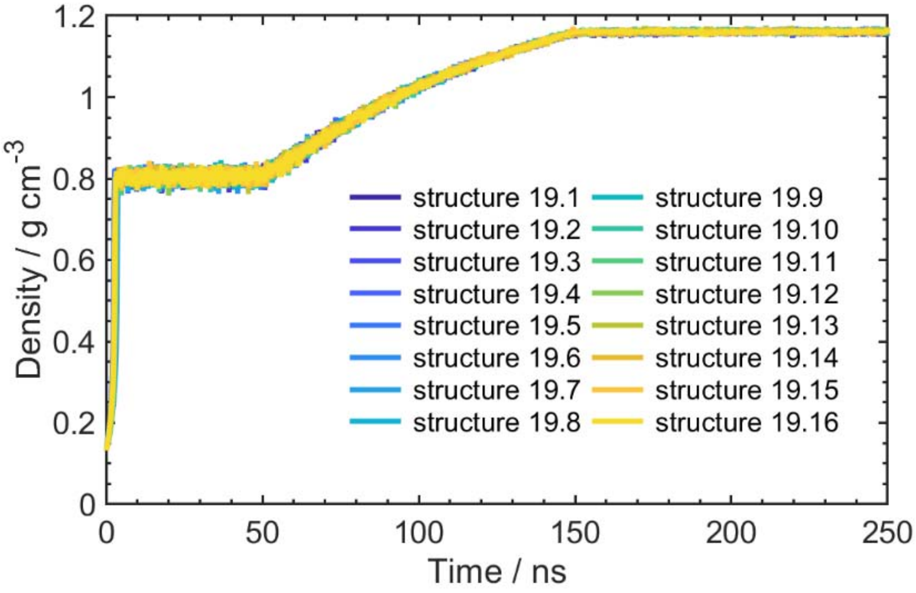

Figure S61. Simulated density of MD model 19 at different times.

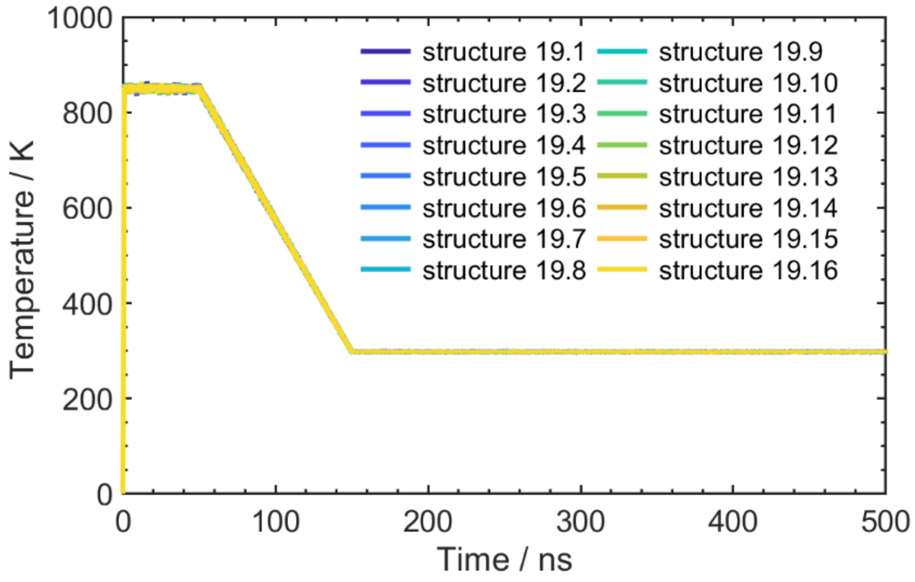

Figure S62. Simulated temperature of MD model 19 at different times.

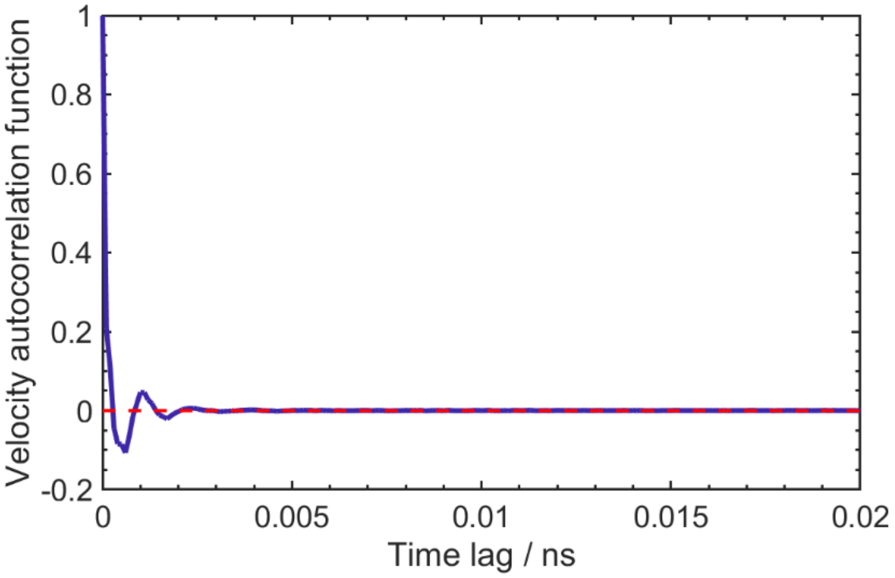

Figure S63. Velocity autocorrelation functions at different time lags of MD model 19.

2.20. MD model 20

100 Y6 and 6 PM6 with 20 repeating units (weight ratio of 1:1) were initially randomly placed in a cubic box. Eight parallel models were constructed, denoted as structures 20.1-20.8. NPT simulation was performed to bring the system to 850 K over 1 ns and kept at this temperature for another 49 ns. The system was then brought to 298 K over 100 ns and kept at this temperature for another 100 ns. NVT simulation was then performed for 250 ns, and the last 200 ns was used for further processing to generate the dataset.

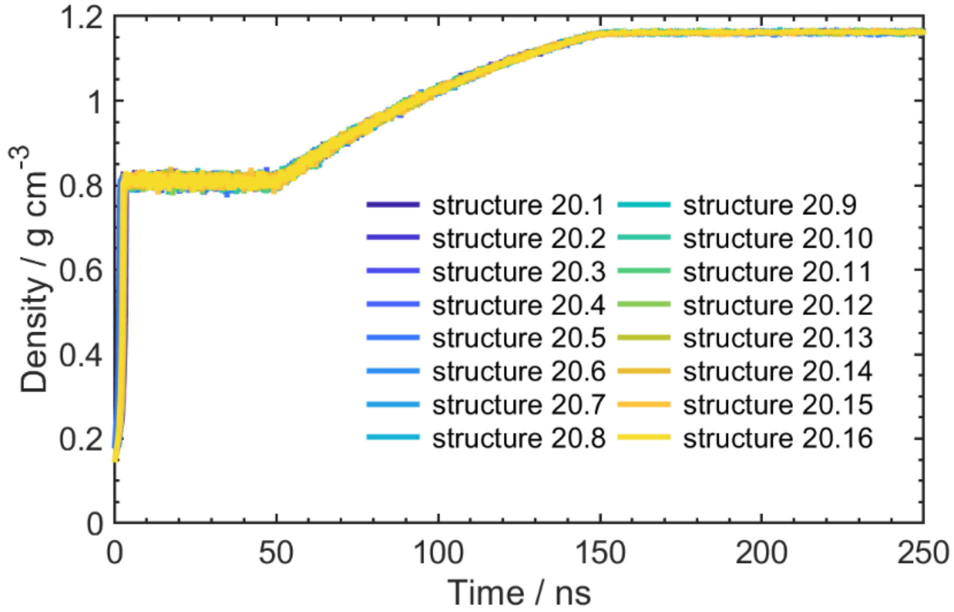

Figure S64. Simulated density of MD model 20 at different times.

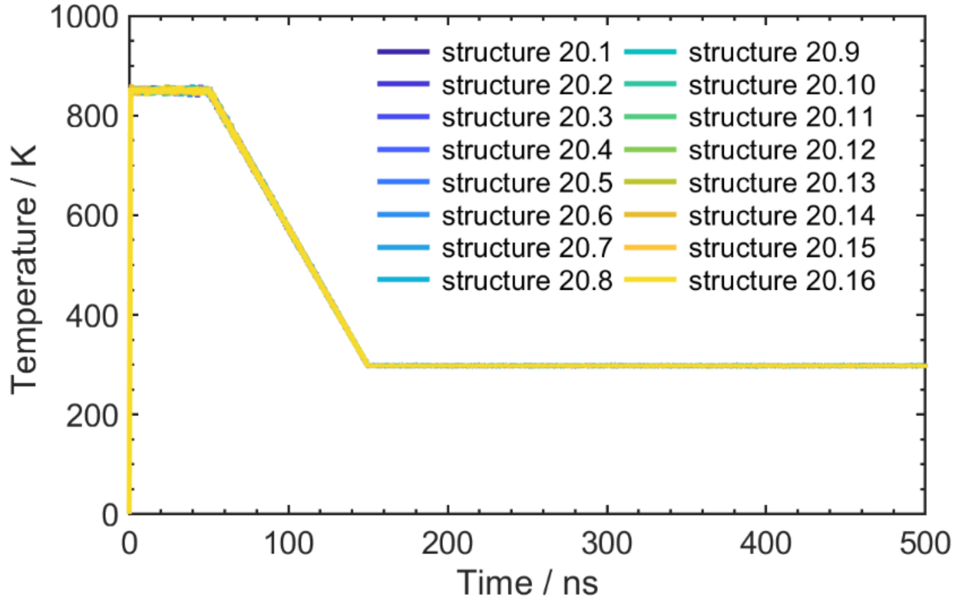

Figure S65. Simulated temperature of MD model 20 at different times.

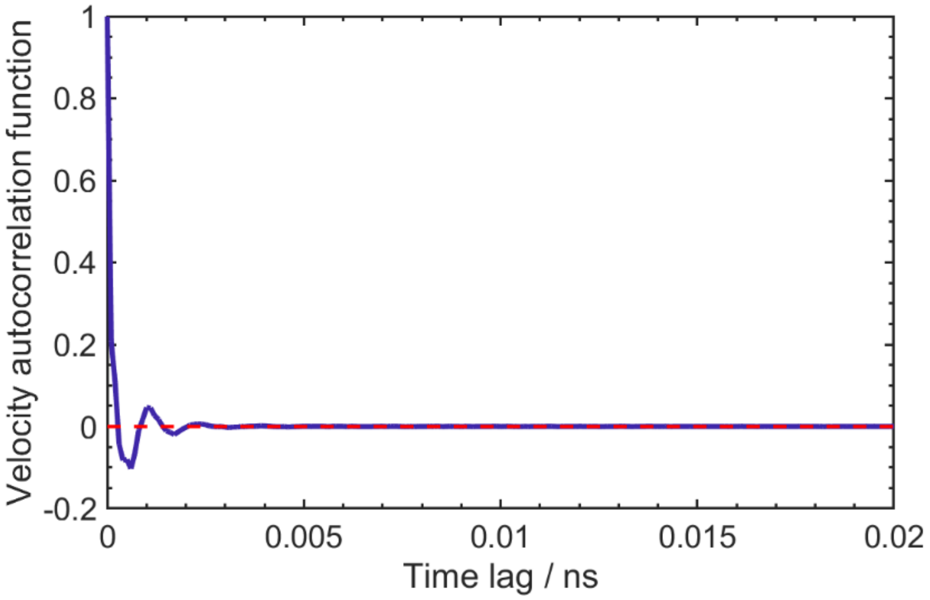

Figure S66. Velocity autocorrelation functions at different time lags of MD model 20.

2.21. MD model 21

100 Y6 and 12 PM6 with 20 repeating units (weight ratio of 1:2) were initially randomly placed in a cubic box. Eight parallel models were constructed, denoted as structures 21.1-21.8. NPT simulation was performed to bring the system to 850 K over 1 ns and kept at this temperature for another 49 ns. The system was then brought to 298 K over 100 ns and kept at this temperature for another 100 ns. NVT simulation was then performed for 250 ns, and the last 200 ns was used for further processing to generate the dataset.

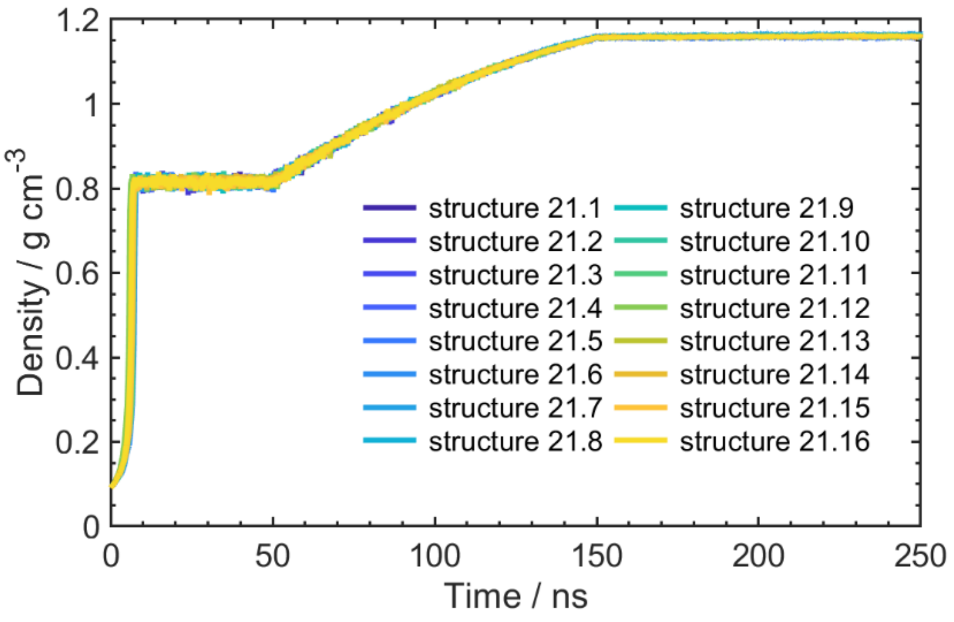

Figure S67. Simulated density of MD model 21 at different times.

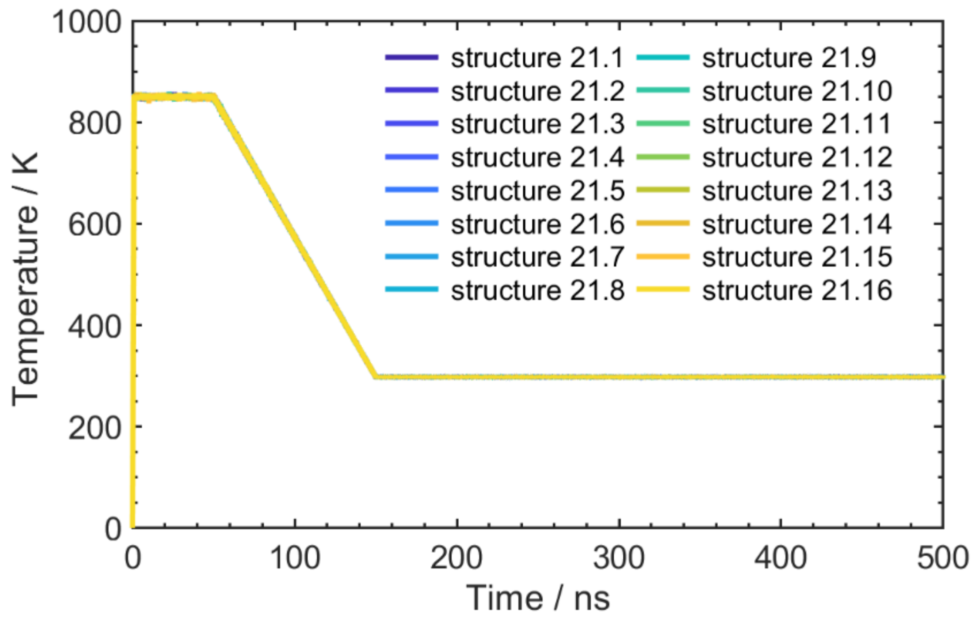

Figure S68. Simulated temperature of MD model 21 at different times.

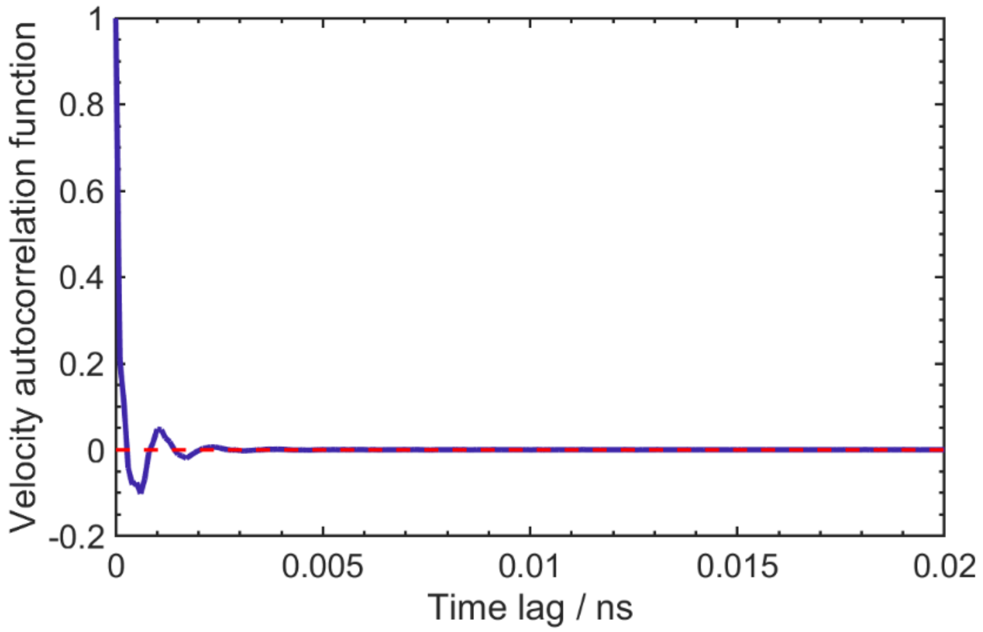

Figure S69. Velocity autocorrelation functions at different time lags of MD model 21.

2.22. MD model 22

100 Y6 and 24 PM6 with 20 repeating units (weight ratio of 1:2) were initially randomly placed in a cubic box. Eight parallel models were constructed, denoted as structures 21.1-21.8. NPT simulation was performed to bring the system to 850 K over 1 ns and kept at this temperature for another 49 ns. The system was then brought to 298 K over 100 ns and kept at this temperature for another 100 ns. NVT simulation was then performed for 250 ns, and the last 200 ns was used for further processing to generate the dataset.

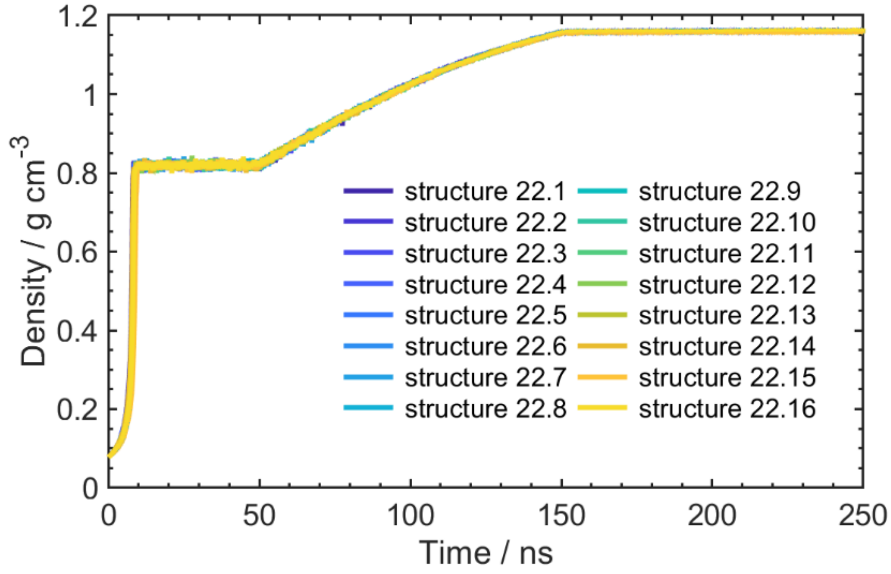

Figure S70. Simulated density of MD model 22 at different times.

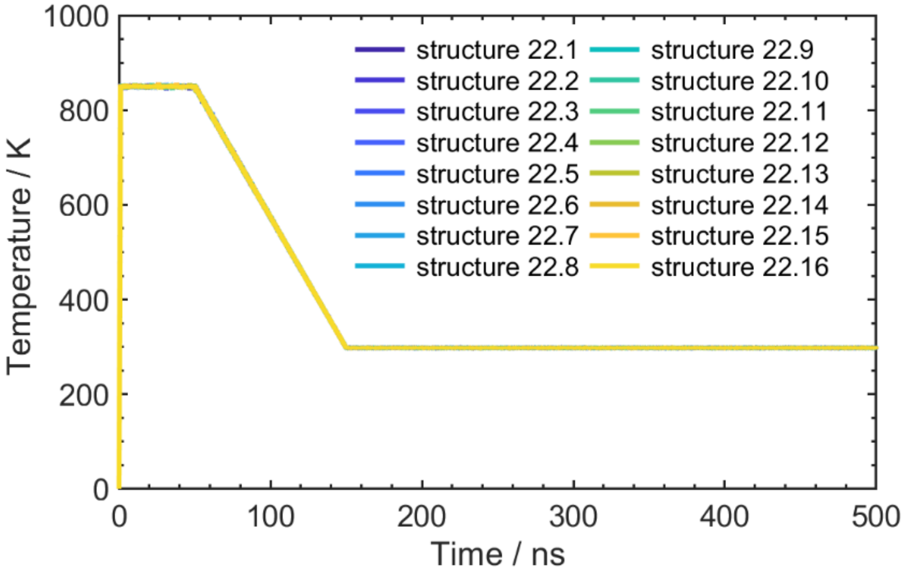

Figure S71. Simulated temperature of MD model 22 at different times.

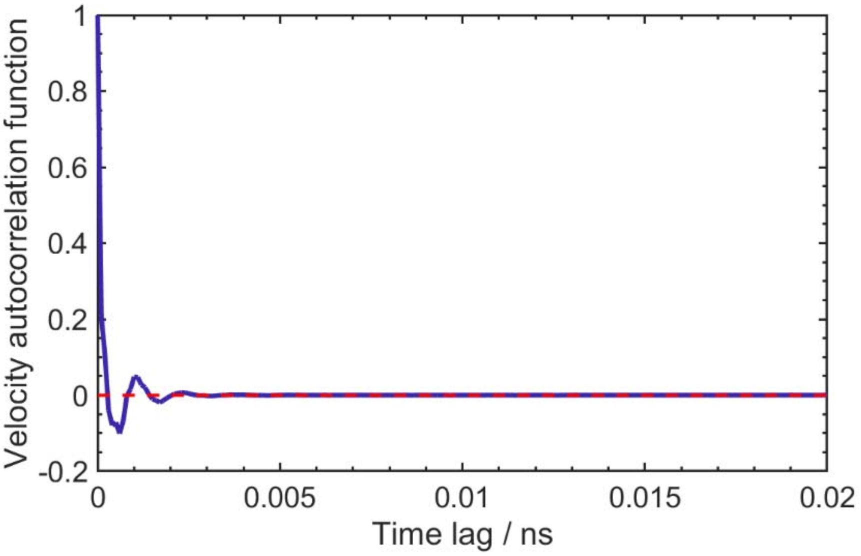

Figure S72. Velocity autocorrelation functions at different time lags of MD model 22.

3. Data processing

Figure S73 shows the data processing flow. First, the relative coordinates of surrounding atoms with respect to each Li^+ ion are extracted from the MD trajectories. Next, equivalent atom groups are generated by the definitions in **Table S1**. These groups are then sorted by their distance to the central atom (Li^+ or carbon atom in CO_2). Finally, a distance matrix was constructed, forming the final dataset. **Table S2** shows the size of the datasets for different MD models.

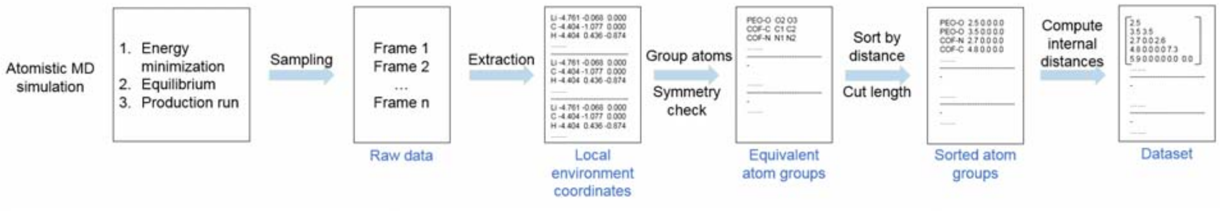

Figure S73. Illustration of the data processing flow.

Table S1. The atom sets used for processing the MD data in different tasks

Task	Atom group name	Molecule/Ion	Atoms	Number of groups used
ζ_A	CLO-O	ClO^-	O1, O2, O3, O4	6
	CLO-Cl	ClO^-	Cl	6
ζ_B	LINK-O1	COF-PEO-3	O3/O4[a]	2
	LINK-C1	COF-PEO-3	C7/C8	2
	LINK-N1	COF-PEO-3	N1/N2	2
	LINK-N4	COF-PEO-3	N3/N4	2
ζ_C	CONN-O1	COF-PEO-3	O1/O2[a]	2
	CONN-C1	COF-PEO-3	C6/C3	2
	CONN-C2	COF-PEO-3	C5/C2	2
	CONN-C3	COF-PEO-3	C7/C8	2
	CONN-O3	COF-PEO-3	O3/O4	2
ζ_D	PEO-C1	PEO	C10/C12[a]	2
	PEO-O1	PEO	O5/O6	2
	PEO-C2	PEO	C13/C15	2
	PEO-C3	PEO	C14/C16	2
	PEO-O2	PEO	O7/O8	2
	PEO-C4	PEO	C17/C19	2
	PEO-C5	PEO	C18/C20	2
	PEO-O3	PEO	O9/O10	2
	PEO-C6	PEO	C21/C22	2
ζ_E	CO2-C	CO_2	C	13
ζ_F	ZnO3-O	ZnO_3 core	O1, O2, O3	4
	ZnO3-Zn	ZnO_3 core	Zn	4
ζ_G	Y6-C1	Y6	C1/C2/C3/C5/C6/C8	2
	Y6-E1	Y6	C60/C62/C70/C74/C73/C67	2
	Y6-E2	Y6	C47/C49/C54/C57/C63/C64	2

[a]As the linkage and side chain-connections both have symmetry, only half of the atoms were used, depending on which half is closest to the central Li^+ or carbon atom in CO_2. See **Figures S74-S76** for the atom names.

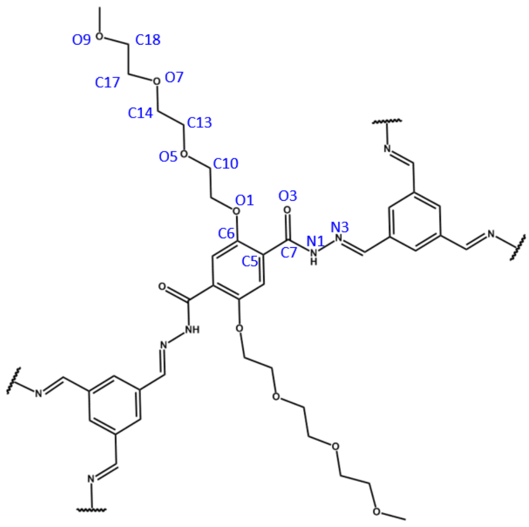

Figure S74. Illustration of the atom names in COF-PEO-3.

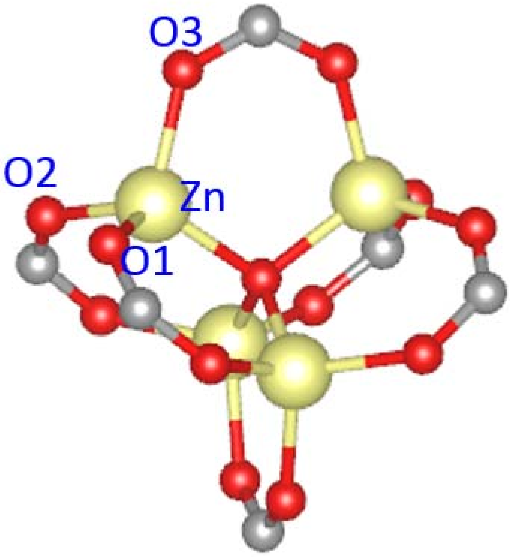

Figure S75. Illustration of the atom names in MOF-5.

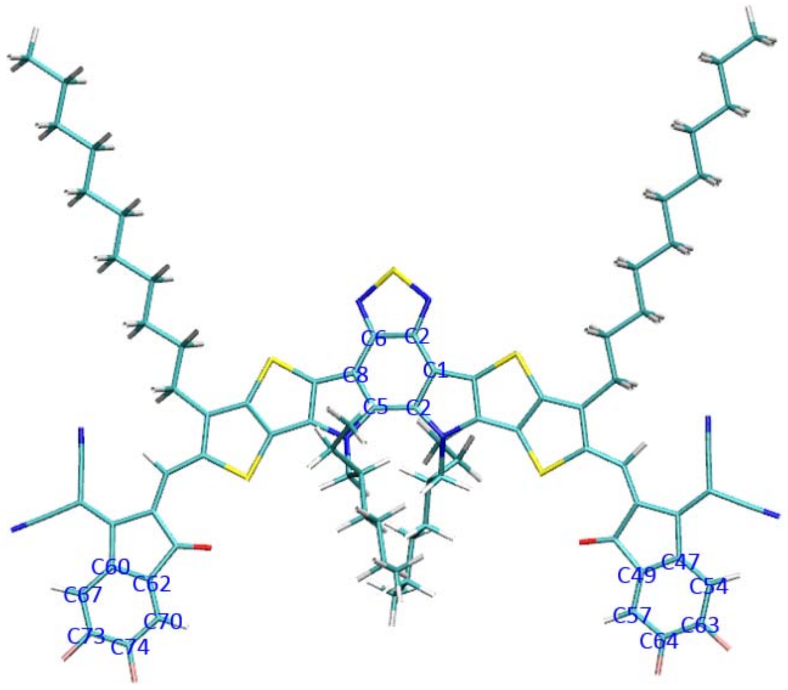

Figure S76. Illustration of the atom names in Y6.

Table S2. Size of the dataset for different MD models used for model training, validation and test.

MD model	Size of dataset
1	960,000
2	2,000,000
3	2,000,000
4	2,400,000
5	1,200,000
6	9,600,000
7	1,200,000
8	14,400,000
9	1,000,000
10	1,000,000
11	1,000,000
12	1,000,000
13	250,000
14	990,000
15	4,930,000
16	9,850,000
17	179,200
18	1,600,000
19	1,600,000
20	1,600,000
21	1,600,000

4. Model training

Unless othersise mentioned, the 80% of the dataset is used for training, 10% is used for validation, and 10% is used for test.

4.1. Li-ion transport in COF-PEO-3

Figure S77 shows the model for Li-ion transport in COF-PEO-3. The order of datasets fed into the model is designed to gradually increase their complexity based on our knowledge of these systems. For example, in task ζ_A, the first dataset (MD model 1) has the most ordered Li^+-$ClO4^-$ configurations; the second dataset (MD model 2) introduces disordered but tightly-packed configurations; the third dataset (MD model 3) introduces asymmetric configurations at the cluster surface; the fourth dataset (MD model 7) introduces loosely bound configurations; the fifth dataset (MD model 4) introduces new configurations close to the COF framework; the sixth dataset (MD model 8) introduces new configurations close to the COF framework with interacting side chains. This choice of order leads to well-differentiated labels in the target system. In tasks ζ_B and ζ_C, the first dataset (MD model 5) corresponds to a low salt concentration so that the configurations represent those close to the COF framework; the second dataset (MD model 6) increases the salt

concentration and leads to configurations towards the pore center; the third dataset (MD model 8) from the target system will capture missed configurations. In task ζ_D, the first dataset (MD model 7) captures configurations in a simpler system, while the second dataset (MD model 8) captures the additional configurations in the target system. The *k* values were tested and determined in each step to ensure accuracy, as shown in the following sections.

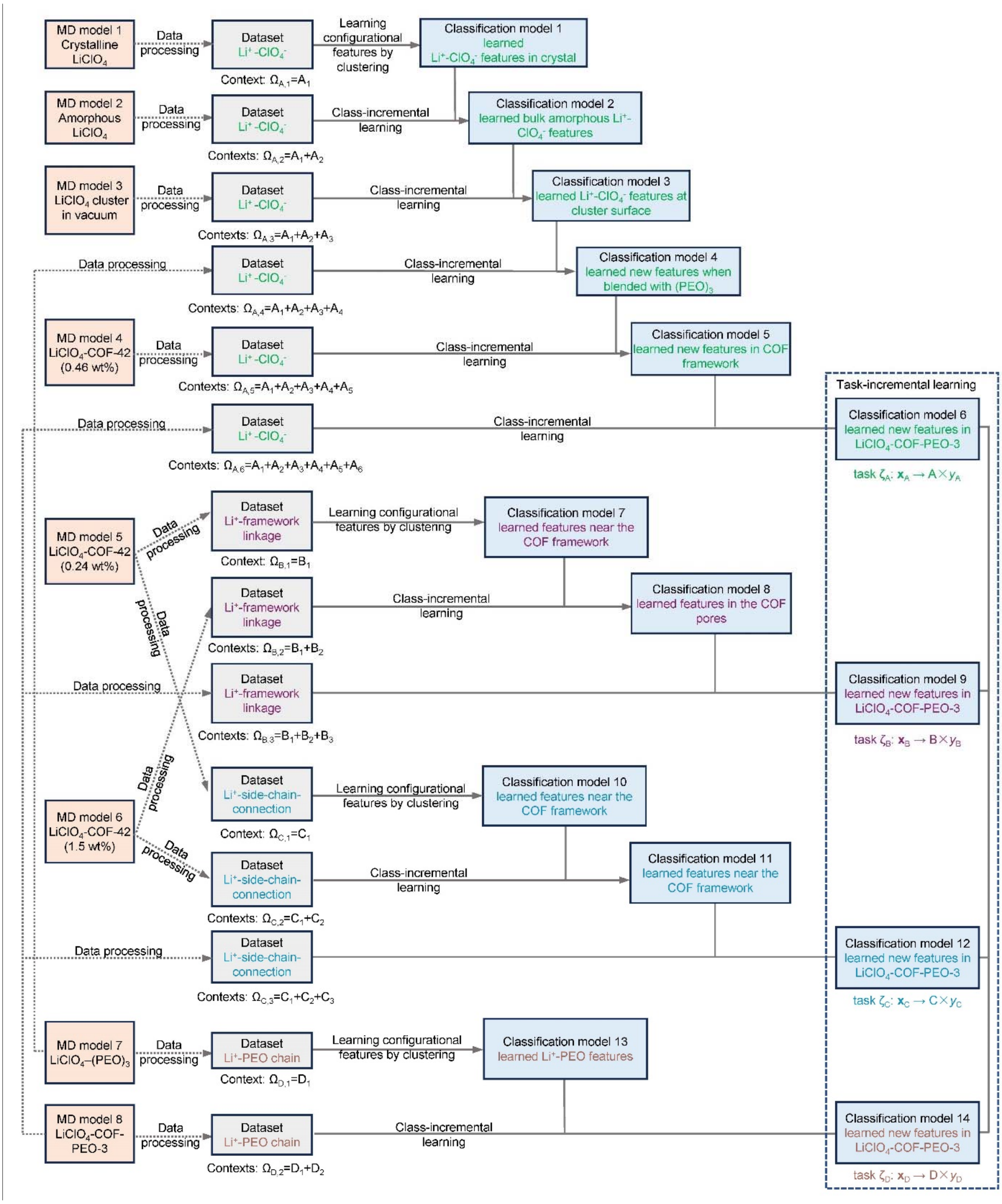

Figure S77. Illustration of applying the HiDiscover protocol to study Li-ion transport in COF-PEO-3 blended with $LiClO_4$. Refer to **Table 1** for definitions of tasks ζ_A, ζ_B, ζ_C, and ζ_D. A, B, C, and D denote contexts in tasks ζ_A, ζ_B, ζ_C, and ζ_D, respectively. $\mathbf{x}_\mathcal{C}$ is a vector representing atomic coordinates, and $y_\mathcal{C}$ denotes a feature in context $\mathcal{C}$. Ω represents a subset of the context set $\{\mathcal{C}\}$.

4.1.1. Task ζ_A

Based on tests in **Figures S78**-**S83**, k_1, k_2, k_3, k_4, k_5 and k_6 are determined to be 20, 20, 20, 30, 50, and 10, respectively.

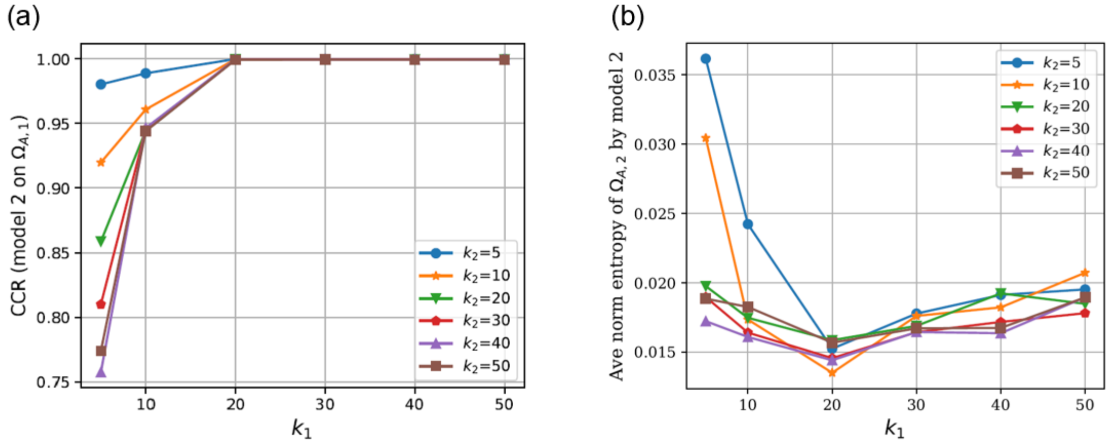

Figure S78. (a) Correct classification rate (CCR) of model 2 on dataset $\Omega_{A,1}$ and (b) average normalized entropy of $\Omega_{A,2}$ at different cluster numbers k_1.

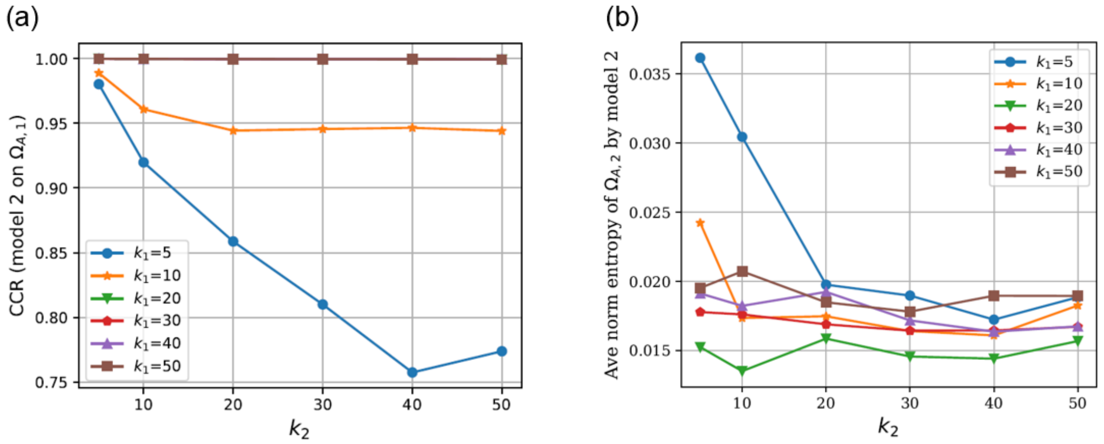

Figure S79. (a) Correct classification rate (CCR) of model 2 on $\Omega_{A,1}$ and (b) average normalized entropy of $\Omega_{A,2}$ at different cluster numbers k_2. We note that the data for k_1=20,30,40,50 overlap in (a).

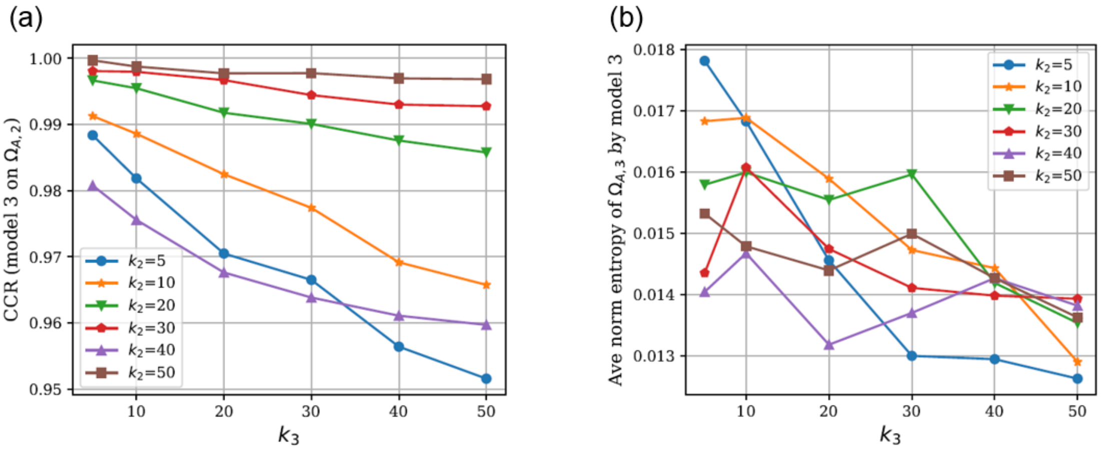

Figure S80. (a) Correct classification rate (CCR) of model 3 on dataset $\Omega_{A,2}$ and (b) average normalized entropy of dataset $\Omega_{A,3}$ at different cluster numbers k_3. Previous determined parameter: $k_1 = 20$.

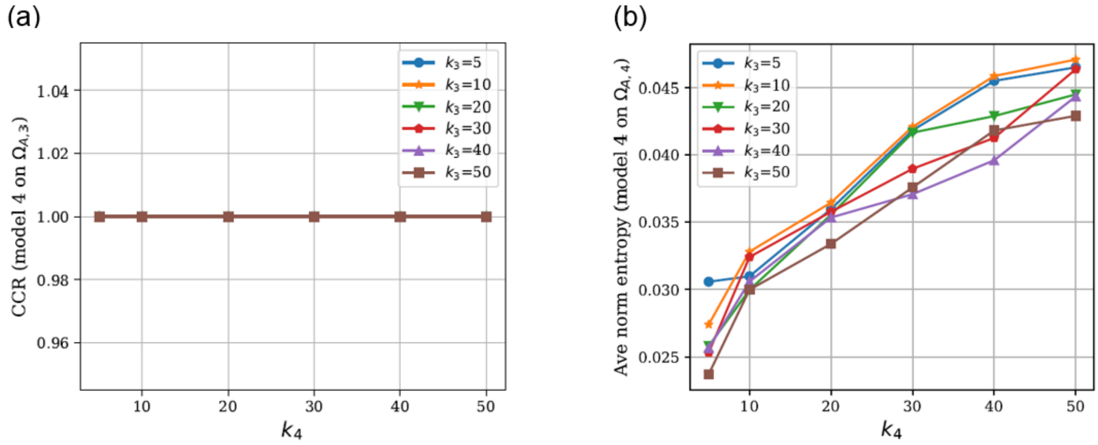

Figure S81. (a) Correct classification rate (CCR) of model 4 on dataset $\Omega_{A,3}$ and (b) average normalized entropy of dataset $\Omega_{A,4}$ at different cluster numbers k_4. Previous determined parameters: k_1=20; k_2=20.

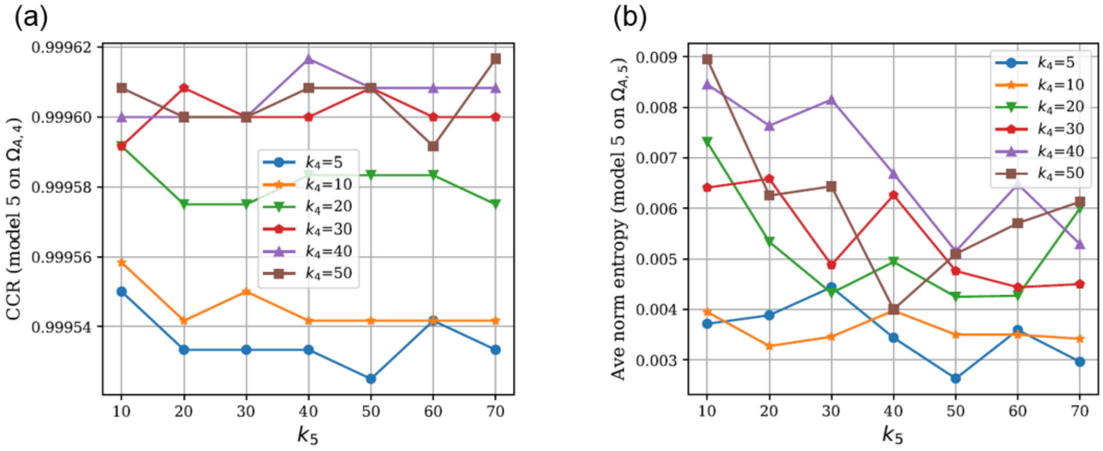

Figure S82. (a) Correct classification rate (CCR) of model 5 on dataset $\Omega_{A,4}$ and (b) average normalized entropy of dataset $\Omega_{A,5}$ at different cluster numbers k_5. Previous determined parameters: k_1=20; k_2=20; k_3=20.

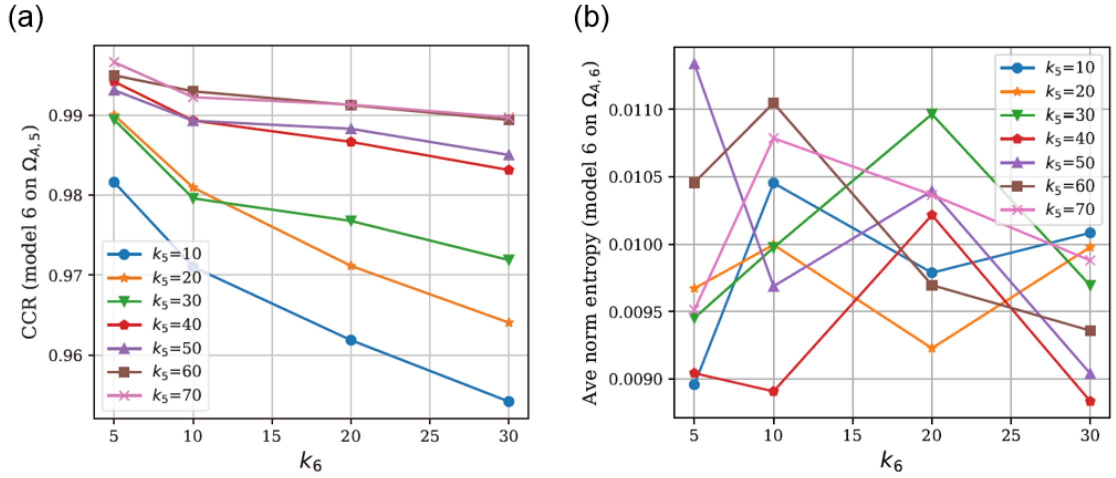

Figure S83. (a) Correct classification rate (CCR) of model 6 on dataset $\Omega_{A,5}$ and (b) average normalized entropy of dataset $\Omega_{A,6}$ at different cluster numbers k_6. Previous determined parameters: k_1=20; k_2=20; k_3=20; k_4=30.

4.1.2. Task ζ_B

By tests in **Figures S84-S86**, k_7, k_8, and k_9 are determined to be 30, 10, and 10, respectively.

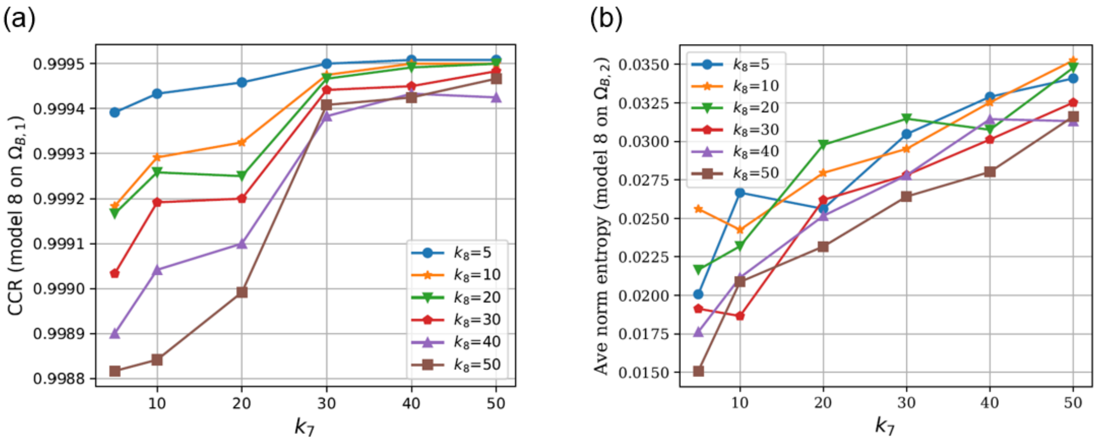

Figure S84. (a) Correct classification rate (CCR) of model 8 on dataset $\Omega_{B,1}$ and (b) average normalized entropy of dataset $\Omega_{B,2}$ at different cluster numbers k_7.

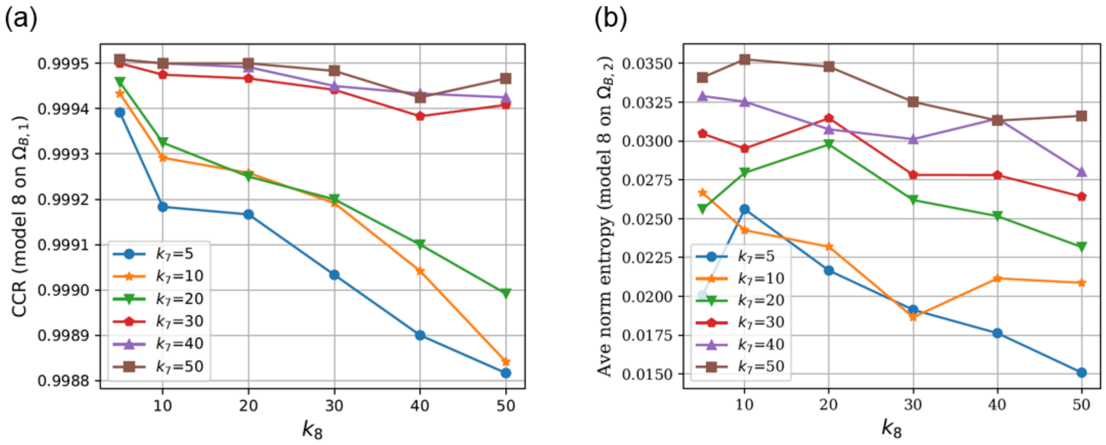

Figure S85. (a) Correct classification rate (CCR) of model 8 on dataset $\Omega_{B,1}$ and (b) average normalized entropy of dataset $\Omega_{B,2}$ at different cluster numbers k_8.

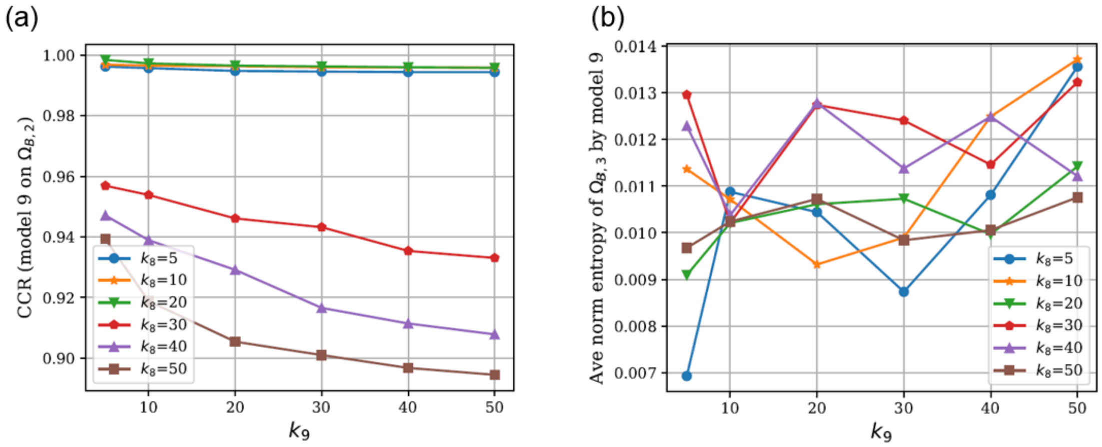

Figure S86. (a) Correct classification rate (CCR) of model 9 on dataset $\Omega_{B,2}$ and (b) average normalized entropy of dataset $\Omega_{B,3}$ at different cluster numbers k_9. Previously determined parameter: k_7=30.

4.1.3. Task ζ_C

By tests in **Figures S87-S89**, k_{10}, k_{11}, and k_{12} are determined to be 30, 10, and 10, respectively.

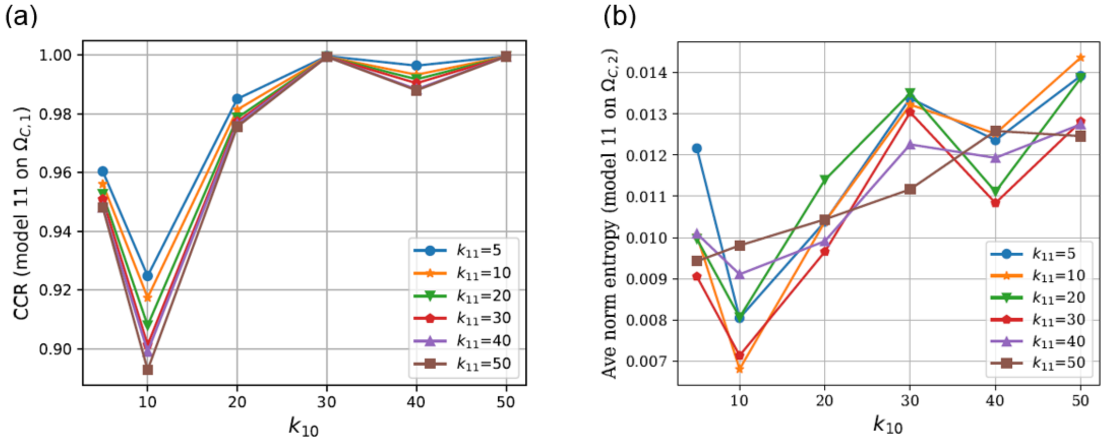

Figure S87. (a) Correct classification rate (CCR) of model 11 on dataset $\Omega_{C,1}$ and (b) average normalized entropy of dataset $\Omega_{C,2}$ at different cluster numbers k_{10}.

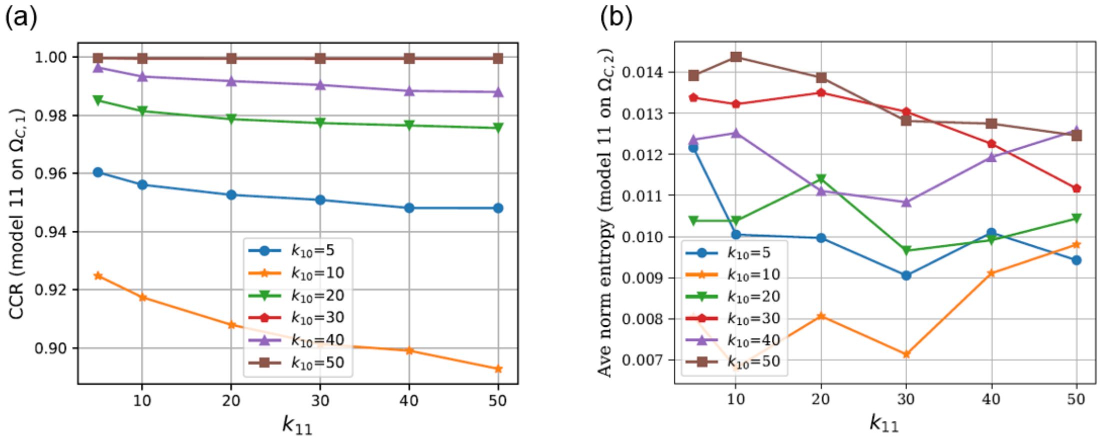

Figure S88. (a) Correct classification rate (CCR) of model 11 on dataset $\Omega_{C,1}$ and (b) average normalized entropy of dataset $\Omega_{C,2}$ at different cluster numbers k_{11}. We note that the data of k_{10}=30,50 overlap in (a).

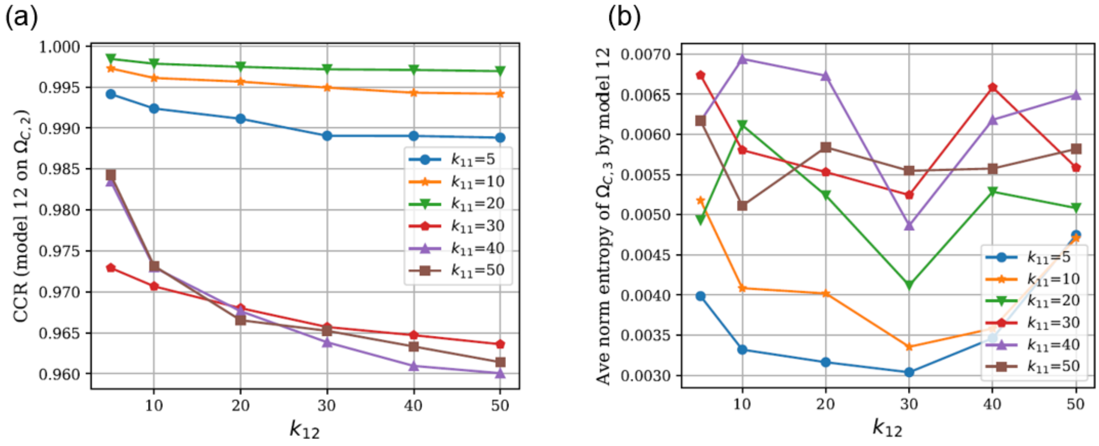

Figure S89. (a) Correct classification rate (CCR) of model 12 on dataset $\Omega_{C,2}$ and (b) average normalized entropy of dataset $\Omega_{C,3}$ at different cluster numbers k_{12}. Previously determined parameter: k_{10}=30.

4.1.4. Task ζ_D

By tests in **Figures S90-S91**, k_{13} and k_{14} are determined to be 20 and 30, respectively.

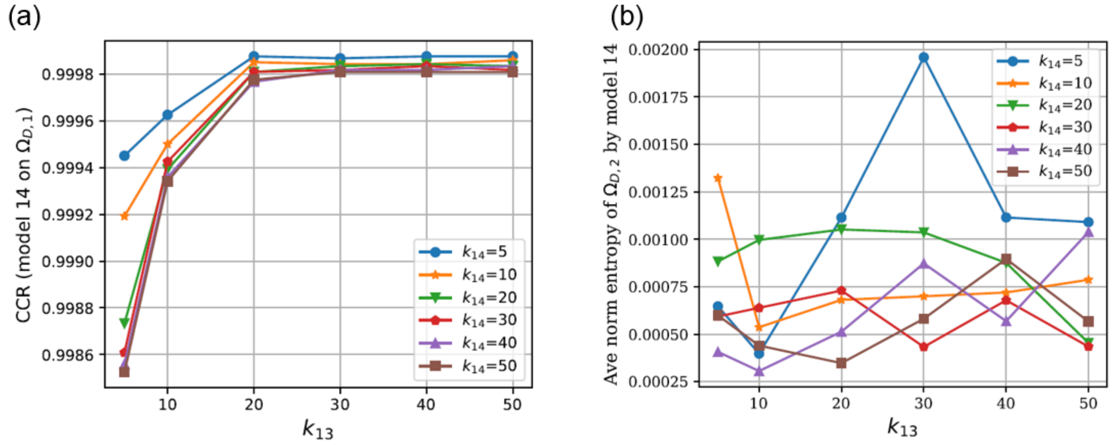

Figure S90. (a) Correct classification rate (CCR) of model 14 on dataset $\Omega_{D,1}$ and (b) average normalized entropy of dataset $\Omega_{D,2}$ at different cluster numbers k_{13}.

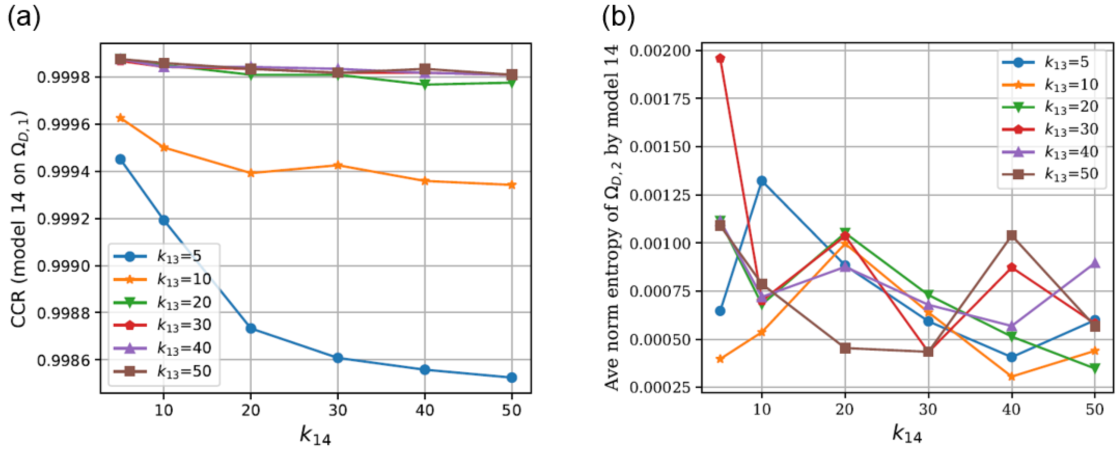

Figure S91. (a) Correct classification rate (CCR) of model 14 on dataset $\Omega_{D,1}$ and (b) average normalized entropy of dataset $\Omega_{D,2}$ at different cluster numbers k_{14}.

4.1.5. Accuracies on the test set

Accuracies of >99% were achieved on the test set, as shown in **Figure S92**.

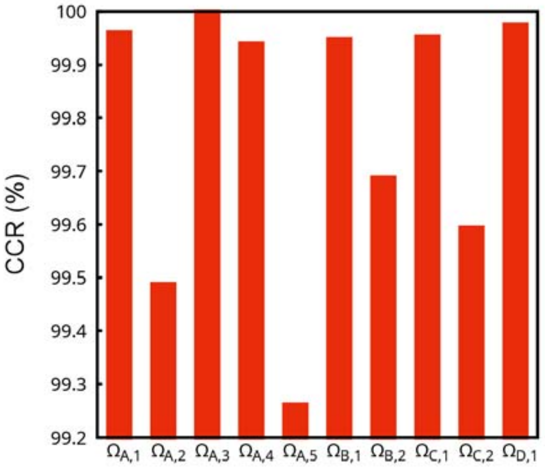

Figure S92. Correct classification rate (CCR) of the combined model on the test sets ($\Omega_{A,1}$, $\Omega_{A,2}$, $\Omega_{A,3}$, $\Omega_{A,4}$, $\Omega_{A,5}$, $\Omega_{B,1}$, $\Omega_{B,2}$, $\Omega_{C,1}$, $\Omega_{C,2}$, and $\Omega_{D,1}$) for Li-ion transport in covalent organic framework COF-PEO-3.

4.2. CO_2 adsorption in MOF-5

Figure S93 shows the model for CO_2 adsorption in MOF-5. We briefly discuss the order of datasets in tasks ζ_E and ζ_F. In task ζ_E, the complexities of the four datasets are not immediately seen. In this case, we found that very high CCRs can be obtained at different orders of the four datasets, suggesting that the contexts of these datasets have minimal overlap. Here, we used the order from high CO_2 density to low CO_2 density. In task ζ_F, we increase the concentration of CO_2 in MOF-5 to allow new configurations to gradually emerge. However, our test shows that the first dataset (MD model 13) covers most of the contexts in the following datasets. In this case, MD models 14 and 15 can still be used for incremental learning, although the new labels have minimal ratios; due to the highly overlapped context, the dataset from MD model 16 cannot be used for further incremental learning to generate a new cluster. The k values were tested and determined in each step to ensure accuracy, as shown in the following sections.

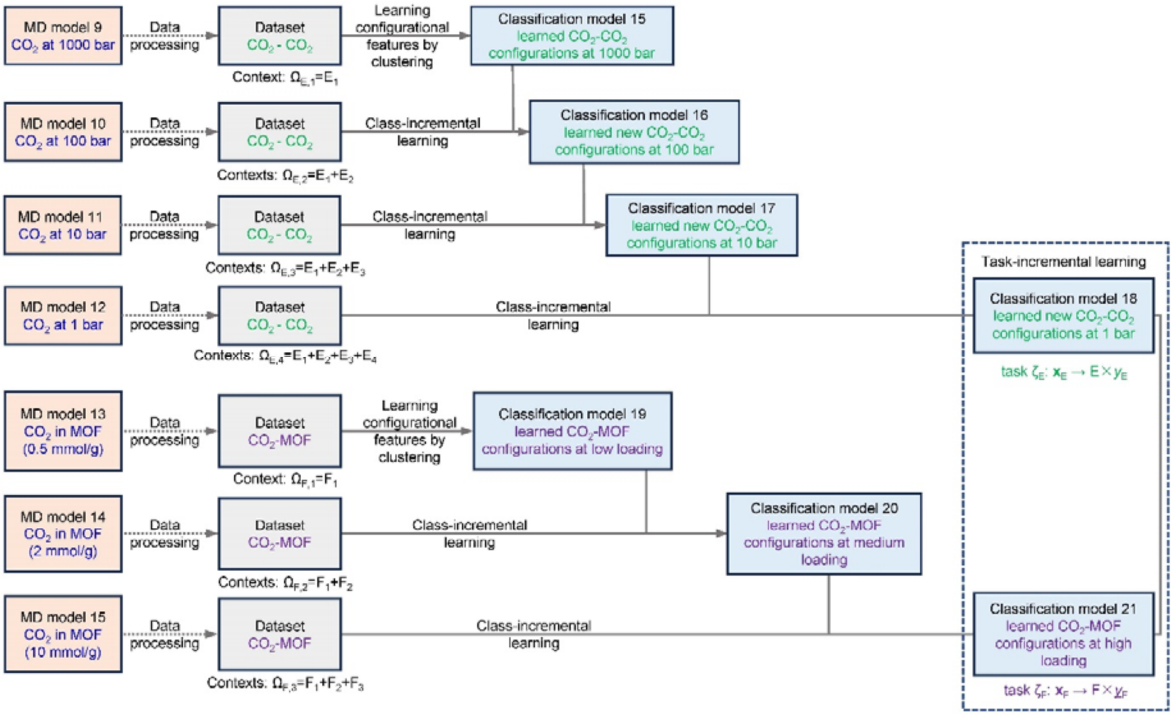

Figure S93. Illustration of applying the HiDiscover protocol in studying CO_2 adsorption in MOF-5. Tasks ζ_E and ζ_F correspond to CO_2-CO_2 and CO_2-MOF configurations, respectively. E and F denote contexts in tasks ζ_E and ζ_F, respectively. $\mathbf{x}_\mathcal{C}$ is a vector representing atomic coordinates, and $y_\mathcal{C}$ denotes a feature in context $\mathcal{C}$. Ω represents a subset of the context set $\{\mathcal{C}\}$.

4.2.1. Task ζ_E

By tests in **Figures S94-S97**, k_{15}, k_{16}, k_{17}, and k_{18} are determined to be 30, 30, 40, and 10, respectively.

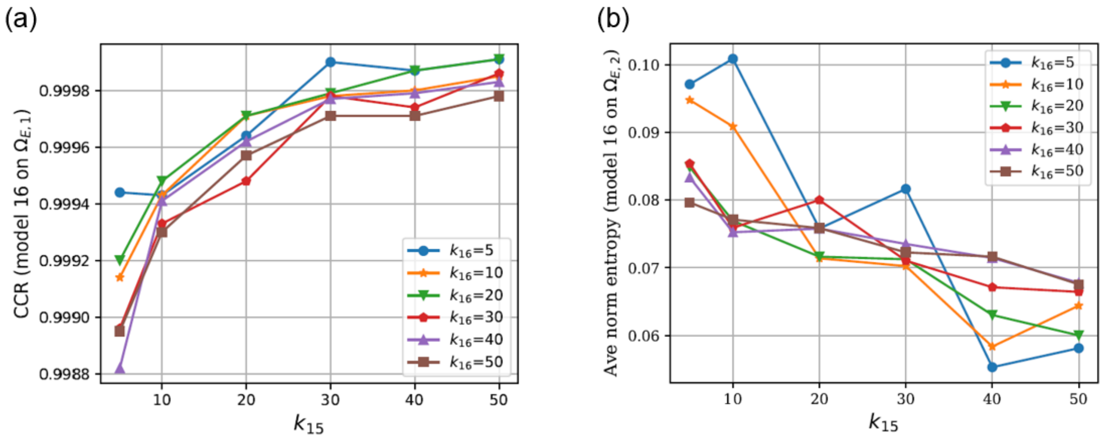

Figure S94. (a) Correct classification rate (CCR) of model 16 on dataset $\Omega_{E,1}$ and (b) average normalized entropy of dataset $\Omega_{E,2}$ at different cluster numbers k_{15}.

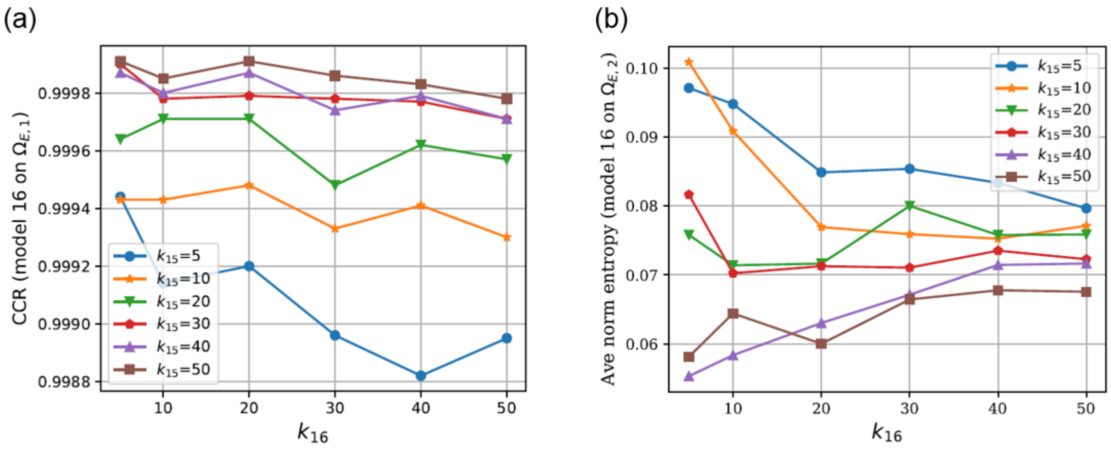

Figure S95. (a) Correct classification rate (CCR) of model 16 on dataset $\Omega_{E,1}$ and (b) average normalized entropy of dataset $\Omega_{E,2}$ at different cluster numbers k_{16}.

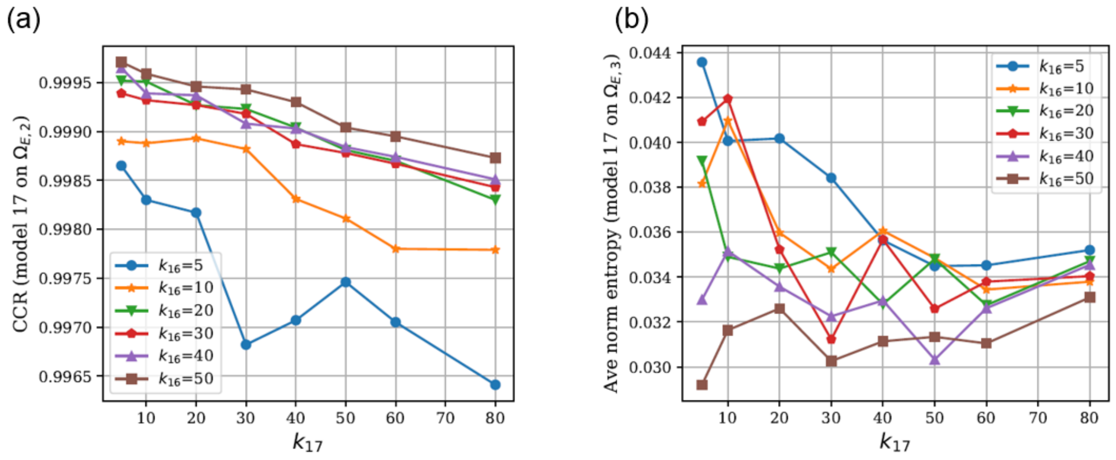

Figure S96. (a) Correct classification rate (CCR) of model 17 on dataset $\Omega_{E,2}$ and (b) average normalized entropy of dataset $\Omega_{E,3}$ at different cluster numbers k_{17}. Previously determined parameter: k_{15}=30.

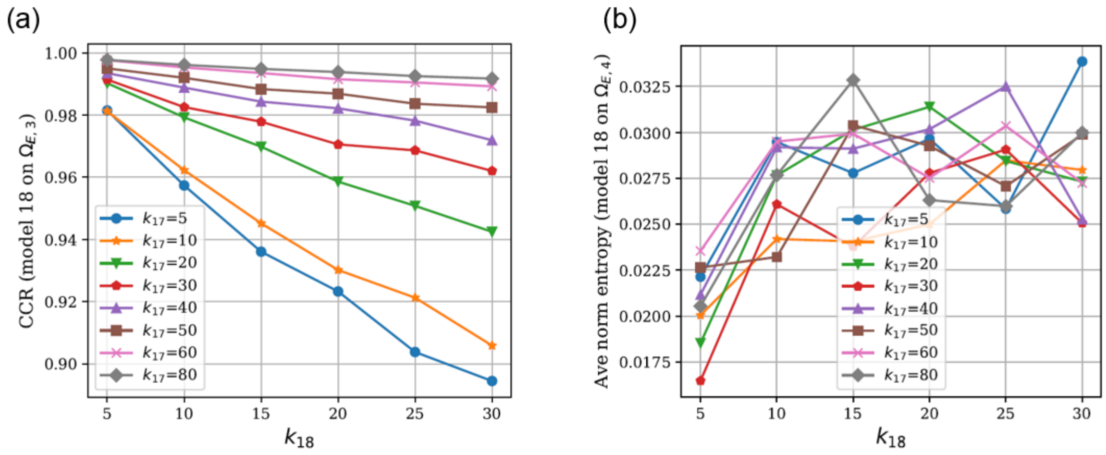

Figure S97. (a) Correct classification rate (CCR) of model 18 on dataset $\Omega_{E,3}$ and (b) average normalized entropy of dataset $\Omega_{E,4}$ at different cluster numbers k_{18}. Previously determined parameters: k_{15}=30; k_{16}=30.

4.2.2. Task ζ_F

By tests in **Figures S98-S100**, k_{19}, k_{20}, and k_{21} are determined to be 20, 10, and 2, respectively.

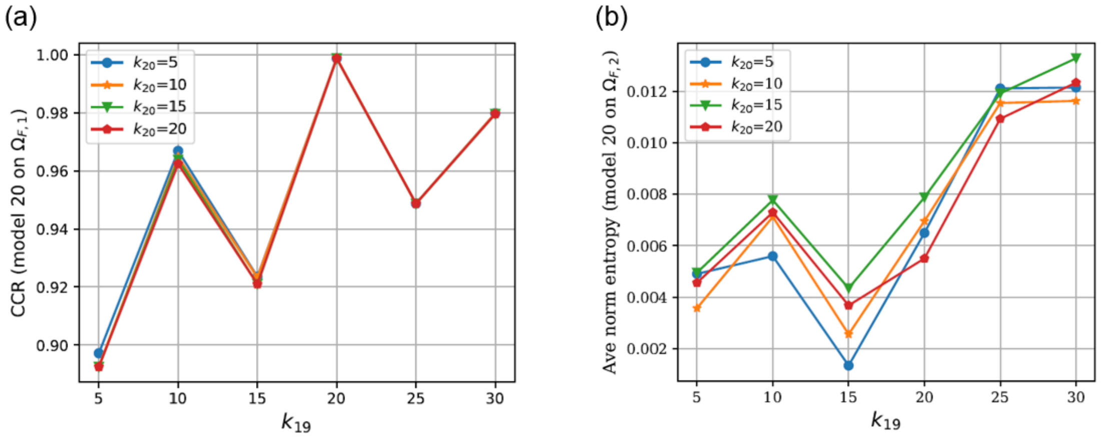

Figure S98. (a) Correct classification rate (CCR) of model 20 on dataset $\Omega_{F,1}$ and (b) average normalized entropy of dataset $\Omega_{F,2}$ at different cluster numbers k_{19}.

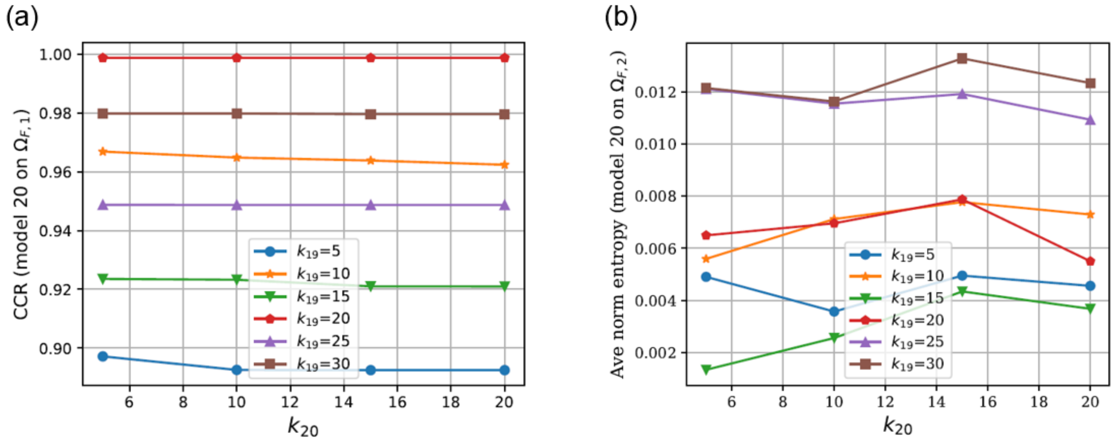

Figure S99. (a) Correct classification rate (CCR) of model 20 on dataset $\Omega_{F,1}$ and (b) average normalized entropy of dataset $\Omega_{F,2}$ at different cluster numbers k_{20}.

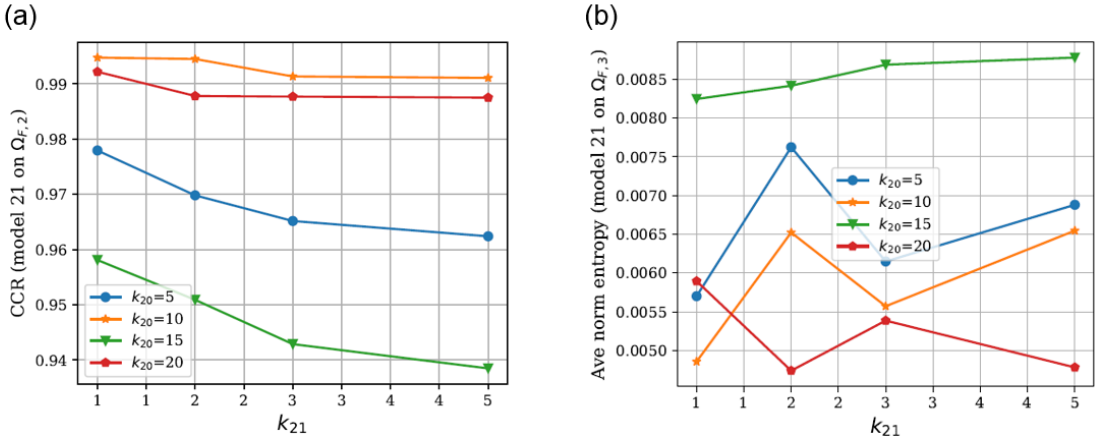

Figure S100. (a) Correct classification rate (CCR) of model 21 on dataset $\Omega_{F,2}$ and (b) average normalized entropy of dataset $\Omega_{F,3}$ at different cluster numbers k_{21}. Previously determined parameter: k_{19}=20.

4.2.3. Accuracies on the test set

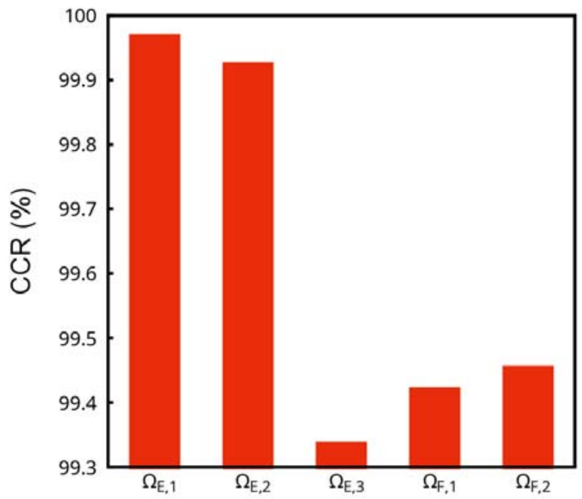

Figure S101. Correct classification rate (CCR) of the final model on the test sets ($\Omega_{E,1}$, $\Omega_{E,2}$, $\Omega_{E,3}$, $\Omega_{F,1}$, and $\Omega_{F,2}$) for CO_2 adsorption in MOF-5.

4.3. Molecular packing in the active layer of the PM6:Y6 organic solar cell

Figure S102 shows the model for identifying short-range Y6-Y6 contacts in PM6:Y6 blends. In this case, the dataset with the simplest packing (MD model 17) is the first to be fed into the model. The dataset for amorphous packing (MD model 18) is used subsequently to introduce disordered short-range contacts. We tried to use MD models 19-22 for further incremental learning; however,

no new cluster can be obtained, suggesting their highly overlapped contexts with MD model 18. The k values were tested and determined in each step to ensure accuracy, as shown in the following sections.

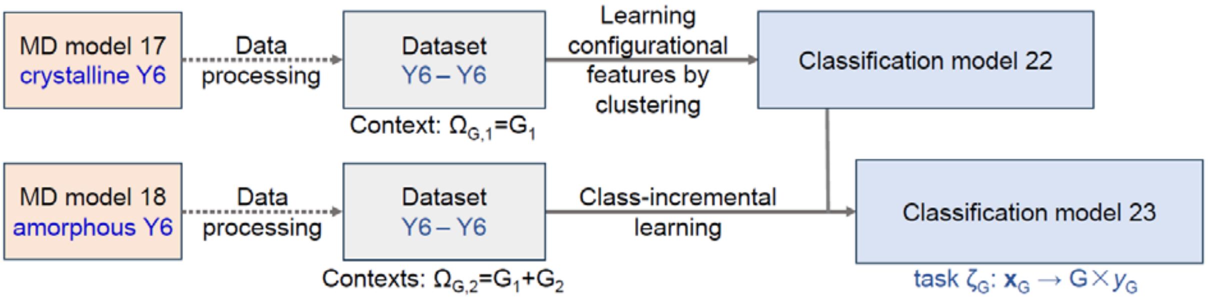

Figure S102. Illustration of applying the HiDiscover protocol in studying electron transport pathways in PM6:Y6 blends (task ζ_G). $\mathbf{x}_G$ is a vector representing atomic coordinates, and y_G denotes a feature in context G. Ω represents a subset of the context set {G}.

4.3.1. Task ζ_G

By tests in **Figures S103** and **S104**, k_{22} and k_{23} are determined to be 15 and 40, respectively. The CCR is calculated to be 99.92%.

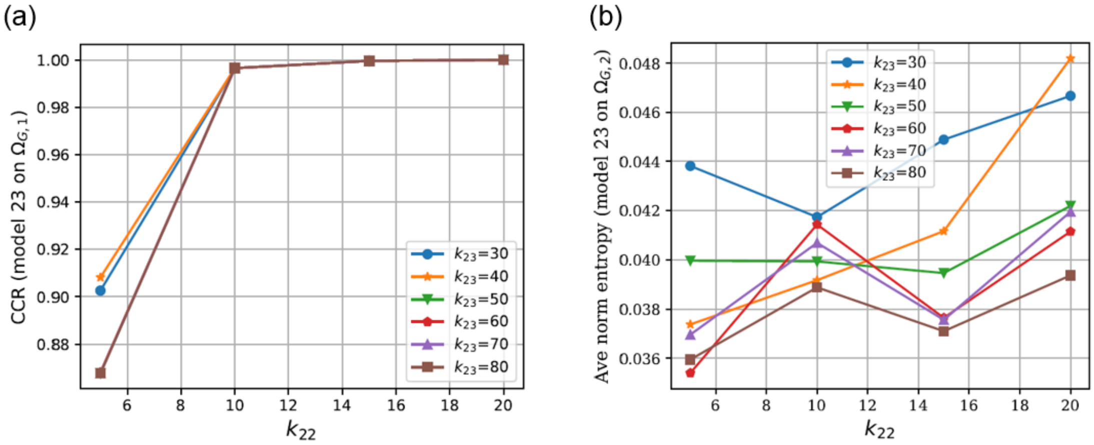

Figure S103. (a) Correct classification rate (CCR) of model 23 on dataset $\Omega_{G,1}$ and (b) average normalized entropy of dataset $\Omega_{G,2}$ at different cluster numbers k_{22}.

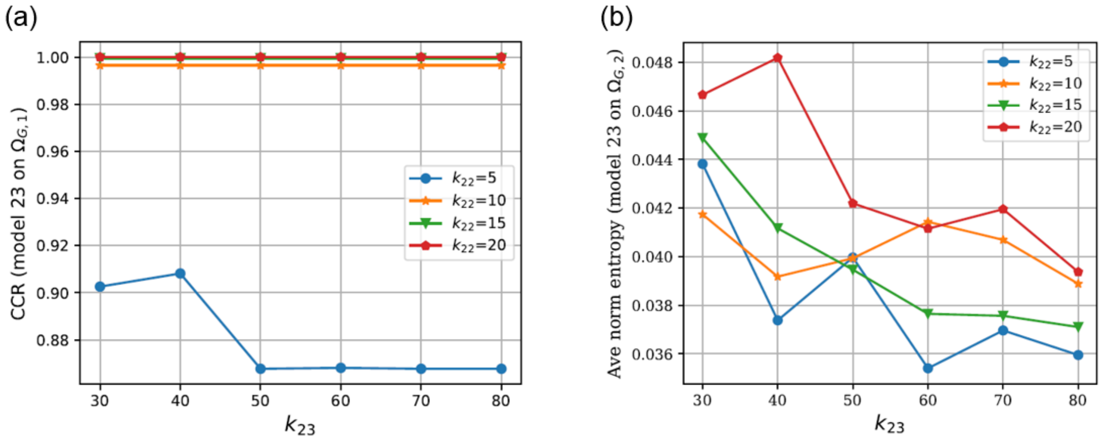

Figure S104. (a) Correct classification rate (CCR) of model 23 on dataset $\Omega_{G,1}$ and (b) average normalized entropy of dataset $\Omega_{G,2}$ at different cluster numbers k_{23}.

4.4. Impact of temporal correlation in datasets

Data generated from molecular dynamics simulations may have some degree of correlation, which may lead to inaccuracy in model training in some cases. However, considering the short time step (1 fs) in the molecular dynamics simulations and the long trajectories used in the analysis (10^8 – 10^9 fs), this degree of correlation is small as demonstrated in the velocity autocorrelation functions shown in **Section 1**. Overall, the motions are not correlated in the time intervals we used to generate the dataset (0.1 ns), except for MD model 12 (the gas state of CO_2 and has few particle collisions), for which system the correlation is 0.077 at 0.1-ns time lag and disappears at ~0.3-ns time lag. To further investigate the impact of temporal correlation in the dataset, we have sampled the trajectory of MD model 12 at larger time intervals (up to 10 ns). As can be seen in **Figures S105** and **S106**, for the range of time intervals (0.1-10 ns) considered, the accuracy of the trained model remains high and the predicted results are stable.

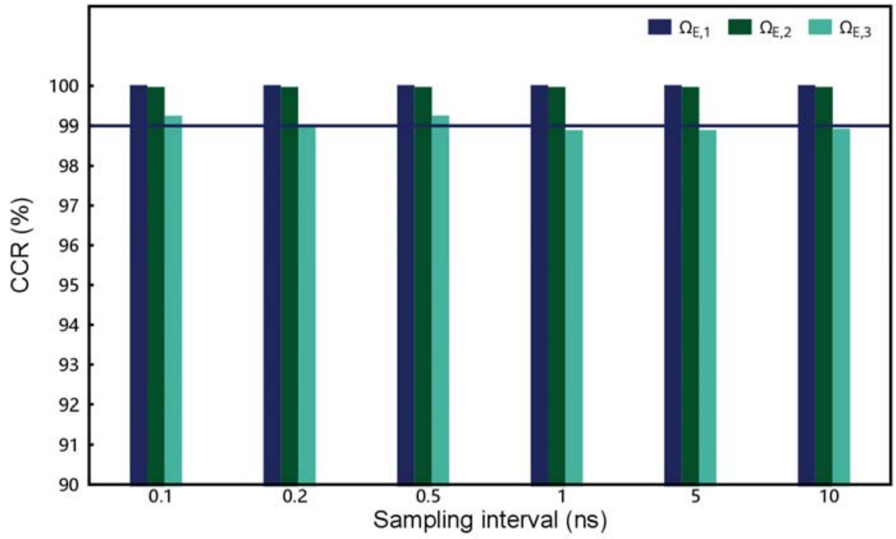

Figure S105. Correct classification rate (CCR) of the trained model on the test sets ($\Omega_{E,1}$, $\Omega_{E,2}$, and $\Omega_{E,3}$) in task ζ_E (refer to the main article for its definition) with MD model 12 sampled at different time intervals (longer times have smaller correlation). The solid line shows 99% accuracy.

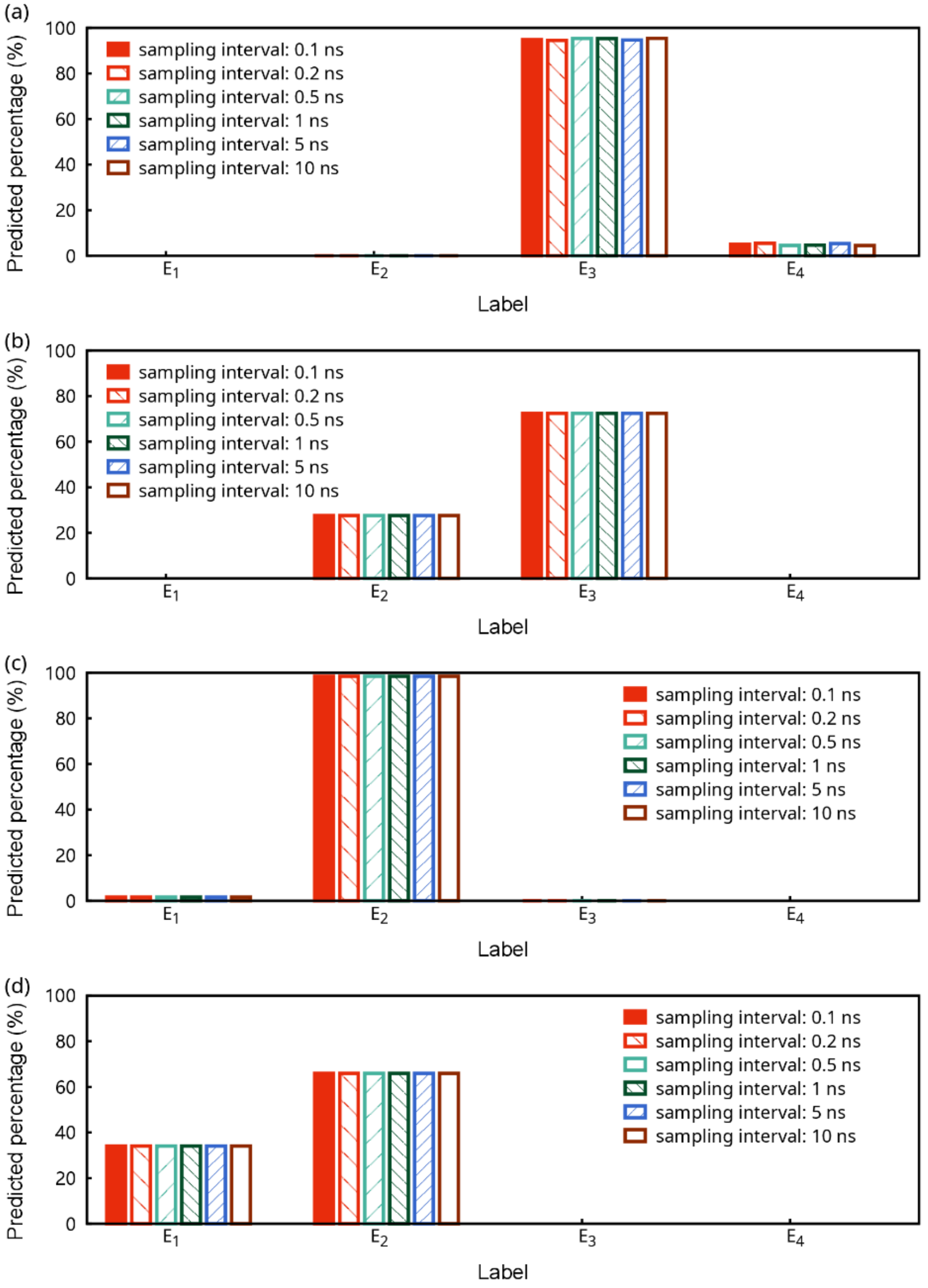

Figure S106. Ratios of predicted labels (among all labels, showing as percentages) on the target molecular dynamics (MD) models (a) 13, (b) 14, (c) 15, and (d) 16 in task ζ_E (refer to the main article for its definition), applied with models trained with MD model 12 sampled at different time intervals (longer times have smaller correlation). Refer to the main article for definitions of labels E_1, E_2, E_3, and E_4.

4.5. Computational costs and benchmarks of the trajectory lengths for the reference molecular models

In this study, the main part of the computational time was spent on the molecular dynamics simulations of the target and reference molecular systems (up to tens of thousands of CPU hours and thousands of GPU hours in this study), while that on model training and prediction (up to hundreds of CPU hours) was negligible in comparison. Each molecular dynamics simulation was run on 16 cores (AMD Epyc CPU) with an Nvidia A100/A800/3090/4090 GPU, depending on their availability on our computing cluster. The CPU and GPU hours for the production runs are summarized in **Table S3**. We counted the increased computational cost when studying the three examples using the HiDiscover protocol compared to the conventional MD study due to the introduced new molecular systems (the reference systems that are not covered by the target systems). It is estimated that the HiDiscover protocol increases the computational cost by 80% and 150% in the studies of Li-ion transport and CO_2 adsorption, respectively (**Figure S107**). On the other hand, the increased computational cost is negligible (0.00005%) in the case of Y6:PM6 heterojunctions. Overall, the simulation of Y6:PM6 heterojunctions cost the most computational resources among the three cases, amounting to ~35000 CPU hours and 2184 GPU hours, while that of CO_2 adsorption only took 400 CPU hours and 25 GPU hours. Clearly, the computational cost for mechanistic studies of multi-component materials and that introduced in the HiDiscover protocol vary in each problem. In general, including more new reference systems would lead to higher computational costs in the HiDiscover protocol.

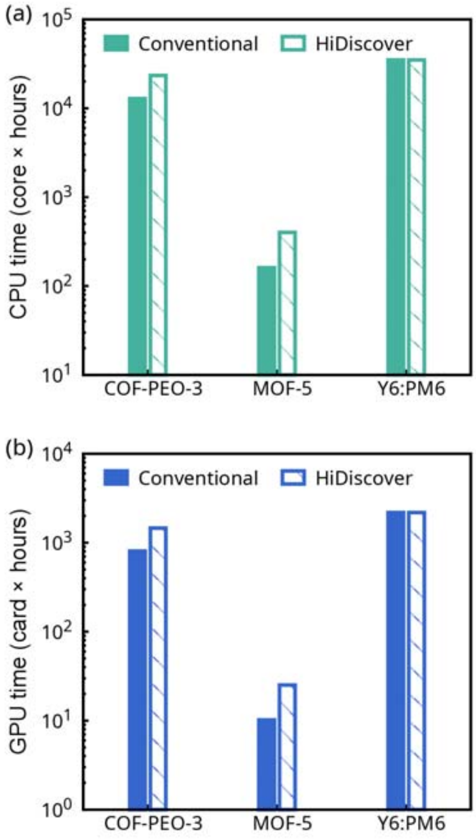

Figure S107. (a) CPU and (b) GPU time of the molecular dynamics (MD) production runs in this study. The times for conventional MD studies were estimated by excluding the new reference molecular systems introduced in the HiDiscover protocol.

Table S3. Summary of the computational times required by the molecular dynamics (MD) production runs in this study.

molecular system	type of system	new molecular models introduced in HiDiscover	CPU hours	GPU hours
MD model 1	reference	yes	83.5	5.2
MD model 2	reference	yes	136.4	8.5
MD model 3	reference	yes	144.5	9.0
MD model 4	reference	yes	3079.5	192.5
MD model 5	reference	yes	2895.9	181.0
MD model 6	reference	yes	3009.3	188.1
MD model 7	reference	yes	1164.5	72.8
MD model 8	reference/target	no	12856.6	803.5
MD model 9	reference	yes	29.3	1.8
MD model 10	reference	yes	26.1	1.6
MD model 11	reference	yes	73.9	4.6
MD model 12	reference	yes	110.9	6.9
MD model 13	reference/target	no	36.2	2.3
MD model 14	reference/target	no	39.5	2.5
MD model 15	reference/target	no	42.4	2.7
MD model 16	target	no	42.6	2.7
MD model 17	reference	yes	0.2	0
MD model 18	reference/target	no	3422.9	213.9
MD model 19	target	no	4196.5	262.3
MD model 20	target	no	5516.9	344.8
MD model 21	target	no	8024.9	501.6
MD model 22	target	no	13781.1	861.3

We note that the length of the MD simulations for the reference systems may impact the accuracy of the trained model and thus the ML-interpreted results. Therefore, one needs to balance the computational cost for the new reference molecular systems and prediction accuracy. To get an insight into these impacts, we performed detailed benchmarks on tasks ζ_A (the one with the most reference systems) and ζ_E (the one with the highest relative computational cost increase compared to a conventional MD study). To approximate the effect of using shorter MD simulations (lower computational cost) for the new reference systems introduced in HiDiscover, we have constructed datasets only from the first 0.05%, 0.1%, 0.2%, 0.5%, 1%, 2%, 5%, 10%, 20%, or 50% portion of their production runs. As can be seen from **Figures S108-S111**, simultaneously high CCRs and stable prediction results on the target systems can be achieved even if we use only ~1% of the MD simulation length for the new reference molecular systems. We note that the full datasets for the 5 new reference systems in task ζ_A correspond to MD production runs of 100 ns, 50 ns, 100 ns, 500 ns, and 1000 ns, respectively. Therefore, 1% of the new reference datasets only use 1-ns, 0.5-ns, 1-ns, 5-ns, and 10-ns MD production runs, respectively. Similarly, 1% of the new reference datasets

in task ζ_E correspond to only 1-ns MD production runs for the 4 new reference systems. These results point out that MD simulations that are much shorter than those considered in this study can be used for the new reference systems. Therefore, the actual computational cost increase when invoking the HiDiscover protocol for mechanistic studies is expected to be much lower than those we present here. When applying the HiDiscover protocol, the datasets of the reference molecular systems can be constructed in a progressive way as the MD simulations are performed. Tests similar to those in **Figures S108-S111** will inform the user whether the data have been sufficient.

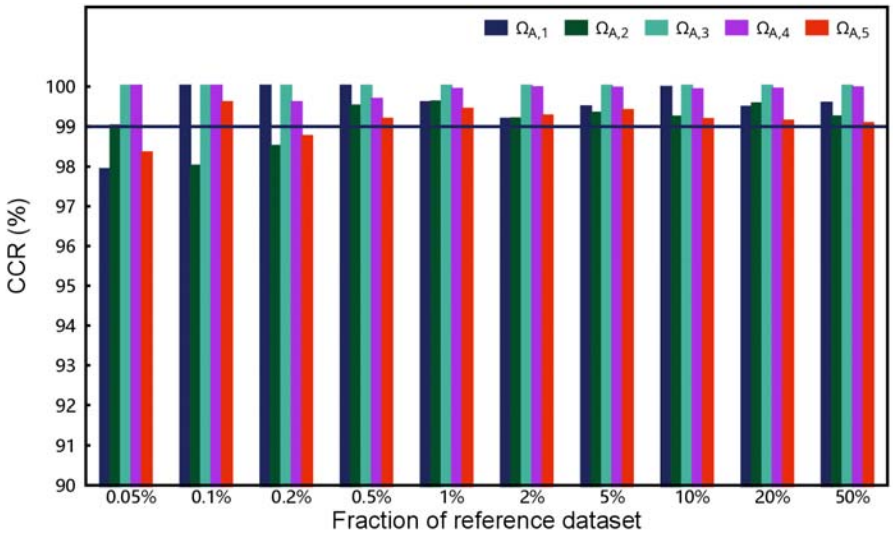

Figure S108. Correct classification rate (CCR) of the model in task ζ_A (refer to **Table 1** for its definition) trained using data from the first 0.05%, 0.1%, 0.2%, 0.5%, 1%, 2%, 5%, 10%, 20%, or 50% portion (approximating shorter molecular dynamics (MD) runs for the reference molecular systems) on the test sets ($\Omega_{A,1}$, $\Omega_{A,2}$, $\Omega_{A,3}$, $\Omega_{A,4}$, and $\Omega_{A,5}$). The solid line shows 99% accuracy.

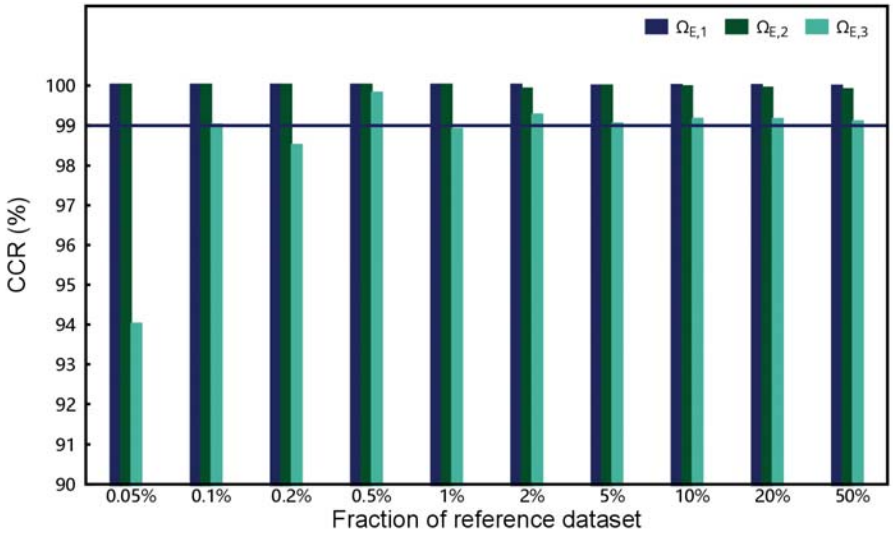

Figure S109. Correct classification rate (CCR) of the model in task ζ_E (refer to the main article for its definition) trained using data from the first 0.05%, 0.1%, 0.2%, 0.5%, 1%, 2%, 5%, 10%, 20%, or 50% portion (approximating shorter molecular dynamics (MD) runs for the reference molecular systems) on the test sets ($\Omega_{E,1}$, $\Omega_{E,2}$, and $\Omega_{E,3}$). The solid line shows 99% accuracy.

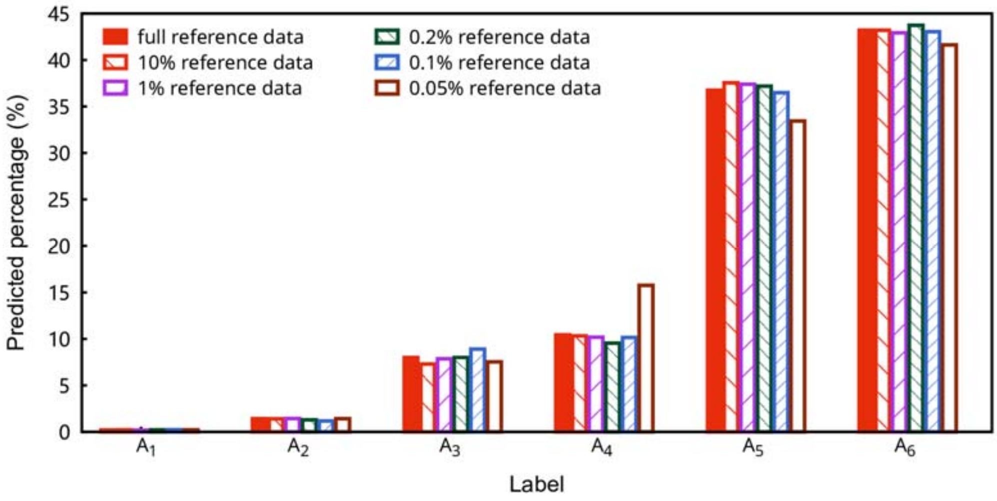

Figure S110. Ratios of predicted labels (among all labels, showing as percentages) on the target system (MD model 8) in task ζ_A (refer to **Table 1** for its definition), applied with models trained using data from the first 0.05%, 0.1%, 0.2%, 1%, or 10% portion (approximating shorter molecular dynamics (MD) runs for the reference molecular systems). The results of the model trained using the full dataset are also shown for comparison. Refer to **Table 1** for definitions of the labels.

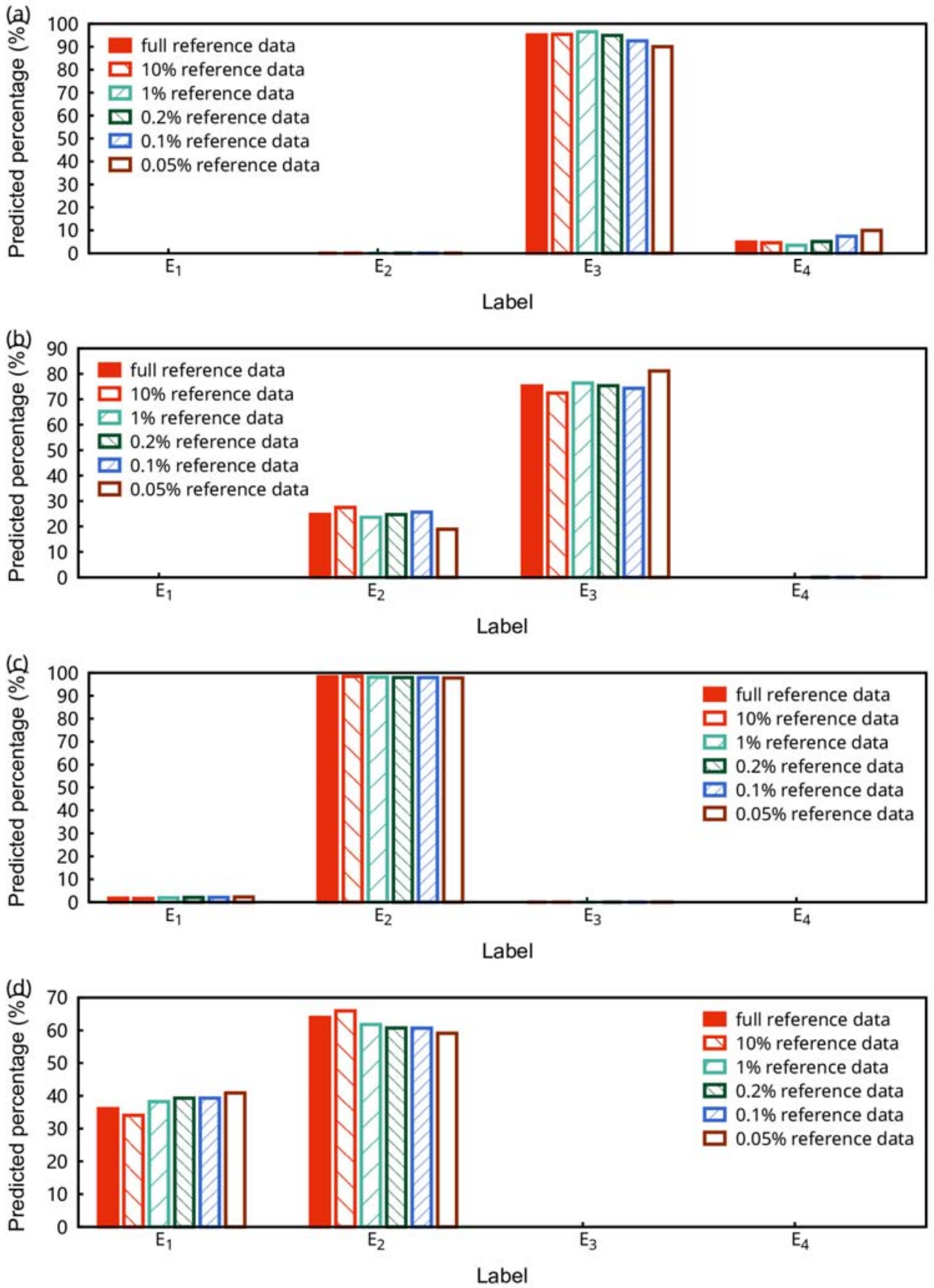

Figure S111. Ratios of predicted labels (among all labels, showing as percentages) on the target molecular dynamics (MD) models (a) 13, (b) 14, (c) 15, and (d) 16 in task ζ_E (refer to the main article for its definition), applied with models trained using data from the first 0.05%, 0.1%, 0.2%, 1%, or 10% portion (approximating shorter MD runs for the reference molecular systems). The results of the model trained using the full dataset are also shown for comparison. Refer to the main article for definitions of the labels E_1, E_2, E_3, and E_4.

5. Additional data analysis

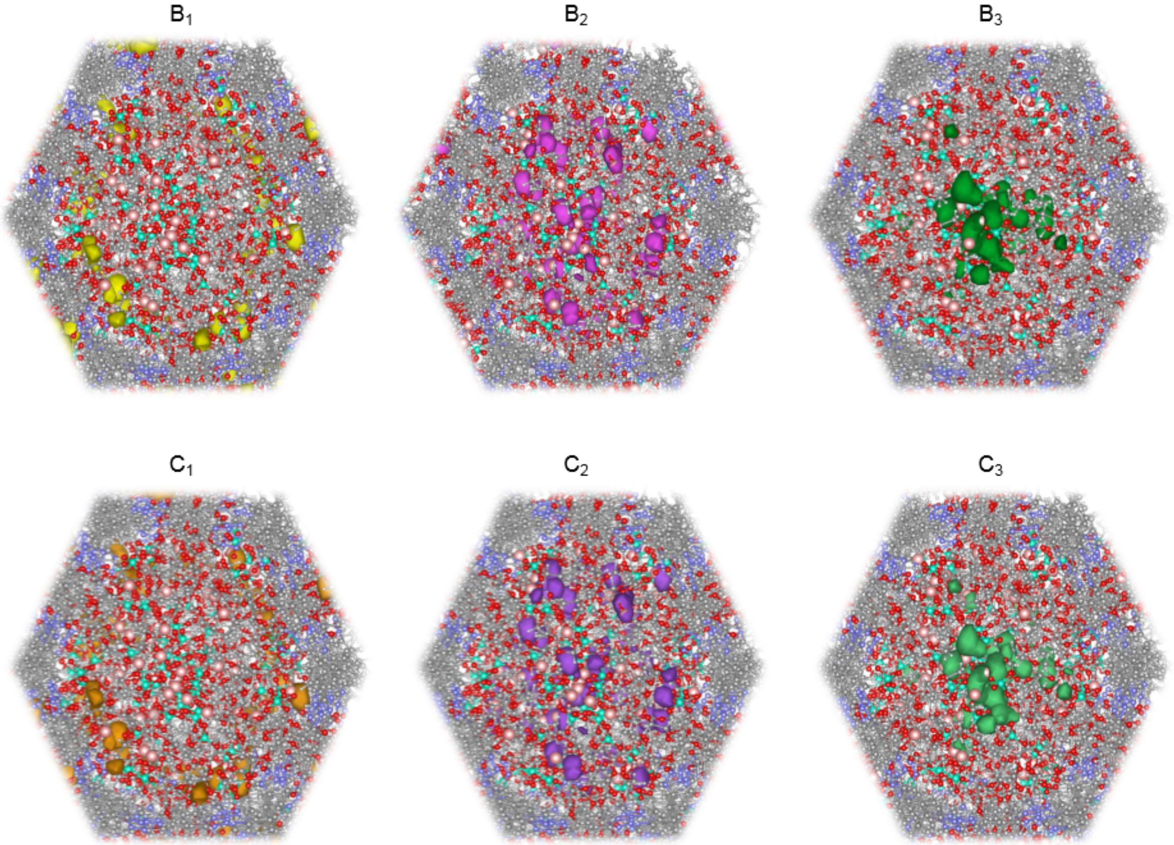

Figure S112. Isosurfaces of the distribution of Li-ions belonging to contexts B_1, B_2, B_3, C_1, C_2, and C_3 (see **Table 1** for their definitions) from one molecular dynamics (MD) simulation. Li-ions in B_1 and C_1 tend to distribute close to the 2D COF pore walls. Those in contexts B_3 and C_3 are mostly located in the center of the pores.

We found that the contexts exhibit correlated patterns, as illustrated in **Figure S113**. For example, B_1 and C_1, B_2 and C_2, and B_3 and C_3 demonstrate strong correlations. This coherence is expected as tasks ζ_B and ζ_C characterize the distances between the Li-ion and the main COF framework. On the other hand, A_1, A_2, A_3, and A_5 are more commonly associated with D_2 than D_1, which is understandable since the tightly bound PEO chains in D_1 would repel ClO_4^-.

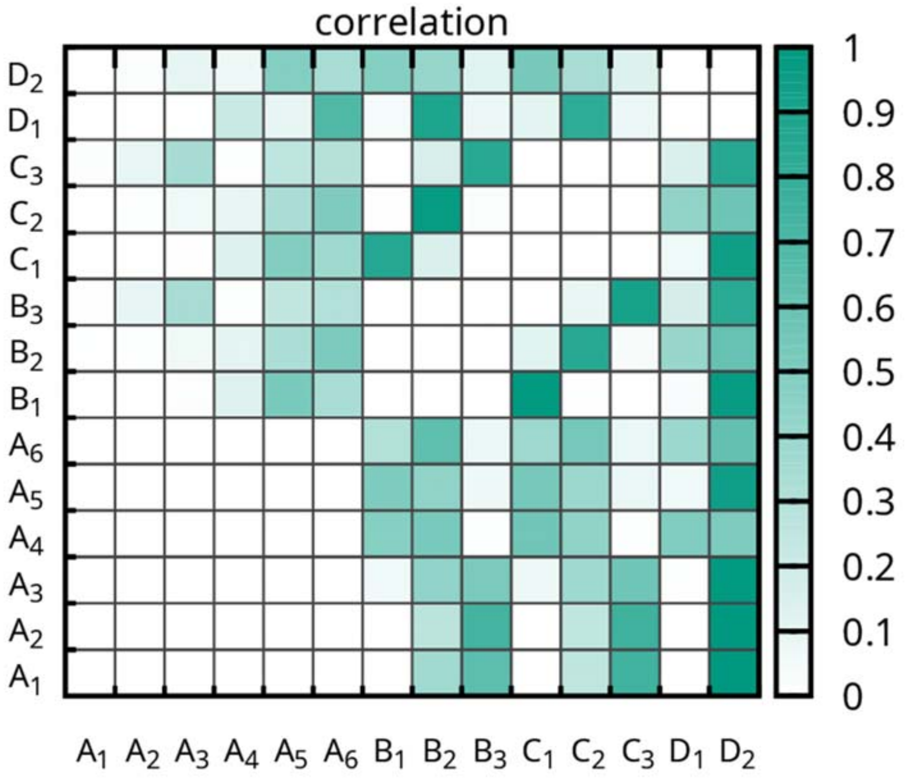

Figure S113. Correlation between different contexts, normalized within each context set in the row columns. For example, the correlation between row A_i and column B_j is $p_{A_iB_j} \Big/ \sum_l p_{A_iB_l}$. Here, i is the row index, j and l are the column indices. Refer to **Table 1** for definitions of the contexts.

Figure S114 illustrates the evolution of a typical Li-ion within the simulated 1-microsecond time frame. We calculated the in-state mean square displacements (MSDs) of all states and shown in **Figure 4b**. Sates associated with contexts B_1 and C_1 have only moderate degrees of movement, as opposed to the often proposed transport pathways on the surface of the 2D COF walls.[6] On the other hand, states (A_3,B_2,C_2,D_2) and (A_3,B_3,C_3,D_2) exhibit the most significant movement along the pore direction. These correspond to Li ions sandwiched between the PEO side chains and the anions (see **Figures S115** and **S116**). The vibrational movement of the side chains and the Coulombic pulling from the asymmetrically located anions could contribute to their large Li-ion motion. However, these two configurations only correspond to ~6% of the total arrangements, indicating that most of the Li ions in COF-PEO-3 transport less efficiently and point to a large room for improvement. For more efficient Li-ion transport, strategies to increase the ratio of these ionic configurations are worth exploring.

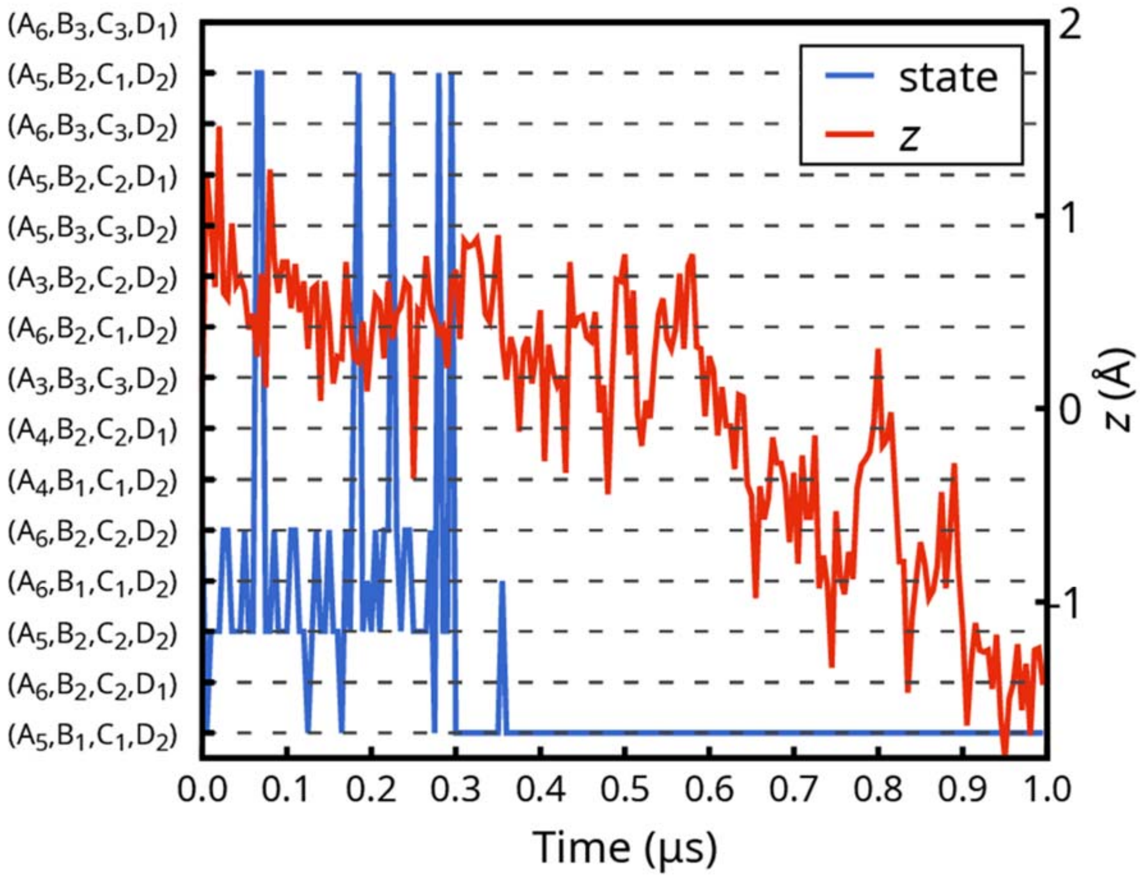

Figure S114. Illustration of the evolution of the state of a Li-ion and its position in the *z*-direction (along the pore channel) at different times. Refer to **Table 1** for definitions of the contexts in a given state.

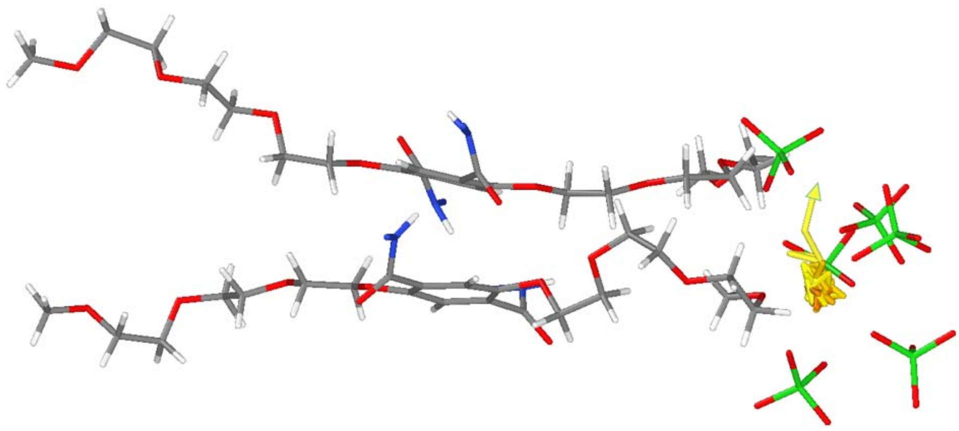

Figure S115. Illustration of Li-ion motion in state (A_3,B_2,C_2,D_2). Yellow-to-white lines with arrow highlight the motion of the Li-ion.

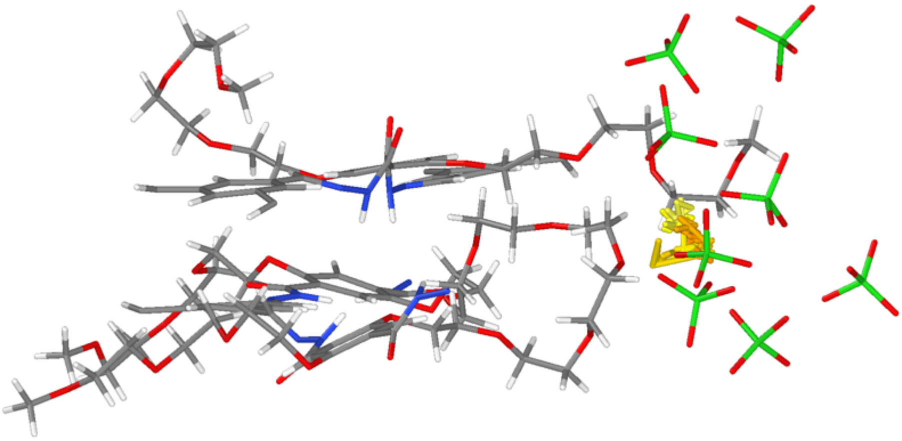

Figure S116. Illustration of Li-ion motion in state (A_3,B_3,C_3,D_2). Yellow-to-white lines with arrow highlight the motion of the Li-ion.

We found that most state transitions are associated with movements considerably faster than the in-state ones (see **Figure S117**). These transitions demonstrate the hopping between the PEO side chains with large movements, exemplified by pairs of (A_6,B_2,C_2,D_1) and (A_6,B_2,C_2,D_2), (A_5,B_2,C_2,D_2) and (A_5,B_2,C_2,D_1), as shown in **Figures S118** and **S119**. This aligns with the mechanisms observed in traditional polymer electrolytes.[7] Here, the 2D COF framework pulls the side chains and competes with their tendencies to form tight contacts with the Li-ion, which could promote the hopping of Li ions out of PEO binding sites. However, this chemical graft approach also restricts the long-range motion of the PEO side chains, causing the movement of the Li-ion with its binding chain to be local vibrations only (see **Figure S120**). Additionally, there are frequent fluctuations of the Li^+-ClO_4^- distances (transitions among A_3-A_6), highlighting the fast motions of ions with respect to each other in the loosely bound PEO matrix (**Figures S121** and **S122**).

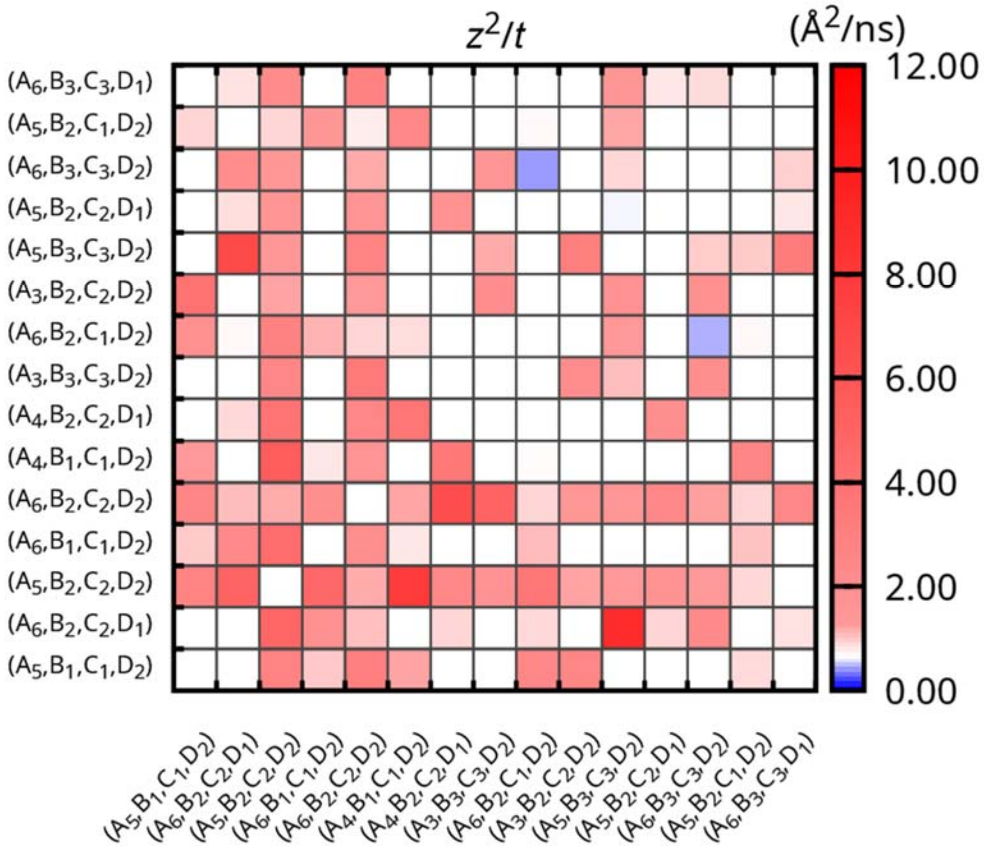

Figure S117. The change speeds of mean square displacements (MSDs) in the *z* direction during state transitions, analyzed by the average value in a 100-ps time window centered at the transition time. Red color highlights the transitions with larger motions than the average in-state movement. Statistically insignificant transitions (fewer than 10 occurrences during the simulations) are represented with pure white color. Refer to **Table 1** for definitions of the contexts in a given state.

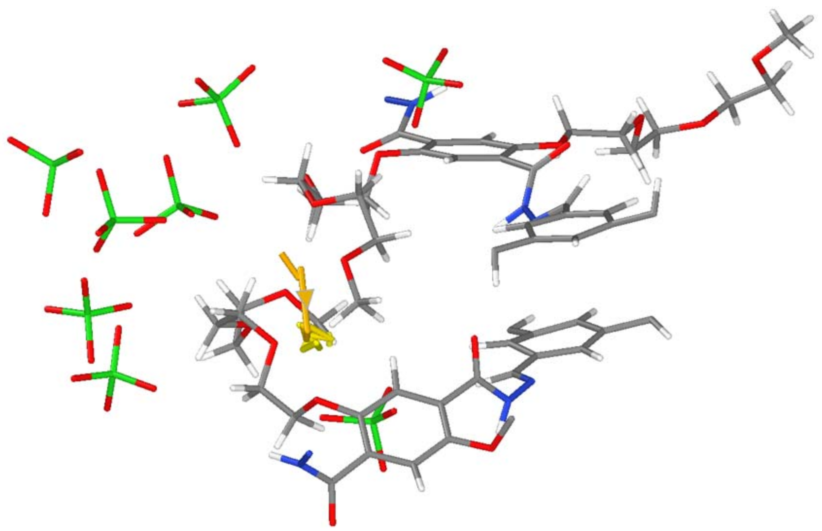

Figure S118. Illustration of Li-ion motion in during the transition between states (A_6,B_2,C_2,D_1) and (A_6,B_2,C_2,D_2). Yellow-to-white lines with arrow highlight the motion of the Li-ion.

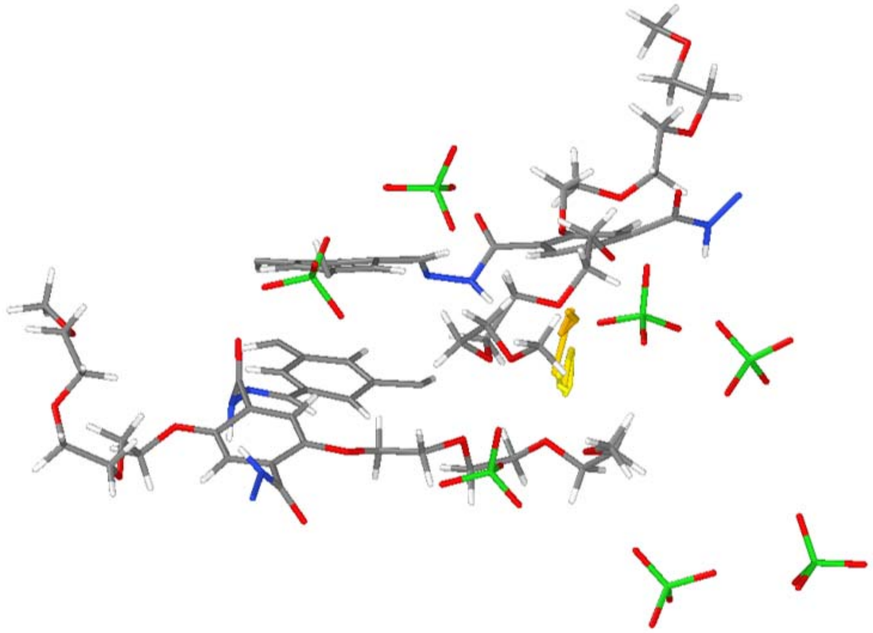

Figure S119. Illustration of Li-ion motion in during the transition between states (A_5,B_2,C_2,D_2) and (A_5,B_2,C_2,D_1). Yellow-to-white lines with arrow highlight the motion of the Li-ion.

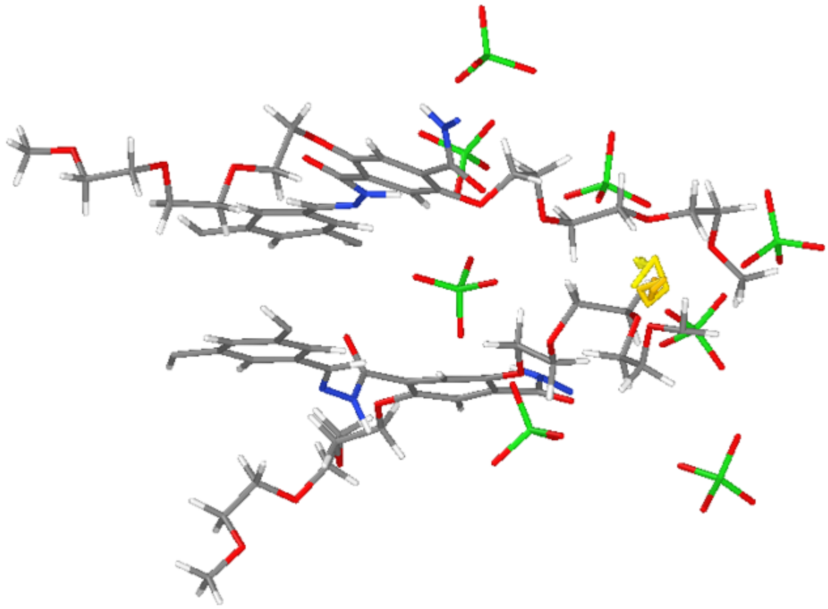

Figure S120. Illustration of Li-ion motion in during the transition between states (A_6,B_2,C_2,D_1) and (A_6,B_3,C_3,D_1). Yellow-to-white lines with arrow highlight the motion of the Li-ion.

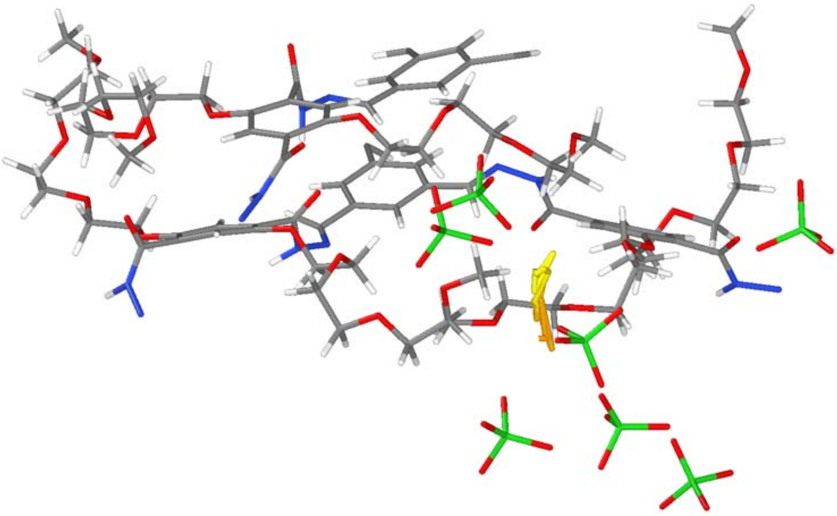

Figure S121. Illustration of Li-ion motion in during the transition between states (A_3,B_2,C_2,D_2) and (A_5,B_2,C_2,D_2). Yellow-to-white lines with arrow highlight the motion of the Li-ion.

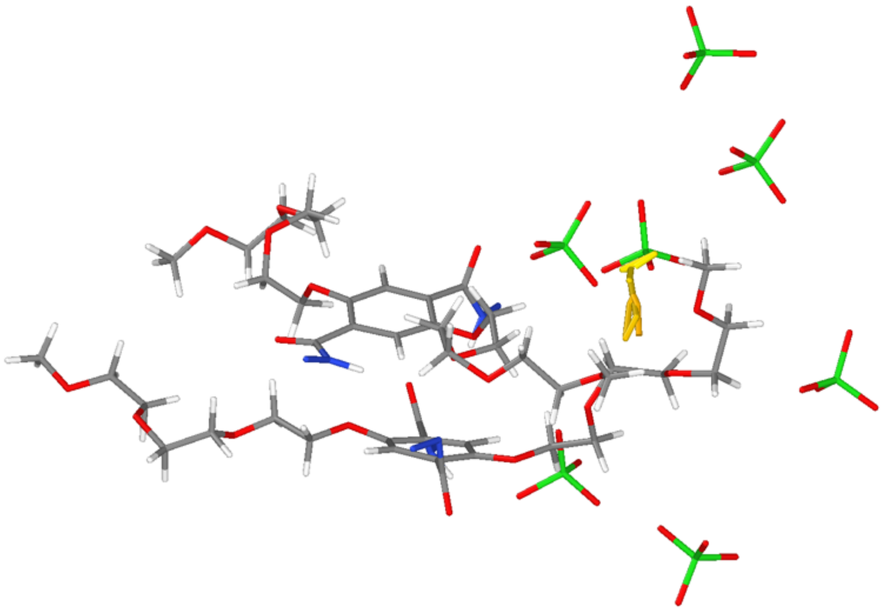

Figure S122. Illustration of Li-ion motion in during the transition between states (A_6,B_2,C_2,D_2) and (A_5,B_2,C_2,D_2). Yellow-to-white lines with arrow highlight the motion of the Li-ion.

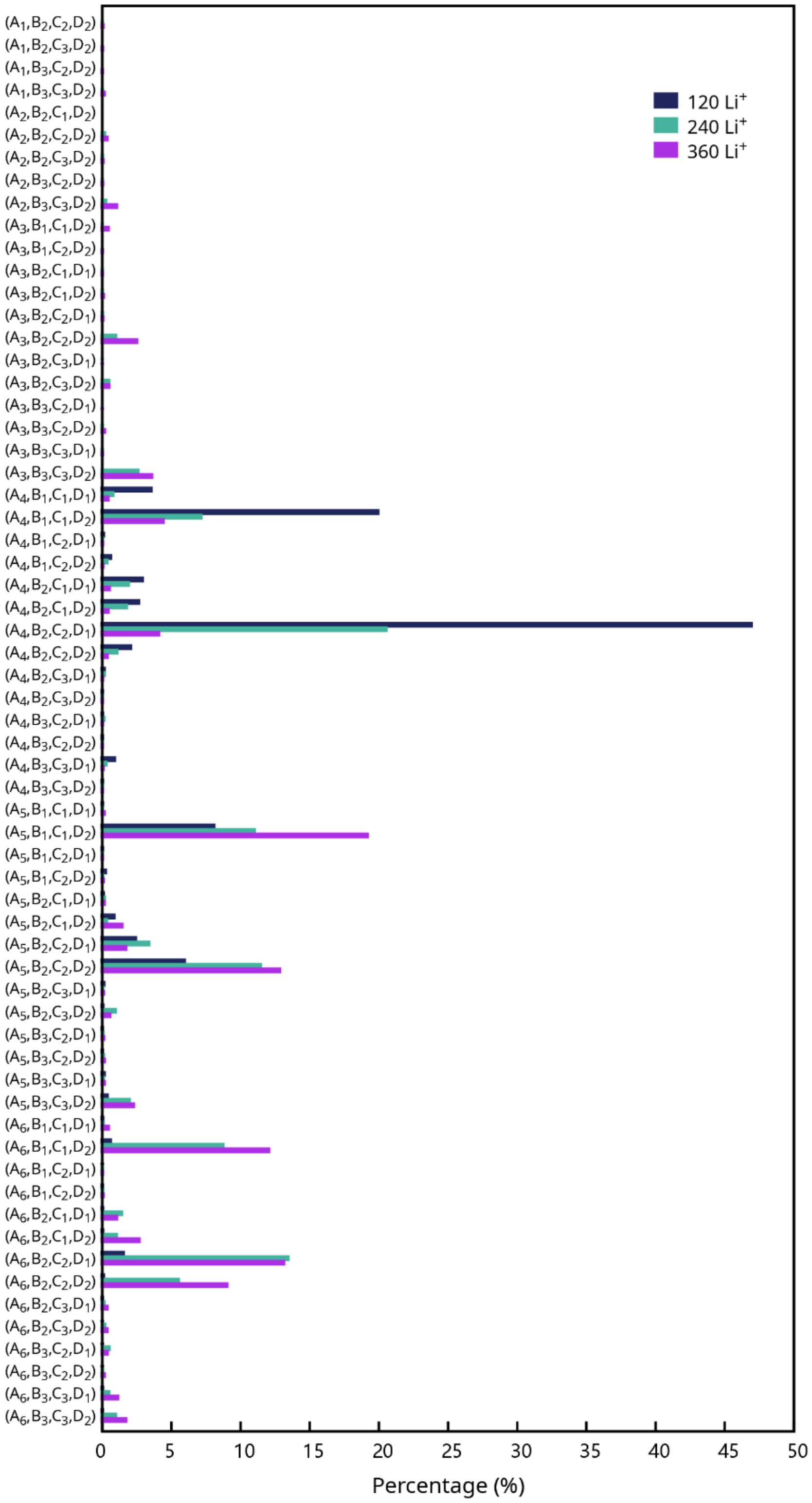

Figure S123. Ratios of different Li-ion state among all states at different concentrations, showing as percentages. The total number of states is 63. Refer to **Table 1** for definitions of the contexts in a given state.

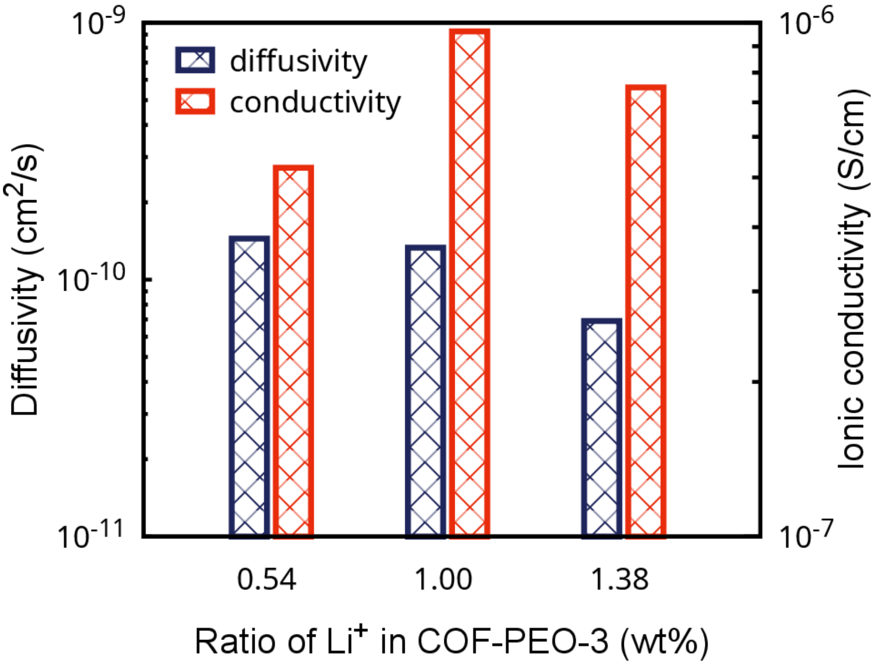

Figure S124. Li-ion diffusivities and ionic conductivities along the pore of COF-PEO-3 at different concentrations of Li^+. To reduce statistical errors, 16, 16, and 8 independent 1 μs-simulations were used for averaging at 0.54wt%, 1.00wt%, and 1.38wt% Li-ion concentrations, respectively.

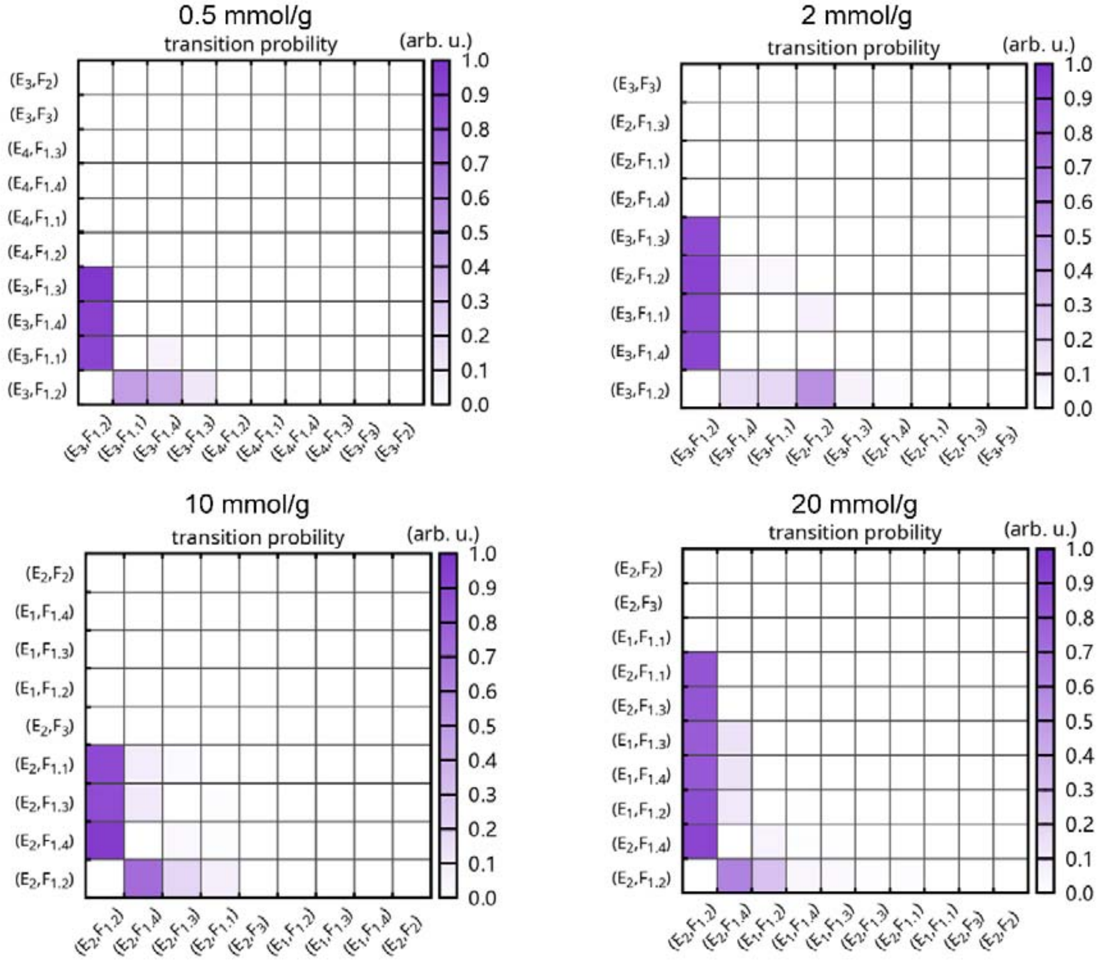

Figure S125. Transition probabilities between various states (p_{ij} corresponds to the value at the ith row and the jth column) at 0.5, 2, 10, and 20 mmol/g CO_2 loadings in MOF-5. The values in each row are normalized. Refer to the main article for definitions of the contexts in a given state.

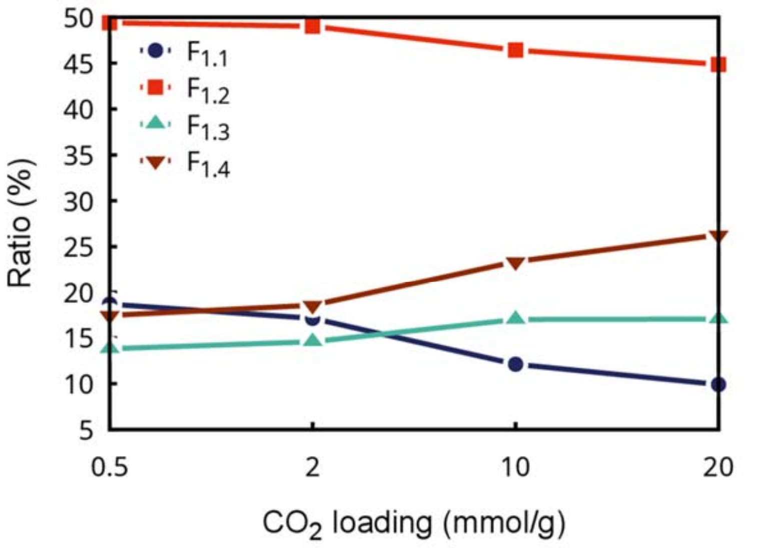

Figure S126. Ratios of the CO_2 configurations corresponding to sub-contexts $F_{1.1}$, $F_{1.2}$, $F_{1.3}$, and $F_{1.4}$ (among all configurations, showing as percentages) for 0.5, 2, 10, and 20 mmol/g CO_2 loading in MOF-5. Sub-contexts $F_{1.1}$, $F_{1.2}$, $F_{1.3}$, $F_{1.4}$ represent coner, off-corner, between-face-center, and through-face-center configurations, respectively.

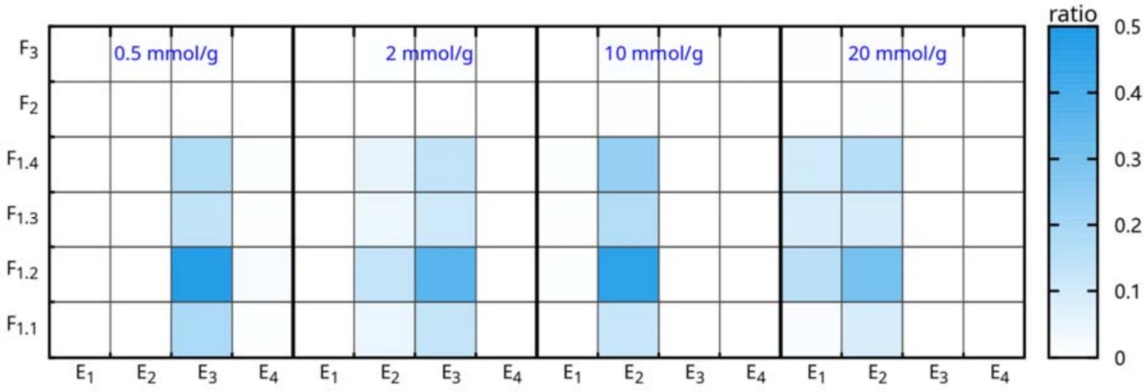

Figure S127. Ratios of all analyzed contexts (among all contexts, showing as percentages) for 0.5, 2, 10, and 20 mmol/g CO_2 loading in MOF-5. Refer to the main article for definitions of the contexts.

6. Suggestions for designing the reference molecular systems

To facilitate the use of the HiDiscover protocol in the study of multi-component materials, we have provided reference system suggestions for common cases. In particular, one can build single-component, two-component, three-component, ..., systems corresponding to the target material (**Table S4**). Different phases, temperatures, pressures, and aggregation states can be further used. These systems can generally be constructed via molecular modeling and are usually easier to model than the more complex target material. The selection of the reference systems is often clear when the problem to study is determined by the researcher. For example, if we focus on intermolecular arrangements of Y6 in a Y6:PM6 heterojunction in the context of organic solar cells, variations involving the gas state obviously become irrelevant as they are unlikely to be similar to molecular arrangements in the target material (we note that such background knowledge is expected for the researcher even in a conventional MD study). On the other hand, if we would like to differentiate the molecular arrangements in crystalline and amorphous packings, we must include corresponding reference systems as we did in this study.

Table S4. Suggestions for designing the reference molecular systems for multi-component materials.

reference system suggestion	variation
single component from the target system	gas, liquid, or solid phases
	different pressures
	different temperatures
	crystalline or amorphous packing for the solid phase
	different crystalline types for the solid phase
	clusters or slabs for liquid or solid phases
A_xB_y: two-component mixtures from the target system	different mixing ratios
	similar variations to single-component reference systems
$A_xB_yC_z$: three-component mixtures from the target system (for target system with three or more components)	different x:y:z ratios
	similar variations to single-component reference systems
...	...

7. References

1. Shao, J., Tanner, S. W., Thompson, N. & Cheatham, T. E. Clustering Molecular Dynamics Trajectories: 1. Characterizing the Performance of Different Clustering Algorithms. *J. Chem. Theory Comput.* **3**, 2312–2334 (2007).

2. Glielmo, A. *et al.* Unsupervised Learning Methods for Molecular Simulation Data. *Chem. Rev.* **121**, 9722–9758 (2021).
3. Hartigan, J. A. & Wong, M. A. Algorithm AS 136: A k-means clustering algorithm. *J. R. Stat. Soc. Ser. C Appl. Stat.* **28**, 100–108 (1979).
4. Xu, D. & Tian, Y. A comprehensive survey of clustering algorithms. *Ann. Data Sci.* **2**, 165–193 (2015).
5. Martínez, L., Andrade, R., Birgin, E. G. & Martínez, J. M. PACKMOL: A package for building initial configurations for molecular dynamics simulations. *J. Comput. Chem.* **30**, 2157–2164 (2009).
6. Zhang, H. *et al.* Charge and mass transport mechanisms in two-dimensional covalent organic frameworks (2D COFs) for electrochemical energy storage devices. *Energy Environ. Sci.* **16**, 889–951 (2023).
7. Brooks, D. J., Merinov, B. V., Goddard, W. A. I., Kozinsky, B. & Mailoa, J. Atomistic Description of Ionic Diffusion in PEO–LiTFSI: Effect of Temperature, Molecular Weight, and Ionic Concentration. *Macromolecules* **51**, 8987–8995 (2018).
