# Supplementary material for: Hierarchical incremental learning deciphers molecular arrangements in multi-component materials
Source: Nat Commun. 2025 Oct 22;16:9324. doi: 10.1038/s41467-025-64372-4 (PMC12546701; doi:10.1038/s41467-025-64372-4)
Supplement: Supplementary file 2 — Reporting Summary [file 41467_2025_64372_MOESM2_ESM.pdf]

Corresponding author(s): Haoyuan Li
Last updated by author(s): Aug 15, 2025

Reporting Summary

Nature Portfolio wishes to improve the reproducibility of the work that we publish. This form provides structure for consistency and transparency in reporting. For further information on Nature Portfolio policies, see our Editorial Policies and the Editorial Policy Checklist.

Statistics

For all statistical analyses, confirm that the following items are present in the figure legend, table legend, main text, or Methods section.

n/a	Confirmed	
☐	☒	The exact sample size (*n*) for each experimental group/condition, given as a discrete number and unit of measurement
☒	☐	A statement on whether measurements were taken from distinct samples or whether the same sample was measured repeatedly
☒	☐	The statistical test(s) used AND whether they are one- or two-sided<br>*Only common tests should be described solely by name; describe more complex techniques in the Methods section.*
☒	☐	A description of all covariates tested
☒	☐	A description of any assumptions or corrections, such as tests of normality and adjustment for multiple comparisons
☐	☒	A full description of the statistical parameters including central tendency (e.g. means) or other basic estimates (e.g. regression coefficient) AND variation (e.g. standard deviation) or associated estimates of uncertainty (e.g. confidence intervals)
☒	☐	For null hypothesis testing, the test statistic (e.g. *F*, *t*, *r*) with confidence intervals, effect sizes, degrees of freedom and *P* value noted<br>*Give P values as exact values whenever suitable.*
☒	☐	For Bayesian analysis, information on the choice of priors and Markov chain Monte Carlo settings
☐	☒	For hierarchical and complex designs, identification of the appropriate level for tests and full reporting of outcomes
☒	☐	Estimates of effect sizes (e.g. Cohen's *d*, Pearson's *r*), indicating how they were calculated

Our web collection on statistics for biologists contains articles on many of the points above.

Software and code

Policy information about availability of computer code

Data collection	Gromacs (2021.5)<br>packmol (20.3.5)
Data analysis	Gromacs (2021.5)<br>Custom code (uploaded to https://github.com/ShuSmeMat/HiDiscover and https://www.doi.org/10.5281/zenodo.16882233)

For manuscripts utilizing custom algorithms or software that are central to the research but not yet described in published literature, software must be made available to editors and reviewers. We strongly encourage code deposition in a community repository (e.g. GitHub). See the Nature Portfolio guidelines for submitting code & software for further information.

Data

Policy information about availability of data

All manuscripts must include a data availability statement. This statement should provide the following information, where applicable:

- Accession codes, unique identifiers, or web links for publicly available datasets
- A description of any restrictions on data availability
- For clinical datasets or third party data, please ensure that the statement adheres to our policy

Samples of the datasets data generated in this study have been deposited in the Zenodo database under accession code 10.5281/zenodo.132928010 [https://www.doi.org/10.5281/zenodo.13292810]. The initial and final configurations of the molecular dynamics trajectories in this study have been deposited in the Zenodo

database under accession code 10.5281/zenodo.16880925 [https://www.doi.org/10.5281/zenodo.16880925].

Research involving human participants, their data, or biological material

Policy information about studies with human participants or human data. See also policy information about sex, gender (identity/presentation), and sexual orientation and race, ethnicity and racism.

Reporting on sex and gender	n/a
Reporting on race, ethnicity, or other socially relevant groupings	n/a
Population characteristics	n/a
Recruitment	n/a
Ethics oversight	n/a

Note that full information on the approval of the study protocol must also be provided in the manuscript.

Field-specific reporting

Please select the one below that is the best fit for your research. If you are not sure, read the appropriate sections before making your selection.

☐ Life sciences ☐ Behavioural & social sciences ☒ Ecological, evolutionary & environmental sciences

For a reference copy of the document with all sections, see nature.com/documents/nr-reporting-summary-flat.pdf

Ecological, evolutionary & environmental sciences study design

All studies must disclose on these points even when the disclosure is negative.

Study description	This study presents a hierarchical incremental learning research protocol named HiDiscover. It expedites the mechanistic exploration in multi-component materials. As illustrations, we apply this protocol to study Li-ion transport and gas adsorption in nanoporous framework materials, as well as molecular packing in organic active layers for photovoltaics.
Research sample	We performed three series of simulations. The first is COF-PEO-3 electrolyte for studying Li-ion transport within it. The second is MOF-5, which is used to illustrate the configuration of CO2 gas within it. The third is Y6:PM6 blends, used to show the molecular arrangement of molecules in organic solar cells.
Sampling strategy	We have tested that the sampling is sufficient in the Supporting Information. By our test, using ~1% of the data achieves similar results.
Data collection	Data was obtained from computer simulations by Hanyin Zhang and Haoyuan Li.
Timing and spatial scale	The simulations were run on our computing cluster whenever resource is available. The data collection starts in 2022 and ends in 2024.
Data exclusions	No data were excluded.
Reproducibility	Multiple simulations were performed for each system. Results such as density and temperature converge as shown in the Supporting Information.
Randomization	n/a<br>All data were obtained by molecular simulations.
Blinding	n/a<br>All data were obtained by molecular simulations.

Did the study involve field work? ☐ Yes ☒ No

Reporting for specific materials, systems and methods

We require information from authors about some types of materials, experimental systems and methods used in many studies. Here, indicate whether each material, system or method listed is relevant to your study. If you are not sure if a list item applies to your research, read the appropriate section before selecting a response.

Materials & experimental systems

n/a	Involved in the study
☒	☐ Antibodies
☒	☐ Eukaryotic cell lines
☒	☐ Palaeontology and archaeology
☒	☐ Animals and other organisms
☒	☐ Clinical data
☒	☐ Dual use research of concern
☒	☐ Plants

Methods

n/a	Involved in the study
☒	☐ ChIP-seq
☒	☐ Flow cytometry
☒	☐ MRI-based neuroimaging

Plants

Seed stocks	n/a
Novel plant genotypes	n/a
Authentication	n/a
